# Cellular heterogeneity of hepatic granuloma formation and evolution in murine schistosomiasis japonica

**DOI:** 10.1038/s41467-026-75112-7

**Published:** 2026-07-08

**Authors:** Jing Wu, Guangxu Cao, Jiakai Yao, Linzhu Li, Yusen Xiang, Xiaolin Zhong, Yicong Liu, Jingxuan Wang, Lan Yin, Li Shen, Fang Li, Xuan Chen, Jipeng Wang, Jun Cao, Qingfeng Zhang, Yang Dai, Xindong Xu

**Affiliations:** 1 Laboratory of Molecular Parasitology, State Key Laboratory of Cardiology and Research Center for Translational Medicine, Shanghai East Hospital; Key Laboratory of Pathogen-Host Interaction (Tongji University), Ministry of Education; Clinical Center for Brain and Spinal Cord Research, School of Medicine, Tongji University, Shanghai, China; 2 Department of Gynecology, School of Medicine, Shanghai East Hospital, Tongji University, Shanghai, China; 3 National Health Commission Key Laboratory of Parasitic Disease Control and Prevention, Jiangsu Provincial Key Laboratory on Parasite and Vector Control Technology, Jiangsu Institute of Parasitic Diseases, Wuxi, China; 4 School of Basic Medicine, Nanchang Medical College, Nanchang, China; 5 State Key Laboratory of Genetic Engineering, Ministry of Education Key Laboratory of Contemporary Anthropology, School of Life Sciences, Fudan University, Shanghai, China

**Keywords:** Parasite host response, Infection

## Abstract

Schistosomiasis morbidity and mortality are primarily driven by egg-triggered granulomas with subsequent fibrosis. However, the dynamics of cellular composition within these granulomas, particularly the identity and function of key immune cells that orchestrate their formation and evolution, remain poorly understood. Here, we combine single-cell RNA sequencing with multiplex immunofluorescence to characterize the hepatic immune landscape in *Schistosoma japonicum*-infected mice and further validate the functional roles of two neutrophil subsets. We find that eggs alter hepatic immune cell composition, which is characterized by extensive neutrophil recruitment and differentiation. Neutrophil recruitment correlates with CXCL2 derived from both an autocrine loop and paracrine signaling from monocytes. Cellular localization analysis reveals that granulomas progress through three distinct developmental stages: early Ly6G^hi^F4/80^lo^Desmin^lo^, developing Ly6G^mid^F4/80^hi^Desmin^mid^, and advanced Ly6G^lo^F4/80^mid^Desmin^hi^. Neutrophil subsets display a zonal distribution within granulomas, with CD177^+^ neutrophils surrounding the eggs and Ltf^+^ neutrophils localizing to the mid-outer layer. *Cd177* knockdown reduces granuloma size and fibrosis, whereas *Ltf* suppression increases these pathological features, indicating that CD177 and LTF exert opposite effects on granuloma formation. Our findings provide new insights into the cellular complexity of *S. japonicum* egg-induced granulomas and may help guide the development of novel therapies for liver fibrosis.

## Abstract (web summary)

Schistosoma japonicum infection causes severe liver damage. Here, the authors reveal that the infection recruits abundant liver neutrophils, with distinct subsets exhibiting zonal distribution within granulomas and playing diverse biological roles.

## Introduction

Schistosomiasis is one of the most prevalent helminthic diseases, affecting over 250 million people worldwide^1–3^. It is primarily caused by *Schistosoma japonicum*, *Schistosoma mansoni*, and *Schistosoma haematobium*. *S. japonicum* and *S. mansoni* cause hepatosplenic and intestinal schistosomiasis, with parasitic worms residing in the mesenteric veins, whereas *S. haematobium* inhabits the venous plexus of the urinary bladder and causes urogenital schistosomiasis^4^. Over the past five decades, significant progress has been made in schistosomiasis control, primarily through mass drug administration (MDA) of praziquantel to entire at-risk populations^5,6^. However, preventive chemotherapy alone is insufficient to interrupt the transmission cycle^7^. Reinfection remains a major global health burden and contributes to long-term complications such as hepatic fibrosis, splenomegaly, and anemia.

The *Schistosoma* lifecycle involves a freshwater snail as the intermediate host and a mammalian definitive host^4^. In humans, contact with water containing cercariae results in rapid skin penetration and infection. Acute schistosomiasis, also known as Katayama syndrome, primarily affects non-immune individuals, such as those who have never been exposed previously or who are exposed to a large number of cercariae^8^. The disease typically manifests 2 weeks to 3 months after infection and is driven by a systemic immune response triggered by migrating schistosomulae^9^. In the chronic stages of schistosomiasis, long-term complications arise from the hosts immune response to parasite eggs trapped in tissues, leading to granulomatous inflammation and fibrosis^10^. During the chronic stages of hepatosplenic and intestinal schistosomiasis, one-half to two-thirds of the eggs deposited in the mesenteric venules travel through the circulation to multiple organs, with the majority lodging in the liver^11^. In the liver, granulomatous inflammation around eggs and subsequent fibrosis are the primary pathological changes seen in *S. japonicum* and *S. mansoni* infections^4,12^.

The granulomatous response to *Schistosoma* eggs is a complex immune reaction that both protects the host and has pathological consequences. The granulomatous response is crucial for host survival^13^. Granulomas act as a barrier by encapsulating eggs and neutralizing hepatotoxic secretions, protecting surrounding tissues such as hepatocytes from damage and preventing acute, fatal disease^14^. However, the granulomatous response often leads to excessive inflammation and fibrosis, thus contributing to the majority of the morbidity and mortality associated with schistosomiasis^15,16^. Granulomas promote collagen deposition via IL-13–driven activation of M2 macrophages and fibroblasts, leading to hepatic and periportal fibrosis^17,18^. Fibrosis in the liver portal tract often leads to the formation of obstructive portal lesions and the development of portal hypertension, and may result in gastrointestinal bleeding and liver failure^19^.

Schistosome granulomas recruit a wide variety of immune cells, including eosinophils, neutrophils, monocytes, macrophages, lymphocytes, and epithelioid cells, with variations in immune cell composition differing across time and species. In *S. mansoni* infection, eosinophils predominate as the major immune cell type, and this phenomenon is facilitated by Th2-mediated inflammatory mechanisms^20,21^. Although eosinophils are also a prevalent feature in clinical *S. japonicum* infection, in severe cases, there is often a marked increase in neutrophils accompanied by a decrease or even complete absence of eosinophils^22^. In addition, neutrophils predominate in the acute initial inflammatory stage of granuloma development in mice and monkeys infected with *S. japonicum*^23,24^. As *S. japonicum* infection in mice advances, late-stage granulomas demonstrate a transition characterized by a decrease in neutrophils and macrophages, while lymphocyte-like cells and plasma cells become more prominent at the periphery of periportal inflammation. However, neutrophils continue to be the predominant cell type within advanced *S. japonicum* granulomas, frequently degranulating upon contact with eggshells and occasionally triggering immune responses inside the schistosome eggs^22^. These findings underscore a key distinction between *S. japonicum* and *S. mansoni*, in that *S. japonicum* granulomas are characterized by a higher ratio of neutrophils and acute inflammation, while *S. mansoni* granulomas are eosinophil-dominated and Th2-driven. Despite these well-documented species-specific differences, a precise understanding of the temporal and spatial dynamics, functional specialization, intercellular crosstalk, and phenotypic plasticity of immune cell subsets during granuloma initiation, progression, and resolution remains elusive, posing a critical obstacle to the development of targeted antifibrotic therapies.

To better understand the dynamic pathological changes in the liver caused by *S. japonicum* infection, we combined single-cell RNA sequencing with multiplex immunofluorescence, histopathological analysis, and serological profiling to comprehensively characterize the hepatic immune landscape in a murine model of *S. japonicum* infection. Furthermore, we validated the functional roles of two neutrophil subsets in the regulation of granuloma formation through targeted marker gene knockdown. Our findings reveal remarkable heterogeneity in the cellular composition, spatial organization, and molecular signatures of egg-induced granulomas, providing critical mechanistic insights for identifying novel therapeutic targets against schistosomiasis-associated hepatic fibrosis.

## Results

### Mouse body weight, serum markers, and hepatic fibrosis over the course of *S. japonicum* infection

To investigate temporal pathological changes induced by *S. japonicum* infection, C57BL/6J mice were infected with 30 ± 2 cercariae (Supplementary Fig. 1a). Body weights, levels of alanine transaminase (ALT), aspartate transaminase (AST), albumin (ALB), and the severity of hepatic fibrosis were examined throughout infection.

From 0 to 22 d.p.i., no significant alterations in body weight or serum markers (ALT, AST, and ALB) were observed compared with uninfected controls (*P* > 0.05) (Supplementary Fig. 1b, d, e). Hematoxylin and Eosin (H&E) staining and Masson’s trichrome staining showed minimal inflammatory cell infiltration and collagen deposition surrounding migrating schistosomula, with fibrosis accounting for <1.7% of the liver area (Supplementary Fig. 1f–h). Hepatic pathological lesions intensified following the onset of egg deposition at 28 d.p.i. Egg granulomas exhibited pronounced inflammatory cell infiltrates and collagen deposition, with fibrosis increasing to 4.4 ± 0.8% of the total liver area (*P* < 0.001) (Supplementary Fig. 1f–h). Although body weight continued to increase, serum ALT and AST levels were significantly elevated compared with controls, indicating that early liver dysfunction was associated with egg deposition (Supplementary Fig. 1b, d, e).

Between 35 and 56 d.p.i., persistent egg deposition led to significant and rapid body weight loss and further elevated serum ALT and AST levels, as well as inducing a marked decrease in ALB levels, reflecting severe hepatic dysfunction (Supplementary Fig. 1b, d, e). Approximately 20% of the mice died between 42 and 56 d.p.i. (Supplementary Fig. 1c). Histologically, inflammatory cell infiltration around the eggs intensified, with granulomas occupying 33.8 ± 3.6% and 51.7 ± 5.1% of total liver area at 42 and 56 d.p.i., respectively (Supplementary Fig. 1f–h). Prominent areas of necrotic tissue were observed within granulomas at 56 d.p.i. In addition, Ki-67, osteopontin (OPN), α-smooth muscle actin (α-SMA), and collagen I levels were highly expressed around granulomas, and their expression was diffusely distributed throughout the liver (Supplementary Fig. 2a–d). This expression pattern suggests that coordinated interplay among immune cell proliferation (Ki-67), inflammatory remodeling (OPN), hepatic stellate cell activation (α-SMA), and extracellular matrix deposition (collagen I) contributes to granuloma progression and hepatic fibrosis.

From 63 to 70 d.p.i., the mouse body weights began to recover, accompanied by decreased serum ALT and AST levels and increased ALB levels, indicating partial restoration of hepatic function (Supplementary Fig. 1b, d, e). Concurrently, reductions in granuloma size and fibrosis severity were observed, with the granulomatous area accounting for 29.4 ± 4.0% of the total liver area by 70 d.p.i. (Supplementary Fig. 1f–h).

These findings delineate three distinct phases of *S. japonicum*-induced pathology in mice (Supplementary Fig. 1a): (1) an asymptomatic pre-egg phase (0 to 28 d.p.i.), characterized by mild inflammation; (2) an acute granuloma formation phase (28 to 56 d.p.i.), marked by severe liver pathology and mortality; and (3) a chronic phase (56 to 70 d.p.i.), characterized by partial restoration of hepatic function and resolution of pathological lesions. These stages provided a framework for our subsequent mechanistic exploration of *S. japonicum* pathogenesis in mice.

### Single-cell RNA sequencing analysis of liver NPCs in mice infected with *S. japonicum*

**Fig. 1:**
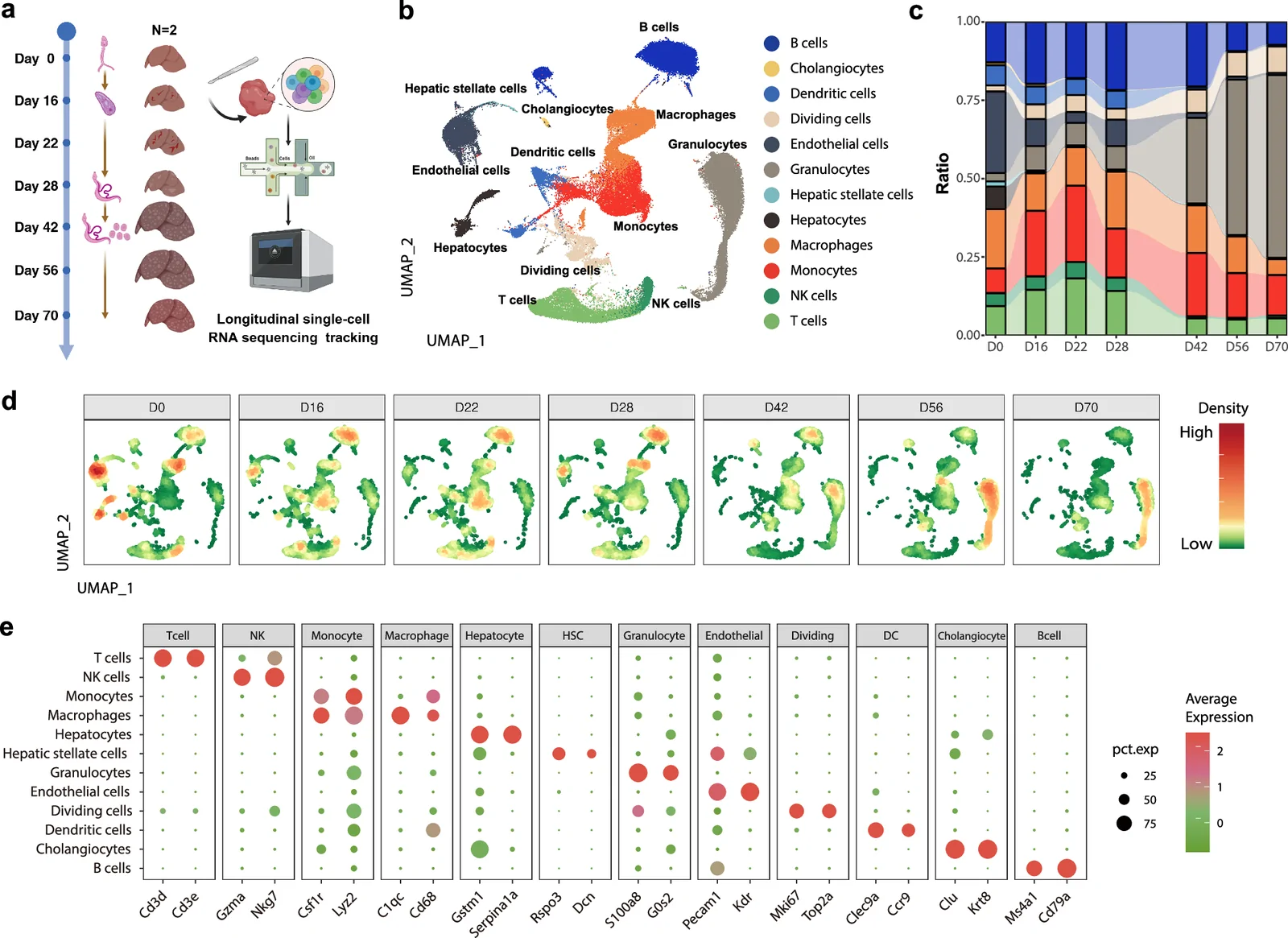
Study design and single-cell transcriptomic profiling of liver non-parenchymal cells (NPCs) in *S. japonicum*–infected mice. **a** Schematic of the longitudinal study design: liver NPCs were collected at seven time points (0–70 d.p.i.), with two biological replicates per time point, followed by scRNA-seq analysis (Created in BioRender. Cao, G. (2026) https://BioRender.com/v5bk78q). **b** UMAP clustering of 87,498 liver NPCs identified 12 distinct cell types. Each dot represents a single cell, and each color indicates an annotated cell type. **c** Temporal dynamics of cell type proportions throughout the course of infection (y-axis: relative proportion of each cell type; x-axis: days post-infection). **d** UMAP projections showing the liver NPC distribution at each time point. **e** Dot plot of the top two marker genes per cell type. Dot color indicates average gene expression level, and dot size represents the percentage of cells expressing the gene in each cluster.

To elucidate the heterogeneity of liver non-parenchymal cells (NPCs) and their dynamic changes during infection, we performed scRNA-seq analysis of NPCs isolated from mice infected with *S. japonicum* (Fig. 1a). A total of 87,498 single-cell transcriptomes were obtained at seven time points from 0 to 70 d.p.i. (*n* = 2 per time point). Following quality control to exclude apoptotic cells, multiplets, and duplicates, a total of 3756 to 8936 high-quality single cells per mouse were retained for subsequent sequencing and analysis. The lowest cell counts were observed at 70 d.p.i. (3756 and 3761, *n* = 2), likely because severe liver fibrosis interfered with isolating single cells at this stage. Uniform manifold approximation and projection (UMAP) identified transcriptomic profiles based on classic marker gene expression for 12 major cell types, corresponding to B cells (14,050), cholangiocytes (364), dendritic cells (DCs) (3584), endothelial cells (7806), granulocytes (16,440), hepatic stellate cells (HSCs) (48), hepatocytes (1514), macrophages (12,379), monocytes (14,367), NK cells (2829), T cells (9615), and dividing cells (4502) (Fig. 1b, e). In the UMAP space, macrophages were spatially adjacent to monocytes, T cells were proximal to NK cells, and other cell types were well separated. The granulocyte cluster was composed of 97.7% neutrophils and approximately 2.3% mast cells, with no eosinophils detected.

Next, we performed dynamic cell proportion analysis of the identified cell types. The numbers of T cells and B cells increased during the asymptomatic phase of infection but decreased in the acute and chronic phases, while granulocyte numbers persistently rose, with granulocytes becoming the predominant cellular component during the acute and chronic phases. The proportions of monocytes and macrophages remained stable across all stages of infection, but very few DCs, NK cells, and endothelial cells were detected in the acute and chronic phases (Fig. 1c, d). HSCs were scarce, likely because they are encapsulated by extracellular matrix components, such as collagen, and were presumably filtered out during single-cell preparations. The small number of hepatocytes that we detected might have been left over from NPC preparation. Taken together, these data reveal a profound and stage-specific remodeling of the hepatic immune cells. This restructuring is characterized by robust granulocyte expansion (predominantly neutrophils), which are highly involved in shaping the inflammatory response and subsequent pathological progression throughout *S. japonicum* infection.

### Egg-driven preferential neutrophil response in the liver during *S. japonicum* infection

**Fig. 2:**
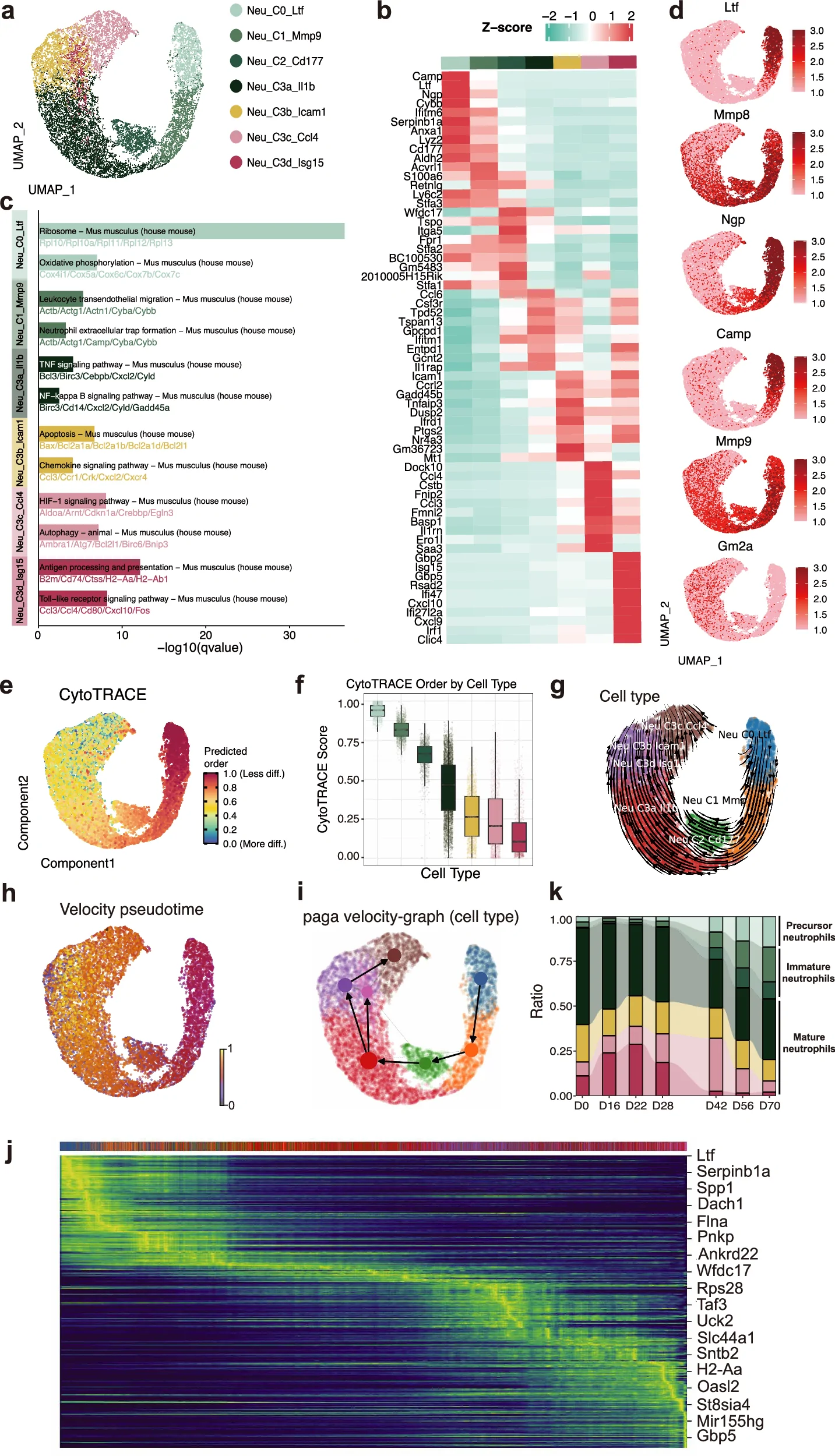
Immunological features of neutrophil subsets during *S. japonicum* infection. **a** UMAP clustering of neutrophil subtypes (total cells: 16,440). Each point represents a single cell, and each color indicates a neutrophil subset. **b** Heatmap of marker gene expression in the neutrophil subsets. **c** GO biological process terms enriched in genes upregulated in specific neutrophil subsets. Representative terms are shown. **d** UMAP feature plots showing the expression of representative granule-associated genes in the neutrophil subsets. **e** CytoTRACE analysis of differentiation potential across the neutrophil subsets. **f** CytoTRACE cell developmental trajectory, grouped by cell type. CytoTRACE analysis was performed using integrated single-cell RNA-sequencing data from mouse liver samples collected at seven time points, with 2 biologically independent mice per time point (14 samples in total). Box plots show the median (centre line), the 25th and 75th percentiles (box limits), and the minimum and maximum values (whiskers). RNA velocity analysis: **g** directional cell trajectory and **h** pseudotime distribution across neutrophil subsets. **i** Partition-based Graph Abstraction coarse-grained trajectory visualization of neutrophil subsets. **j** Expression levels of the top 300 genes (ranked by likelihood score along the differentiation trajectory) across neutrophil subsets. **k** Temporal dynamics of neutrophil subset proportions during infection.

The granulocyte cluster primarily consists of neutrophils in this study. Recent studies have highlighted marked variability in neutrophil function^25–27^. However, in helminth diseases, neutrophil heterogeneity remains underexplored. To elucidate neutrophil heterogeneity, we analyzed 16,440 granulocytes and identified seven transcriptionally distinct neutrophil subsets (Fig. 2a), each with a unique molecular profile (Fig. 2b, c, and Supplementary Data 1). Neu_C0_Ltf was characterized by high lactoferrin (*Ltf*) and antimicrobial peptide (*Camp*) expression and was enriched in ribosomal and oxidative phosphorylation pathways, suggesting a hypermetabolic state conducive to rapid biosynthetic activity. Neu_C1_Mmp9 was dominated by matrix metalloproteinases (*MMP8/9*) and was associated with leukocyte transendothelial migration and neutrophil extracellular trap (NET) formation, suggesting roles in tissue remodeling and egg entrapment. As a CD177-expressing neutrophil subset, Neu_C2_Cd177 may have a high capacity for vascular adhesion and transendothelial migration, thereby potentially initiating the recruitment of other inflammatory cells to egg deposition sites. The heterogeneous Neu_C3 population comprised four subsets with distinct functional implications: Neu_C3a_Il1b was enriched in the TNF and NF-κB signaling pathways, and might drive pro-inflammatory responses; Neu_C3b_Icam1 was associated with chemokine signaling, and could regulate immune cell recruitment; Neu_C3c_Ccl4 showed activation of the HIF-1 signaling and autophagy pathways, and may represent adaptation to the hypoxic environment of granulomas; and Neu_C3d_Isg15 was enriched in antigen processing and presentation and Toll-like receptor signaling, potentially bridging the innate and adaptive immune systems.

A well-established mechanism underlying neutrophil granule heterogeneity is the temporal regulation of biosynthesis during cellular development, wherein proteins that are synthesized concurrently are packaged into the same granules without the need for type-specific sorting^28,29^. Myeloperoxidase (MPO), defensins, and elastase are hallmark components of azurophil (primary) granules. In contrast, LTF-positive granules are classified as specific (secondary) granules. Finally, granules that are negative for LTF but positive for gelatinase are defined as gelatinase (tertiary) granules. Analysis of granule-related gene expression across neutrophil differentiation stages (Fig. 2d) showed that *Ltf* and *Camp* were highly expressed in the Neu_C0_Ltf subcluster, while *Mmp8* and *Mmp9* were markedly upregulated in the Neu_C1_Mmp9 subcluster. These results indicate that Neu_C0_Ltf cells were the primary source of secondary granules, while Neu_C1_Mmp9 cells were mainly responsible for tertiary granule production. Notably, no specific neutrophil subpopulation was significantly associated with primary granules.

To characterize neutrophil development, we first defined the maturation stages of the neutrophil subclusters based on published data^30^: Neu_C0_Ltf represents proliferation-competent precursor neutrophils (preNeus); Neu_C1_Mmp9 and Neu_C2_Cd177 correspond to non-proliferating immature neutrophils; and Neu_C3a_Il1b, Neu_C3b_Icam1, Neu_C3c_Ccl4, and Neu_C3d_Isg15 constitute mature neutrophils. CytoTRACE analysis, which quantifies differentiation potential, showed continuous development among these subsets: CytoTRACE scores decreased from Neu_C0_Ltf (highest potential) through Neu_C1_Mmp9 and Neu_C2_Cd177 to Neu_C3a_Il1b, whereas Neu_C3b_Icam1, Neu_C3c_Ccl4, and Neu_C3d_Isg15 showed discrete distribution patterns (Fig. 2e, f). This trajectory aligned with both cellular maturation status and maturation score (Supplementary Fig. 3a and Supplementary Data 2). RNA velocity analysis further validated this developmental path, as the resulting directional cell trajectories and pseudotime distributions (Fig. 2g, h) supported progression from preNeus to immature and mature neutrophils. Additionally, partition-based graph abstraction (PAGA) trajectory analysis identified two distinct terminal fates of mature neutrophils, with Neu_C3c_Ccl4 and Neu_C3d_Isg15 representing independent maturation endpoints (Fig. 2i). To explore transcriptional dynamics along this trajectory, we profiled the expression patterns of the top 300 genes ranked by likelihood score (along the differentiation trajectory) across subsets (Fig. 2j). The velocity and expression of representative early, intermediate, and late drivers in individual neutrophils are shown in Supplementary Fig. 3b.

The proportional dynamics of the neutrophil subsets appeared to reflect phase-specific immune adaptations to *S. japonicum* infection (Fig. 2k). In the pre-egg phase, mature neutrophils dominated the hepatic neutrophil compartment. In contrast, the acute and chronic phases were characterized by dramatic expansion of the precursor (Neu_C0_Ltf) and immature (Neu_C1_Mmp9, Neu_C2_Cd177) neutrophil subsets, together with a progressive decline in mature subsets, particularly Neu_C3d_Isg15. Notably, the Neu_C2_Cd177 subset was restricted to the acute and chronic phases, which coincided with egg deposition and granuloma formation. This temporal association raises the hypothesis that egg deposition generates an inflammatory milieu triggered by soluble egg antigens (SEA). The microenvironment may promote the recruitment and local expansion of precursor and immature neutrophils to hepatic granulomas, where these subsets may help modulate egg-induced pathology (e.g., via ROS production, NET formation, or proteolytic enzyme release).

### Dynamic macrophage polarization shift throughout *S. japonicum*

**Fig. 3:**
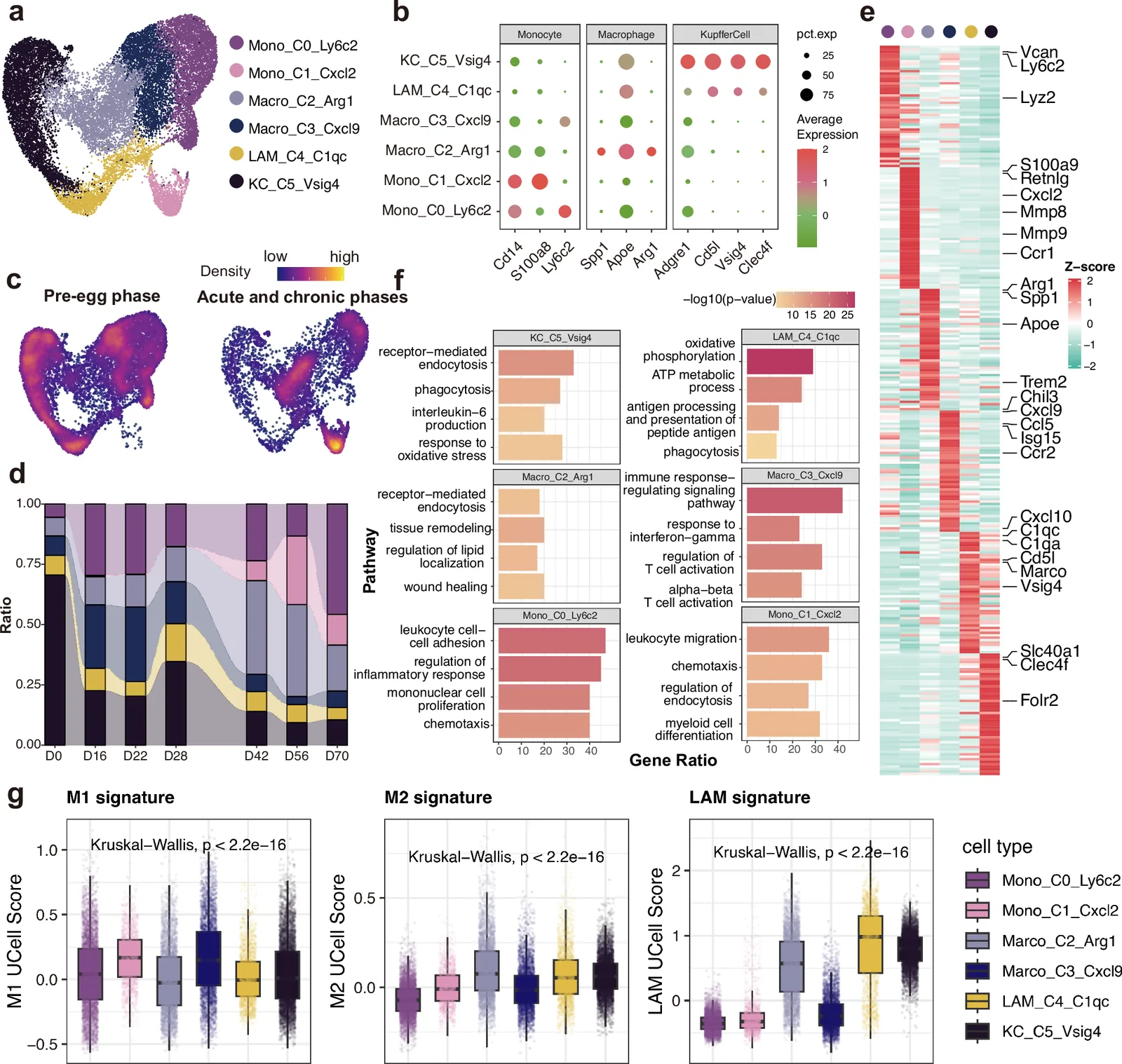
Immunological features of mononuclear phagocytes during *S. japonicum* infection. **a** UMAP visualization of six mononuclear phagocyte clusters (including monocytes, macrophages, liver-associated macrophages [LAMs], and Kupffer cells). **b** Bubble plots showing marker gene expression in each mononuclear phagocyte cluster. **c** Cell density distribution of mononuclear phagocyte clusters in the pre-egg phase (left) and the acute/chronic phases (right). **d** Temporal dynamics of mononuclear phagocyte subset proportions throughout infection (D0–D70). **e** Heatmap of marker gene expression in mononuclear phagocyte subsets. **f** Enriched biological process terms (GO) for cluster-specific upregulated genes in the mononuclear phagocyte subsets. Representative terms are shown. **g** UCell scores for the M1, M2, and LAM functional signatures across the mononuclear phagocyte clusters. UCell scores were calculated from integrated single-cell RNA-sequencing data of mouse liver samples collected across seven time points, with 2 biologically independent mice per time point. Box plots show the median (centre line), the 25th and 75th percentiles (box limits), and the minimum and maximum values (whiskers). Differences among cell subsets were assessed using the Kruskal–Wallis test.

We next characterized the heterogeneity of mononuclear phagocytes. scRNA-seq analysis identified 14,367 mononuclear phagocytes, which were classified into six distinct clusters: two monocyte subsets (Mono_C0_Ly6c2 and Mono_C1_Cxcl2), two macrophage subsets (Macro_C2_Arg1 and Macro_C3_Cxcl9), a lipid-associated macrophage (LAM) cluster (LAM_C4_C1qc) and a Kupffer cell (KC) cluster (KC_C5_Vsig4) (Fig. 3a). Differential gene expression heatmaps showed cluster-specific transcriptional profiles (Fig. 3b, e and Supplementary Data 3). Functional scoring using established gene signatures, as shown in Fig. 3g and Supplementary Data 2, indicated that the Macro_C3_Cxcl9 cluster exhibited the highest M1-polarization score, the Macro_C2_Arg1 cluster showed predominantly M2 polarization, and the LAM_C4_C1qc cluster displayed the strongest enrichment for the LAM signature.

Significant compositional changes were observed in mononuclear phagocyte populations between the pre-egg and post-egg phases (Fig. 3c, d). The Mono_C1_Cxcl2 subset, which was virtually absent before egg deposition, expanded into a specific, detectable population during acute and chronic infection. This subset showed significant enrichment for pathways related to leukocyte migration, chemotaxis, regulation of endocytosis, and myeloid cell differentiation, indicating a potential role in targeted cell infiltration into granulomas, SEA internalization and processing, and potential differentiation into macrophages (Fig. 3f).

A dynamic shift was observed in the ratio of M1-like (Macro_C3_Cxcl9) and M2-like (Macro_C2_Arg1) macrophages throughout infection (Fig. 3c, d). During the pre-egg phase (16 to 22 d.p.i.), the Macro_C3_Cxcl9 (M1-like) macrophage cluster predominated, accounting for 2.0-fold the number of Macro_C2_Arg1 (M2-like) macrophages, which indicates a Th1-skewed pro-inflammatory response, likely driven by schistosome worm antigens. By 28 d.p.i., coinciding with initial egg deposition, the M1:M2 ratio equilibrated at approximately 1.2:1. In the acute post-egg phase (42 to 56 d.p.i.), a marked Th2 shift occurred, with the Macro_C2_Arg1 (M2-like) macrophage cluster surpassing Macro_C3_Cxcl9 (M1-like) macrophages by 7.4-fold. This shift was likely triggered by SEA, which induces M2 polarization and promotes granuloma formation and tissue repair via Arg1-mediated pathways. In the chronic phase (70 d.p.i.), the M2:M1 ratio decreased to approximately 2.8:1, suggesting partial resolution of Th2-driven fibrosis. Consistent with this, the Macro_C2_Arg1 subset exhibited high expression of CD9, TREM2, and SPP1 (Supplementary Fig. 4), a molecular signature characteristic of pro-fibrotic macrophages in human cirrhotic liver and mouse CCl₄-induced fibrosis models, suggesting a conserved pro-fibrotic program across different etiologies of liver fibrosis^31^.

### T/NK cell, B cell, DC and endothelial cell dynamics during schistosome egg–induced granuloma formation

**Fig. 4:**
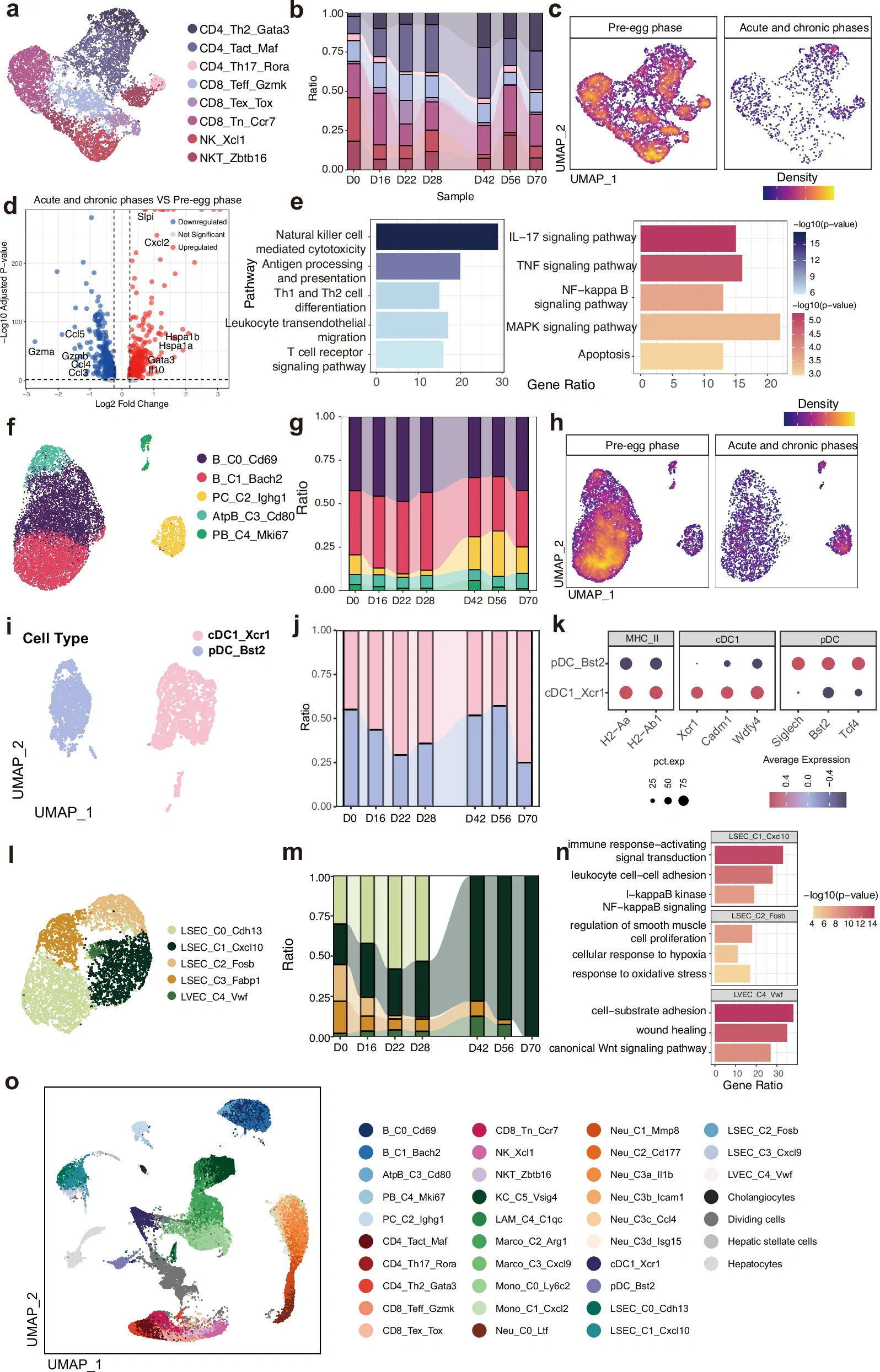
Distribution of T/NK cells, B cells, dendritic cells, and endothelial cells during experimental *S. japonicum* infection. **a** UMAP visualization of T/NK cell subsets. Each dot represents a single cell, color-coded by the cluster it belongs to. **b** Dynamic proportions of the T/NK subsets throughout infection (D0–D70). **c** Cell density distribution of T/NK cells in the pre-egg phase (left) and the acute/chronic phases (right). **d** Volcano plots of genes that were differentially expressed between the pre-egg phase and the acute/chronic phase in T/NK cells. Differentially expressed genes were identified using the Wilcoxon rank-sum test implemented in Seurat, with Benjamini–Hochberg correction for multiple testing. Genes with adjusted *P* values below the predefined threshold were considered significant. **e** Enriched biological process pathways (GO) for genes that were differentially expressed in T/NK cells. Left, pre-egg phase–dominant (downregulated in acute/chronic phases) pathways. Right, acute/chronic phase–dominant (upregulated) pathways. Representative terms are shown. **f** UMAP visualization of B cell subsets, color-coded by clusters. **g** Temporal dynamics of B cell subset proportions throughout infection (D0–D70). **h** Cell density distribution of B cells in the pre-egg phase (left) and the acute/chronic phases (right). **i** UMAP visualization of DC subsets, including cDC1 (Xcr1^+^) and pDC (Bst2^+^). **j** Temporal changes in DC subset composition across infection (D0–D70). **k** Dot plot showing expression of canonical marker genes and antigen presentation molecules across DC subsets. **l** UMAP visualization of endothelial cell subsets, color-coded by cluster. **m** Temporal dynamics of endothelial cell subset proportions throughout infection (D0–D70). **n** Enriched functional pathways in the LESC_C1_Cxcl10, LESC_C2_Fosb, and LVEC_C4_Vwf subsets, which were retained post–egg deposition. Functional enrichment analysis was performed using clusterProfiler, and adjusted *P* values were calculated using the Benjamini-Hochberg method. **o** Integrated UMAP visualization of all merged hepatic cell subsets, showing the overall cellular distribution and organizational landscape.

Subcluster analysis of 9615 T cells and NK cells revealed eight distinct subsets, which had unique molecular signatures and functional roles: three CD4^+^ T cell subpopulations, three CD8^+^ T cell subpopulations, one NK cell subset, and one natural killer T (NKT) cell subset (Fig. 4a and Supplementary Fig. 5a). Among the CD4^+^ T cell clusters, the CD4_Th2_Gata3 subset was defined by high *Gata3* and *IL4* expression, which is consistent with a classical Th2 phenotype. The CD4_Th17_Rora subset was characterized by elevated *Rora* and *Il23r* expression and was identified as Th17 cells. The CD4_Tact_Maf subset included a diverse population of activated T cells, including Th1-like cells, that co-expressed *Rora*. Among the CD8^+^ T cell subsets, the CD8_Teff_Gzmk subset showed high *Gzmk* expression, which is indicative of pro-inflammatory effects^32^. The CD8_Tex_Tox subset was marked by *Tox* and *Pdcd1* expression and represented exhausted CD8^+^ T cells. The CD8_Tn_Ccr7 subset was classified as naïve T cells and distinguished by elevated *Ccr7* and *Sell* expression levels. The cellular composition analysis showed a significant increase in the CD4_Th2_Gata3 (Th2 cell) subset following egg production (Fig. 4b). This is consistent with the transition from worm antigen–driven Th1 responses to egg antigen–driven Th2 responses^33,34^.

Given the limited number of T cells in the acute and chronic phases, we grouped the data from 16, 22, and 28 d.p.i. into the pre-egg phase group and the data from 42, 56, and 70 d.p.i. into the post-egg phase group (Fig. 4c). Volcano plot analysis further delineated phase-specific T cell gene expression (Fig. 4d): genes that were upregulated in the post-egg phase (e.g., *Il10*, *Slpi, Hspa1b, Gata3*) were enriched in anti-inflammatory pathways, while genes enriched in the pre-egg phase (e.g., *Gzma*, *Gzmb*) were associated with cytotoxic effector functions. Corresponding pathway enrichment analysis showed that dominant pathways in the pre-egg phase included antigen processing and presentation, T cell differentiation, and T cell receptor (TCR) signaling pathways, indicating antigen recognition and cytotoxic T cell activation functions presumably associated with targeting schistosome worm antigens (Fig. 4e, left panel). The significant enrichment of the IL-17 signaling pathway that we observed after egg deposition is consistent with established mechanisms, in which IL-17A, secreted by Vγ2 γδ T cells and Th17 cells, serves as a critical mediator of neutrophil recruitment in murine schistosomiasis (Fig. 4e, right panel)^35,36^.

A total of 14,050 B cells were identified and classified into five distinct clusters based on their expression profiles (Fig. 4f, g and Supplementary Fig. 5b). While the overall proportion of B cells decreased significantly post–egg production (Fig. 4h), which similar to the trend that we observed in T cells, the PC_C2_Ighg1 cluster (representing plasma cells) exhibited marked expansion (Fig. 4g). This suggests that antibody production may play a protective role in schistosome egg granuloma formation, with humoral immunity contributing to mitigation of egg-induced tissue damage.

We identified two subclusters of DCs according to the classic DC cell markers of mice (Fig. 4i). The conventional Type 1 DC subset (cDC1, cDC1_Xcr1) was found to be characterized by elevated expression of Xcr1 and *Cadm1* (Fig. 4k). This subset is specialized in the cross-presentation of exogenous antigens to prime CD8^+^ T cells. It was observed that this population is enriched during the early stages of *S. japonicum* infection, reflecting their critical role in initiating the early cytotoxic immune response (Fig. 4j). Plasmacytoid DC (pDC, pDC_Bst2) was defined by the expression of *Bst2*, *Siglech* and *Tcf4* (Fig. 4k). The proportion of pDC peaks during the late phase, suggesting that this subset is associated with a chronic inflammatory response as previously reported in *S. mansoni* infection^37^.

Analysis of 7806 endothelial cells identified five subsets through vascular categorization, primarily consisting of capillary endothelial cells (LESC_C0_Cdh13, LESC_C1_Cxcl10, LESC_C2_Fosb, LESC_C3_Fabp1) and capillary venule endothelial cells (LVEC_C4_Vwf) (Fig. 4l and Supplementary Fig. 5c, d, and Supplementary Data 4). Temporal heterogeneity was observed: only three subsets (LESC_C1_Cxcl10, LESC_C3_Fabp1, and LVEC_C4_Vwf) were present in the acute phase and only the LESC_C1_Cxcl10 subset persisted into the chronic phase (Fig. 4m). The LESC_C1_Cxcl10 subset was significantly enriched for pathways associated with immune response–activating signal transduction and leukocyte cell–cell adhesion, indicating a critical role in orchestrating immune cell recruitment and granuloma formation following egg deposition (Fig. 4n).

The refined classification of subclusters is presented in Fig.4o. In addition, the list of marker genes for 37 identified sub-populations is provided in Supplementary Data 5. This comprehensive dataset provides a valuable resource for further characterizing the molecular signatures of these subclusters.

### Stage-specific remodeling of intercellular communication and amplified CXCL2-CXCR2 crosstalk in granulocyte-associated cell pairs

**Fig. 5:**
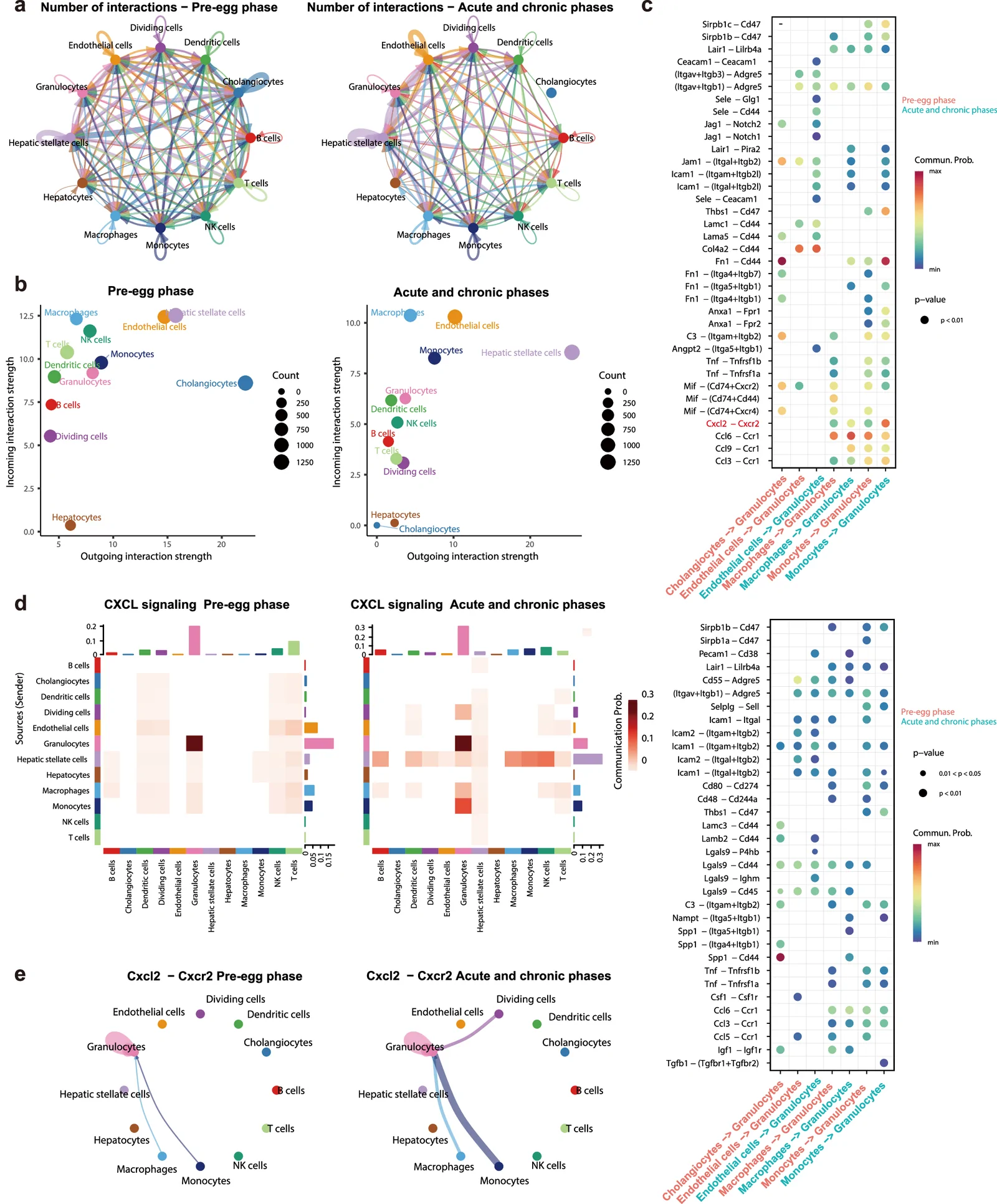
Stage-specific remodeling of intercellular communications and CXCL2-CXCR2 signaling in liver NPCs. **a** Chord diagrams illustrating ligand–receptor (L-R) interaction networks between distinct cell clusters in the pre-egg phase (left) and the acute/chronic phases (right). Nodes represent cell types, and edges denote L-R–mediated intercellular crosstalk. **b** Quantification of intercellular communication features (pre-egg phase, left; acute/chronic phases, right). Scatter plots show the outgoing interaction strength (x-axis) and the incoming interaction strength (y-axis) of each cell cluster, with the size of the point indicating the total interaction count of the cluster. **c** Differential L-R pair interactions associated with granulocytes (neutrophils). The upper panel displays L-R pairs with upregulated interactions in the acute/chronic phase relative to the pre-egg phase, while the lower panel shows downregulated L-R pairs. “Common Prob.” denotes the communication probability of each L-R pair. Differential ligand–receptor interactions were identified using CellChat by comparing communication probabilities between groups. Statistical significance was assessed using the permutation framework implemented in CellChat. **d** Heatmaps of CXCL family signaling activity between cell clusters in the pre-egg phase (left) and the acute/chronic phases (right). The color intensity indicates the communication strength of CXCL-related L-R pairs between the sender (y-axis, ligand-expressing cell cluster) and the receiver (x-axis, receptor-expressing cell cluster). CXCL signaling activities were inferred using CellChat based on communication probabilities calculated from single-cell transcriptomic data. **e** Cell-specific CXCL2-CXCR2 axis communication networks in the pre-egg phase (left) and the acute/chronic phases (right). Edges represent the direction and strength of CXCL2-CXCR2 crosstalk between cell types.

To investigate intercellular communication dynamics throughout infection, we used the CellChat algorithm to analyze signaling pathways between distinct cell types in the pre-egg phase versus the acute/chronic phases. A chord diagram was generated to summarize the ligand–receptor (L-R) interactions between cell population pairs (Fig. 5a). Following egg deposition, the total number of cellular interactions decreased; however, specific crosstalk was markedly enhanced. This enhanced crosstalk included interactions between HSCs and myeloid cells (e.g., granulocytes, monocytes, and macrophages), as well as interactions between HSCs and endothelial cells. Analysis of interaction strength (Fig. 5b) further showed phase-specific functional shifts: in the acute/chronic phases, HSCs showed increased outgoing interaction strength but reduced incoming strength, which reflects a transition toward a more autonomous, pro-active regulatory state. Conversely, T and NK cells exhibited decreased incoming interaction strength following egg deposition, which indicates an impaired capacity to receive polarizing or activating signals. The interaction strength of cholangiocytes (biliary epithelial cells) was negligible in the acute/chronic phases, as these cells were not detected during these stages.

To further dissect phase-specific changes in L-R crosstalk involving granulocytes, i.e. neutrophils, we focused on four critical communication pairs: monocyte–granulocyte, macrophage–granulocyte, endothelial cell–granulocyte, and cholangiocyte–granulocyte (Fig. 5c). Notably, the CXCL2-CXCR2 L-R interaction intensity was significantly elevated in the monocyte–granulocyte and macrophage–granulocyte communication pairs during the acute/chronic phases (Fig. 5c, upper panel). This finding indicates that CXCL2-CXCR2 paracrine signaling between monocytes/macrophages and neutrophils is specifically amplified following egg deposition, potentially serving as a key regulatory axis driving neutrophil recruitment.

Next, we extended our analysis to CXCL family signaling across all cell populations to identify the key functional axes within this family. This analysis confirmed that granulocytes exhibited significantly enhanced CXCL-dependent crosstalk with monocytes, macrophages, dividing cells, and granulocytes themselves (Fig. 5d). As expected, among all CXCL-dependent crosstalk pathways, the CXCL2-CXCR2 axis was the most potent driver (Fig. 5e).

### The CXCL2-CXCR2 axis orchestrates neutrophil recruitment via autocrine and paracrine mechanisms in *S. japonicum* egg–induced hepatic granulomas

**Fig. 6:**
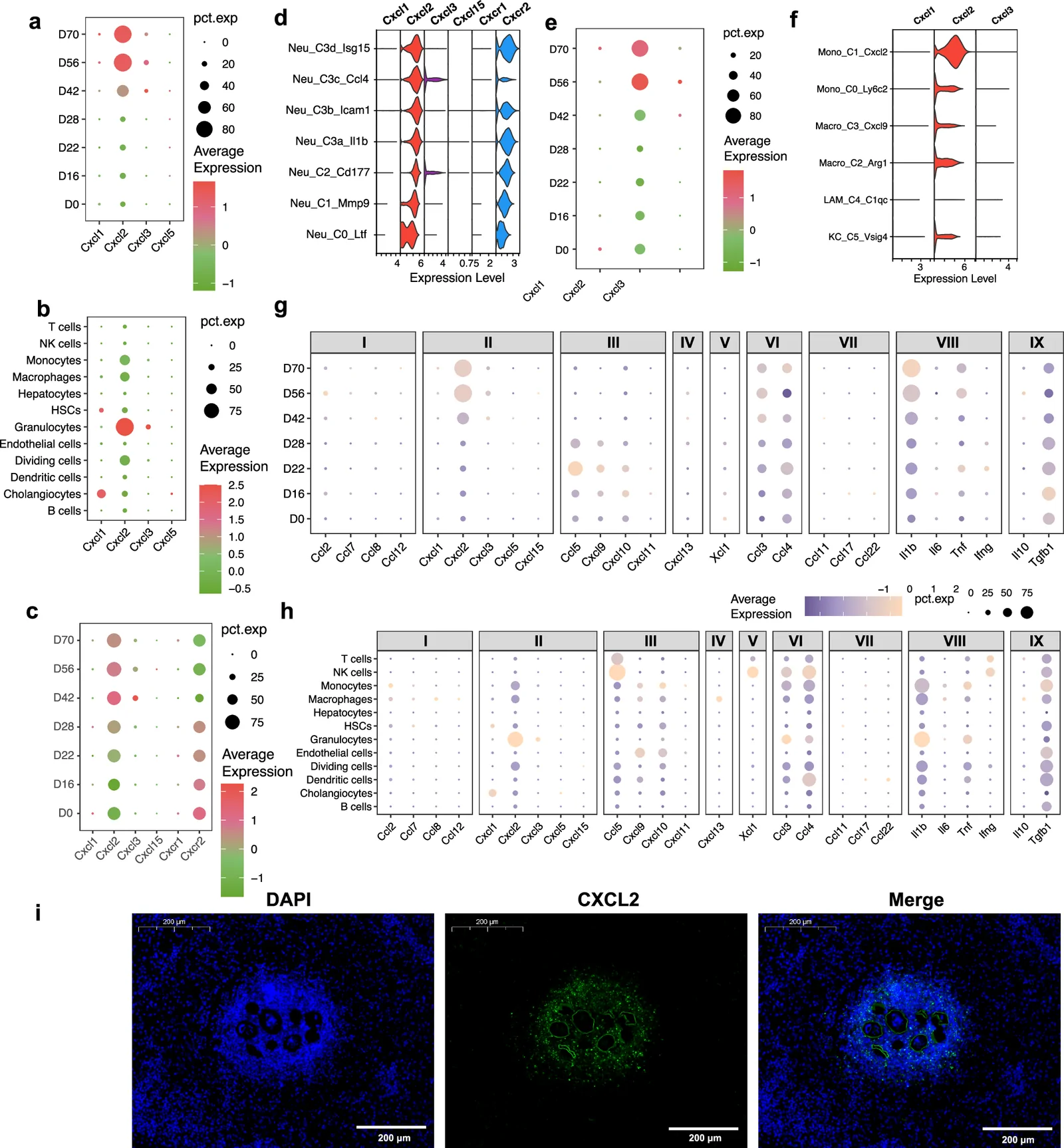
Temporal expression and cell-specific profiles of key chemokines and cytokines during schistosome infection. **a** Temporal CXCL1, CXCL2, CXCL3, and CXCL5 expression across all NPCs during schistosome infection. **b** CXCL1, CXCL2, CXCL3, and CXCL5 expression within distinct NPC clusters. **c** Temporal expression of CXCL1, CXCL2, CXCL3, CXCL5 and their receptors CXCR1 and CXCR2 in neutrophils during infection. **d** CXCL1, CXCL2, CXCL3, CXCL5, CXCR1, and CXCR2 expression across neutrophil subsets. **e** Temporal CXCL1, CXCL2, and CXCL3 expression in mononuclear phagocytes during infection. **f** CXCL1, CXCL2, and CXCL3 expression within mononuclear phagocyte subsets. **g** Expression dynamics of key chemokines and cytokines over the infection course. Panels 1–7 display chemokines related to the recruitment of seven immune cell subsets. I: monocyte-macrophages, II: neutrophils, III: T cells, IV: B cells, V: DCs, VI: NK cells, VII: eosinophils. Panels 8–9 represent pro- and anti-inflammatory cytokines. VIII: pro-inflammatory cytokines, IX: anti-inflammatory cytokines. **h** Expression dynamics of key chemokines and cytokines across distinct cell clusters. Panels 1–9 follow the same grouping classification as in (**g**). **i** Immunofluorescence staining for CXCL2 in hepatic tissue sections at 42 days post–*S. japonicum* infection. From left to right: DAPI staining; CXCL2-specific staining; merged image (scale bar = 200 μm). Multiplex immunofluorescence and immunofluorescence staining experiments shown in Fig. 6i were performed using samples from four biologically independent mice, and representative images are shown. Similar results were obtained across all samples analyzed.

The findings described above indicate that CXCL2 is the predominant chemokine mediating neutrophil chemotaxis within egg-induced granulomas. The CXCL2 expression levels in liver NPCs show a progressive upward trend after infection, with a significant increase observed from 42 d.p.i. (Fig. 6a). This temporal pattern is highly consistent with our single-cell sequencing data, which showed a significant expansion in neutrophils at the same stage of infection.

To identify cellular sources of CXCL2, we first analyzed its expression across different immune populations. Granulocytes (neutrophils) were identified as the primary producers, followed by monocytes and macrophages (Fig. 6b). Temporal analysis of neutrophils revealed that CXCL2 expression increased progressively throughout the course of infection, whereas expression of its receptor, CXCR2, exhibited a declining trend (Fig. 6c). Further dissection of neutrophil subsets demonstrated that CXCL2 was widely expressed across all subtypes, while CXCR2 was expressed in all neutrophil subsets except for Neu_C3c_Ccl4 (Fig. 6d). Similarly, within mononuclear phagocytes, CXCL2 expression was temporally upregulated (Fig. 6e), and among mononuclear phagocyte subsets, the Mono_C1_Cxcl2 cluster contributed most significantly to CXCL2 production. Notably, the Mono_C1_Cxcl2 cluster emerged specifically following egg deposition, which suggests that this subset plays a critical role in initiating neutrophil recruitment (Fig. 6f).

Next, we profiled the expression dynamics of other key immune factors (Fig. 6g, h). The pro-inflammatory cytokines IL-1β and TNF, along with the NK cell chemokine CCL3, demonstrated sustained upregulation throughout disease progression. These factors originated from diverse cellular sources: IL-1β was primarily produced by neutrophils, TNF by macrophages, and CCL3 by monocytes. In contrast, T cell–recruiting chemokines, including CCL5, CXCL9, and CXCL10, exhibited a distinct biphasic expression pattern, with levels peaking at 22 d.p.i. This temporal profile aligns with the expansion of T cells that we observed during the pre-egg stage of infection. Notably, transcripts corresponding to eosinophil-specific chemokines were not detected at any infection stage.

To further verify the role of the CXCL2-CXCR2 axis in granuloma formation, we analyzed the distribution of CXCL2 expression in the mouse liver at 42 d.p.i. Immunofluorescence staining results showed that CXCL2 expression was predominantly concentrated within hepatic granulomas (Fig. 6i and Supplementary Fig. 6a, b), supporting the potential involvement of the CXCL2-CXCR2 axis in granuloma development.

### The histologic morphology of granulomas is consistent with cell composition changes during *S. japonicum* infection

**Fig. 7:**
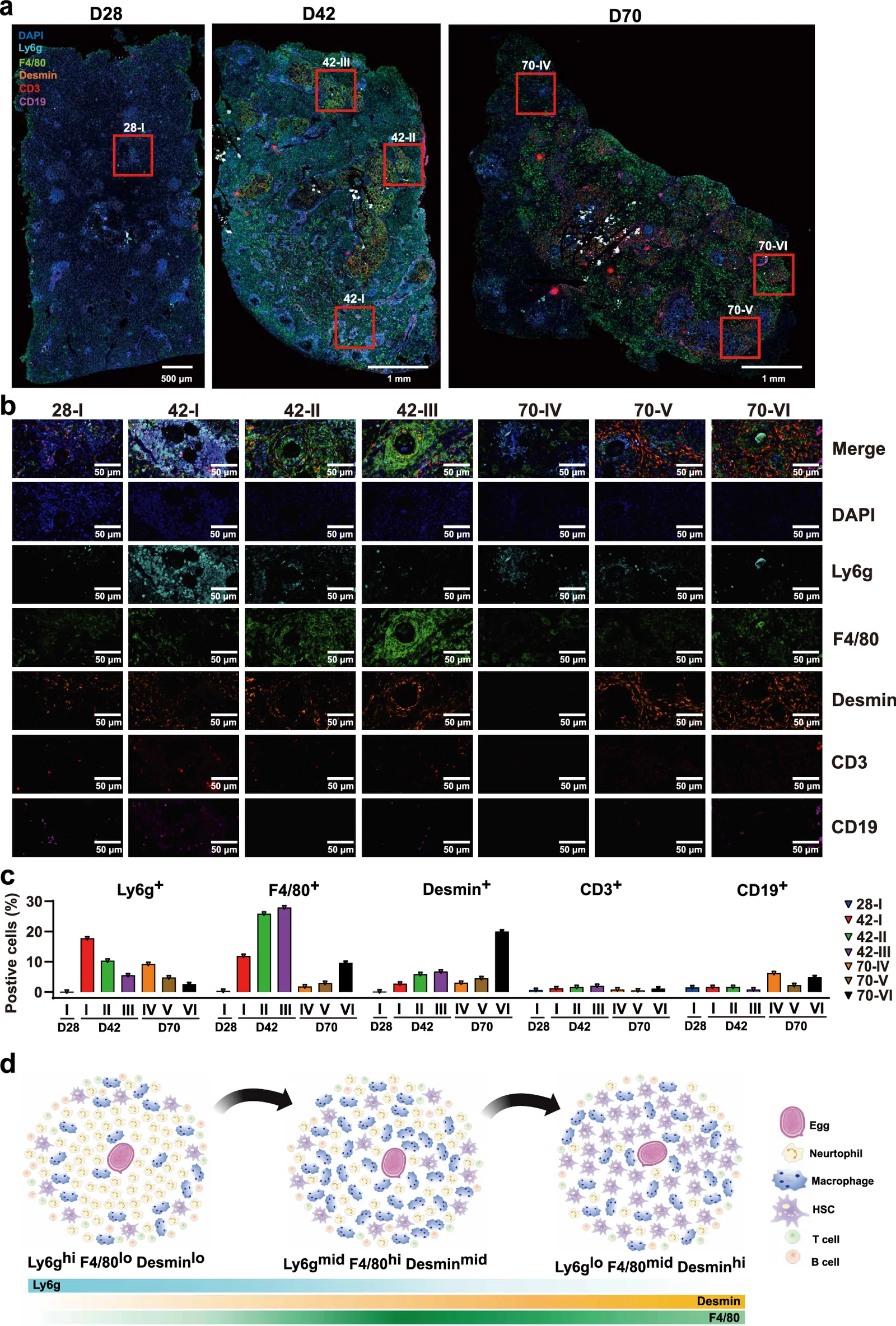
Hepatic egg granuloma heterogeneity and development in *S. japonicum*–infected mice. **a** Whole-slide multiplex immunofluorescence staining of mouse liver tissue at 28, 42, and 70 d.p.i. Red boxes denote representative granuloma types at each stage (e.g., 28-I at 28 d.p.i., 42-I/II/III at 42 d.p.i., 70-IV/V/VI at 70 d.p.i.). Fluorescent labels: DAPI (nuclear staining, dark blue), Ly6G (neutrophils, light blue), F4/80 (macrophages, green), Desmin (HSCs, orange), CD3 (T cells, red), CD19 (B cells, purple). Scale bars: 500 μm (28 d.p.i.), 1 mm (42/70 d.p.i.). **b** Layered immunofluorescent visualization of cellular composition across granuloma types. All representative images were obtained from single- or low-egg-count granulomas wherever feasible to preserve the intact morphological architecture of individual lesions. For confluent types 42-III and 70-V, representative individual granulomas were selected to reflect the overall cellular distribution patterns of these subtypes. Scale bars: 50 μm. **c** The proportions of Ly6G^+^, F4/80^+^, Desmin^+^, CD3^+^, and CD19^+^ cells in different types of granulomas. **d** A schematic diagram of the development of hepatic egg granulomas. The color gradients at the bottom reflect the dynamic changes in Ly6G, Desmin, and F4/80 abundance. For quantification, each granuloma was considered an independent unit of analysis. The numbers of granulomas analyzed were *n* = 20 (28-I), *n* = 25 (42-I), *n* = 4 (42-II), *n* = 2 (42-III), *n* = 7 (70-IV), *n* = 2 (70-V), and *n* = 16 (70-VI).

We next investigated whether the histologic features of hepatic granulomas are consistent with the dynamic changes in cell composition that we observed during *S. japonicum* infection. TissueFAXS cytometry was used to detect the distribution of the major cell populations, i.e., Ly6G^+^ cells (neutrophils), F4/80^+^ cells (macrophages), Desmin^+^ cells (HSCs), CD3^+^ cells (T cells), and CD19^+^ cells (B cells), within and around granulomas (Fig. 7a and Supplementary Fig. 7c).

We observed significant heterogeneity among hepatic granulomas both across different infection stages and within the same infection stage (Supplementary Table 1). At 28 d.p.i., 20 atypically diffuse granulomas comprising an average of two eggs were observed in the liver, which were designated as type 28-I, with an area ranging from 0.01 to 0.12 mm^2^. At 42 d.p.i., typical granulomas were evident. A total of 31 granulomas were classified into three types: type 42-I (*n* = 25, area: 0.03–0.43 mm^2^, average 4.4 eggs), type 42-II (*n* = 4, area: 0.74–1.23 mm^2^, average 23 eggs), and type 42-III (*n* = 2, area: 4.23–6.48 mm^2^, average 179 eggs; characterized by massive egg deposition and confluence). At 70 d.p.i., 25 granulomas were classified into three types: type 70-IV (*n* = 7, area: 0.08–0.4 mm^2^, average 5.57 eggs), type 70-V (*n* = 2, area: 1.51–2.03 mm^2^, average 81.50 eggs), and type 70-VI (*n* = 16, area: 0.10–0.34 mm^2^, average 2.94 eggs).

Among type 42-I, II, and III granulomas, the proportion of Ly6G^+^ neutrophils gradually decreased (from 17.8% to 5.5%), whereas the proportion of F4/80^+^ macrophages (from 11.9% to 28.0%) and Desmin^+^ HSCs (from 2.8% to 6.8%) progressively increased (Fig. 7b, c). Among type 70-IV, V, and VI granulomas, type 70- IV granulomas exhibited a cellular composition similar to type 42-I, with a high proportion of Ly6G^+^ neutrophils (9.2%) but low abundances of F4/80^+^ macrophages (1.8%) and Desmin^+^ HSCs (3.0%), indicating these granulomas are induced by newly deposited eggs. Type 70-V granulomas probably develop from type 42-III, as both are characterized by large lesion size and high egg burden. But the most striking difference is that the macrophage proportion drops sharply to 3.0% in type 70-V, compared with 28.0% in type 42-III. Type 70-VI is a representative advanced granuloma, characterized by the highest Desmin^+^ HSC percentage (20%) across all types, with neutrophils and macrophages comprising just 2.9% and 9.6%, respectively.

Based on the differences in the proportions of Ly6G^+^, F4/80^+^, and Desmin^+^ cells, we delineated a three-stage evolutionary trajectory for schistosome-induced hepatic granulomas: early granulomas (Ly6G^hi^F4/80^lo^Desmin^lo^, type 42-I); developing granulomas (Ly6G^mid^F4/80^hi^Desmin^mid^, type 42-II); and advanced granulomas (Ly6G^lo^F4/80^mid^Desmin^hi^, type 70-VI) (Fig. 7d). Type 28-I was excluded as it represents atypical lesions from immature eggs, while type 70-IV is similar to early-stage type 42-I. Types 42-III and 70-V are confluent lesions unsuitable for precise cellular quantification.

Regarding spatial distribution, neutrophils were mostly concentrated within 25 μm of the granuloma centers, and macrophages and HSCs were evenly dispersed throughout the granulomas, while T cells and B cells were mainly distributed ~150 μm from the egg centers (Supplementary Fig. 7a, b).

### Neutrophil subsets within granulomas display distinct spatial zones

**Fig. 8:**
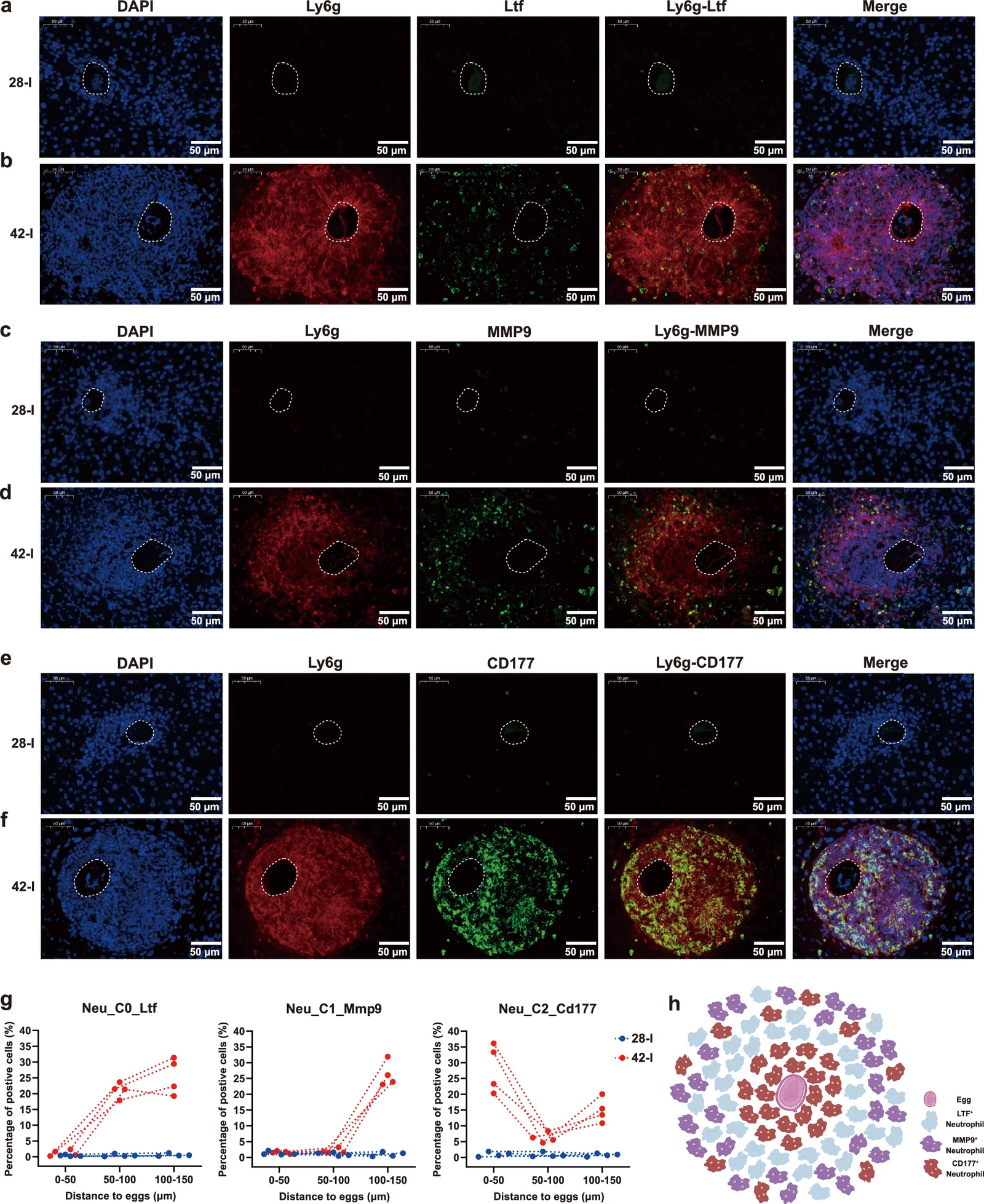
Spatial distribution of immature neutrophil subsets within *S. japonicum* egg–induced granulomas. Immunofluorescence staining of Ly6G^+^/LTF^+^ cells in 28-I (**a**) and 42-I (**b**) granulomas. Immunofluorescence staining of Ly6G^+^/MMP9^+^ cells in 28-I (**c**) and 42-I (**d**) granulomas. Immunofluorescence staining of Ly6G^+^/CD177^+^ cells in 28-I (**e**) and 42-I (**f**) granulomas. **g** Proportions of neutrophil subsets at different distances from the egg (*n* = 4 per group). **h** Schematic illustration of the spatial zonation of neutrophil subsets in 42-I granulomas. The dashed lines outline the eggs. Scale bars: 50 μm. Multiplex immunofluorescence and immunofluorescence staining experiments shown in the Figure were performed using samples from four biologically independent mice, and representative images are shown. Similar results were obtained across all samples analyzed.

A distinct feature of Ly6G^hi^F4/80^lo^Desmin^lo^ granulomas is a high proportion of neutrophils. In addition, the scRNA-seq results revealed a significant increase in the proportions of three neutrophil subsets (Neu_C0_Ltf, Neu_C1_Mmp9, and Neu_C2_Cd177) in the liver following granuloma formation. To investigate whether these three neutrophil subsets exhibit different spatial distributions within granulomas, we analyzed their relative spatial distribution by immunofluorescence in two granuloma types: 28-I and 42-I. These types are characterized by low egg counts and minimal intra-type heterogeneity. Consistent with the scRNA-seq data, neutrophil infiltration was negligible in 28-I granulomas (Fig. 8a, c, e, g). For the 42-I type (Fig. 8b, d, f, g), we divided the granuloma into three zones centered on the egg: the inner zone (0–50 μm), middle zone (50–100 μm), and outer zone (100–150 μm). The inner zone was predominantly composed of Ly6G^+^/CD177^+^ cells, accounting for 20.3% to 36.2% of the total cells, whereas the proportions of both Ly6G^+^/LTF^+^ and Ly6G^+^/MMP9^+^ cells were less than 3%. The middle zone was mainly occupied by Ly6G^+^/LTF^+^ cells (17.9%–23.6%), with Ly6G^+^/CD177^+^ cells comprising 4.6% to 8.3% of the cells and Ly6G^+^/MMP9^+^ cells less than 3%. The outer zone was primarily enriched with Ly6G^+^/MMP9^+^ cells (23.1%–31.9%), accompanied by substantial proportions of Ly6G^+^/LTF^+^ (19.2%–31.4%) and Ly6G^+^/CD177^+^ (10.9%–20.0%) cells. Our analysis revealed clear spatial zonation of neutrophil subsets within *S. japonicum* egg–induced granulomas (Fig. 8h).

### Targeted silencing of *Ltf* and *Cd177* in neutrophils via AAV8-mediated RNAi

**Fig. 9:**
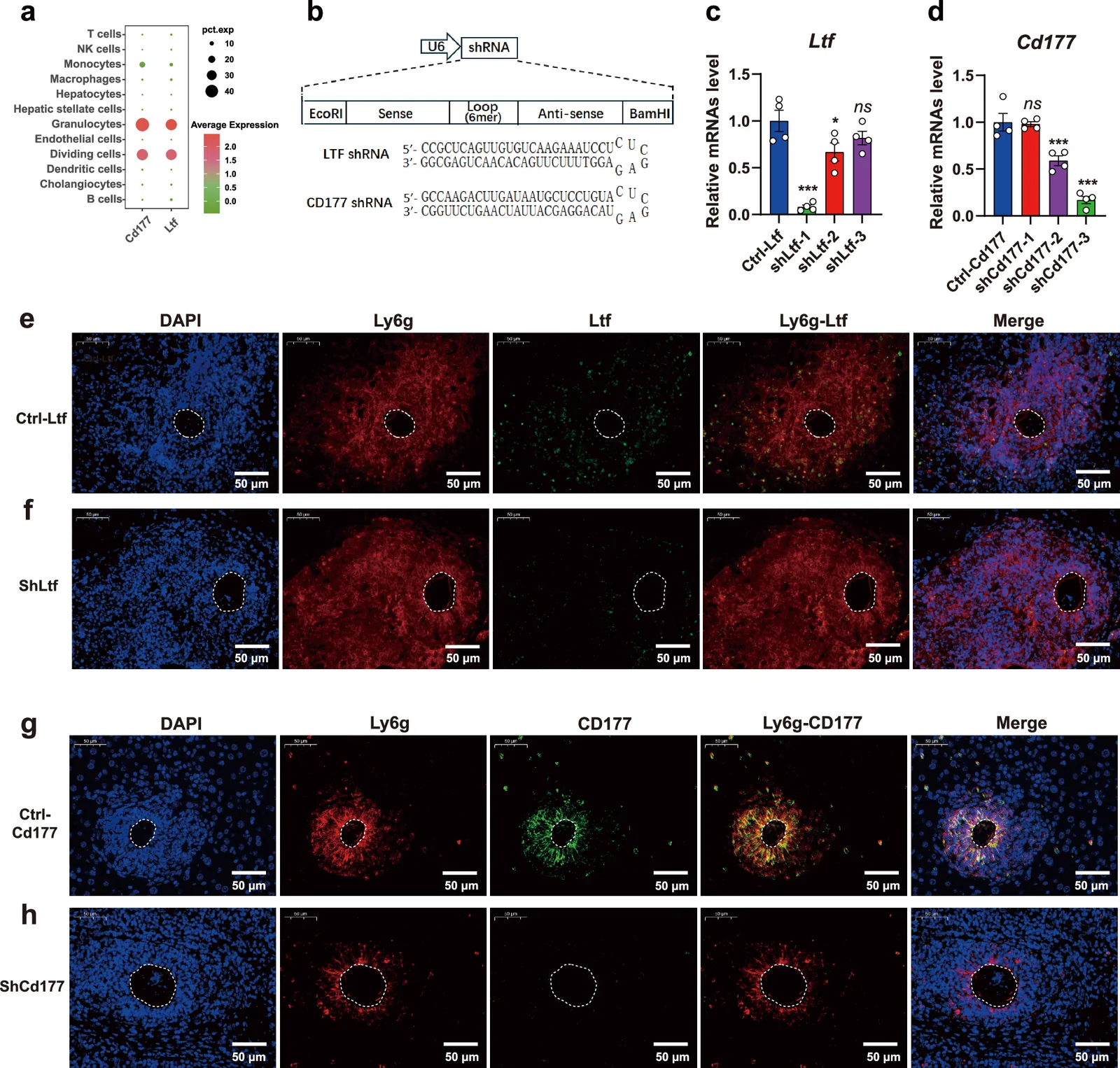
Targeted regulation of neutrophil subsets in *S. japonicum* egg granulomas via AAV8-mediated LTF and CD177 knockdown. **a** *Ltf* and *Cd177* expression in different cell clusters (combined scRNA‑seq data from 0 to 70 d.p.i.). **b** LTF and CD177 shRNA constructs. Relative *Ltf* (**c**) and *Cd177* (**d**) mRNA levels in the liver (*n* = 4 per group). Immunofluorescence staining of Ly6G^+^/LTF^+^ cells in the Ctrl-Ltf group (**e**) and shLtf group (**f**). Immunofluorescence staining of Ly6G^+^/CD177^+^ cells in the Ctrl-Cd177 group (**g**) and shCD177 group (**h**). Multiplex immunofluorescence and immunofluorescence staining experiments shown in Fig. 9e–h were performed using samples from four biologically independent mice, and representative images are shown. Similar results were obtained across all samples analyzed. Dashed lines outline the egg area. Scale bar = 50 μm. Data are presented as mean ± SEM. Statistical significance was determined by a two-sided unpaired *t-*test. *P < 0.05, ***P < 0.001 compared with the control group. Source data are provided as a Source Data file. Exact *P* values are provided in the Source Data file.

The unique spatial distributions of the Neu_C0_Ltf, Neu_C1_Mmp9, and Neu_C2_Cd177 subsets within granulomas suggest that they have distinct biological functions. To test this hypothesis, we knocked down the expression of subset-specific marker genes via RNA interference (RNAi). Notably, the Neu_C1_Mmp9 subset highly expresses both *Mmp9* and *Mmp8*, and these two matrix metalloproteinases share similar biological functions. *Mmp9* knockdown may thus fail to specifically abrogate the function of this subset. We thus selected *Ltf* and *Cd177* as the target genes for knockdown. Importantly, both *Ltf* and *Cd177* exhibit neutrophil-specific expression patterns (Fig. 9a), which conferred high specificity to the subsequent targeted knockdown experiments. We employed adeno-associated virus serotype 8 (AAV8)-mediated RNAi to silence *Ltf* and *Cd177* (Fig. 9b). One week after infection, mice received the AAV8 RNAi vectors via tail vein injection. Five weeks post-injection, the mice were euthanized. Liver tissue samples were collected directly, and hepatic neutrophils were isolated by flow cytometry. Quantitative real-time PCR (qRT-PCR) analysis showed that shLtf-1 and shCd177-3 significantly suppressed the expression of *Ltf* and *Cd177* in both liver tissue and isolated hepatic neutrophils (Fig. 9c, d and Supplementary Fig. 8a–c). Based on these results, mice treated with these two shRNAs were used for subsequent experiments. Immunofluorescence analysis confirmed that there was a significant reduction in the number of Ly6G^+^/LTF^+^ neutrophils in shLtf-1–treated mice and a marked decrease in Ly6G^+^/CD177^+^ neutrophils in shCd177-3–treated mice, further validating the efficiency of the gene silencing (Fig. 9e–h).

### LTF and CD177 exert opposing regulatory roles in regulating *S. japonicum* egg–induced hepatic granuloma formation and fibrosis

**Fig. 10:**
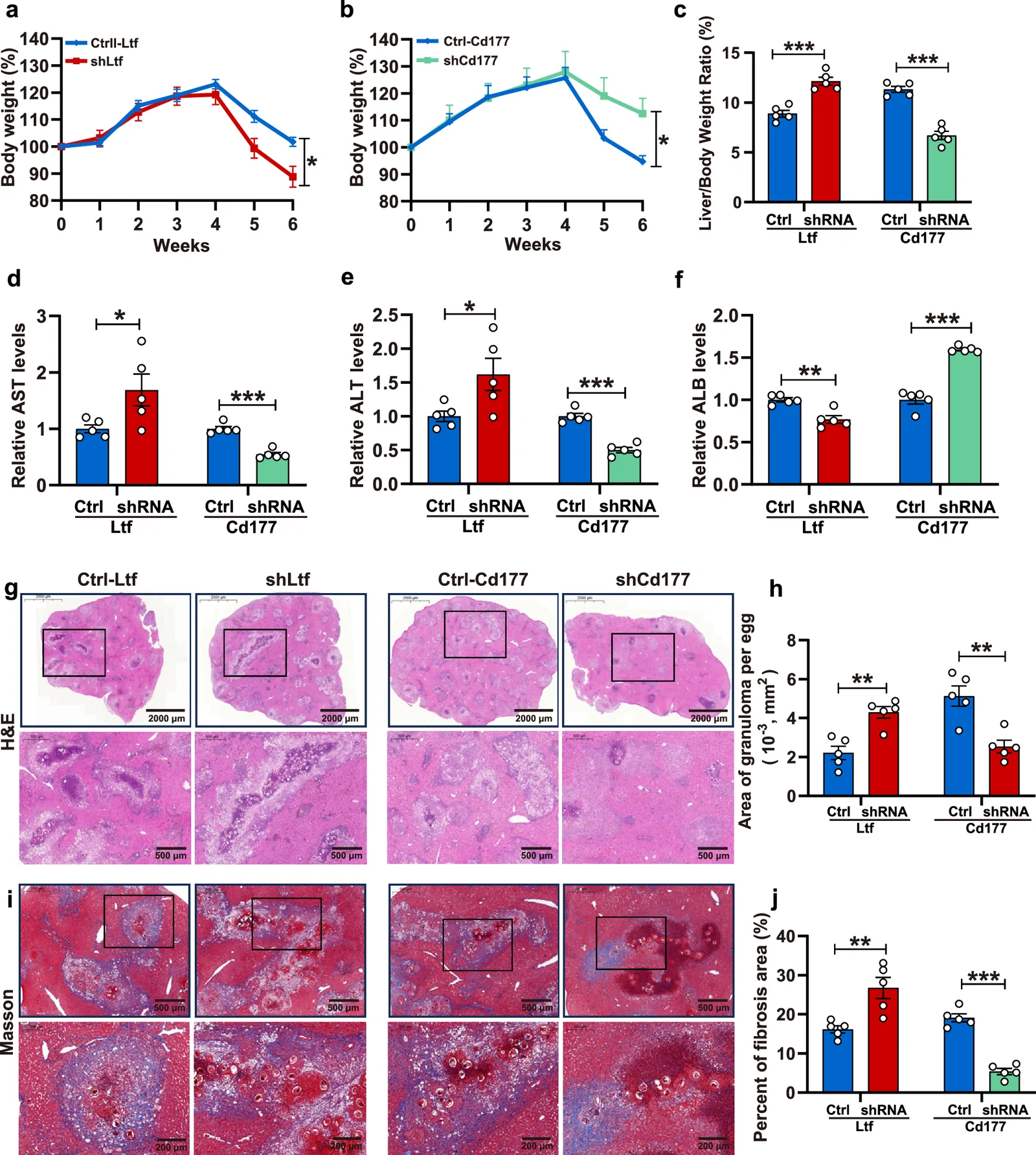
Assessment of hepatic injury and fibrosis in *S. japonicum*–infected mice following *Ltf* or *Cd177* knockdown. Changes in body weight of *Ltf* knockdown mice (**a**) and *Cd177* knockdown mice (**b**). **c** Liver-to-body weight ratio (liver index, %). Serum AST (**d**) and ALT (**e**) activity levels and ALB expression levels (**f**). **g** Representative H&E staining showing inflammatory infiltration and granuloma formation. Scale bars: 2000 μm (upper panel), 500 μm (lower panel). **h** Area of granuloma per egg. **i** Representative Masson’s trichrome staining showing collagen deposition. Scale bars: 2000 μm (upper panel), 500 μm (lower panel). **j** Quantification of fibrosis area. Values are expressed as mean ± SEM, *n* = 5 per group. Statistical significance was determined by two-sided unpaired *t* test. *P < 0.05, **P < 0.01, ***P < 0.001 compared with the control. Source data are provided as a Source Data file. Exact *P* values are provided in the Source Data file.

Mice in which *Ltf* or *Cd177* expression was knocked down exhibited distinct phenotypic changes compared with control mice. *Ltf* knockdown led to a significant reduction in body weight and a marked increase in the liver-to-body weight ratio (Fig. 10a, c). Serum biochemical analysis revealed a substantial elevation in ALT and AST levels, as well as a decrease in ALB (Fig. 10d–f). H&E and Masson’s trichrome staining showed that the granuloma area per egg (normalized to egg number) was significantly enlarged, in addition to exhibiting a marked increase in fibrosis severity (Fig. 10g–j). Collectively, these findings demonstrate that loss of *Ltf* expression exacerbates egg-induced hepatic injury. In contrast, silencing *Cd177* led to an increase in body weight and a reduction in the liver-to-body weight ratio (Fig. 10b, c). The serum ALT and AST levels decreased, while ALB levels increased (Fig. 10d–f). Moreover, both the granuloma area per egg and the extent of fibrosis were markedly attenuated (Fig. 10g–j). These findings were further corroborated by Sirius red staining and immunohistochemical staining for collagen I and α-smooth muscle actin (α-SMA), respectively (Supplementary Fig. 9a, b). These results indicate that CD177 and LTF expression are associated with opposing regulatory role**s** in *S. japonicum*–induced hepatic granuloma and fibrosis: CD177 expression promotes granulomatous inflammation and fibrosis, whereas LTF expression restrains these pathological processes.

## Discussion

This study provides a comprehensive characterization of the dynamic changes in immune cell populations within the liver throughout the course of *S. japonicum* infection. Previous scRNA-seq studies have preliminarily delineated the cellular landscape of schistosomiasis-associated liver fibrosis, identifying key immune subsets including Th2 cells, NKT cells, and macrophage subpopulations, as well as CCL–CCR and CXCL-CXCR signaling pathways critical for intercellular crosstalk^38,39^. However, these studies were largely based on static transcriptomic profiles from limited samples or focused on temporal changes without precise spatial information, leaving the in situ functional organization of myeloid cells poorly understood. By integrating scRNA-seq with spatial transcriptomic analysis, our work identifies a prominent expansion of neutrophils as a key hallmark following egg deposition. We further uncover a highly structured spatial organization of neutrophil subsets within developing granulomas and demonstrate their distinct, even opposing, functional roles in regulating granulomatous inflammation and fibrosis.

Granulomas induced by *S. japonicum* are predominantly neutrophilic, whereas those caused by *S. mansoni* are dominated by eosinophils^20,40^. Neutrophils serve as a major driver of hepatic necrosis and liver injury during the acute phase of *S. japonicum* infection, and also act as progenitors contributing to hepatic fibrosis development. Nevertheless, neutrophil-depleted mice infected with *S. japonicum* exhibit augmented hepatic granulomas throughout the infection, indicating a potential regulatory role of neutrophils in granuloma formation^41^. Moreover, the *S. japonicum* egg protein SjE16.7 has been verified to specifically recruit neutrophils to egg deposition sites in vivo, suggesting that parasite-derived molecules are critically involved in modulating neutrophil biological activity^42,43^. In vitro experiments further show that neutrophils release neutrophil extracellular traps (NETs) as early as 4 h after incubation with *S. japonicum* egg^44^. In contrast, the eosinophil response is far more prominent than the neutrophil response in *S. mansoni* egg-elicited granulomas. *S. mansoni* eggs secrete a chemokine-binding protein (smCKBP) that selectively interacts with specific host chemokines, thereby suppressing chemokine bioactivity and reshaping the cellular composition of granulomas. Mechanistically, smCKBP binds to the neutrophil chemoattractant CXCL8 and efficiently blocks neutrophil infiltration into granulomatous lesions, whereas it fails to recognize the eosinophil chemoattractant CCL11 and thus does not interfere with eosinophil recruitment^45^.

Consistent with the above findings, our single-cell transcriptomic analysis further confirms that neutrophils, rather than eosinophils, constitute the predominant cellular component of *S. japonicum*-induced granulomas, substantially advancing our understanding of neutrophil biology during helminth infection. We identified seven transcriptionally and functionally distinct neutrophil subsets, further highlighting the extensive heterogeneity of neutrophils^46–48^. Chemokine profiles showed that the neutrophil chemokine CXCL2 was highly expressed in granulomas, while eosinophil chemokines were barely detectable in any cell type. CXCL1 and CXCL2 potently recruit neutrophils by binding to the CXCR2 receptor in murine bacterial infection models^49–51^. Our data suggest that CXCL2 may play an important role in mediating neutrophil recruitment during *S. japonicum* infection. Notably, our scRNA-seq data demonstrate that neutrophils themselves highly co-express CXCL2 and its receptor CXCR2, suggesting that these cells maintain their dense accumulation within granuloma cores through an autocrine CXCL2-CXCR2 axis. In addition, *S. japonicum* eggs can directly stimulate human neutrophils to upregulate CXCL2 expression, highlighting the potent capacity of schistosome eggs to drive neutrophil inflammatory responses^44^. This chemokine axis may also operate in other immune cell types. A recent scRNA-seq study of PBMCs from patients with schistosomiasis japonica reported that CXCR2⁺ NKT cells are expanded in advanced disease and exhibit a pro-inflammatory signature^52^.

Earlier LMM-based transcriptomics described a dual neutrophil phenotype (pro-inflammatory core, reparative periphery) in *S. japonicum* granulomas^53^. Here, we further reveal the prominent and functionally meaningful spatial zonation of neutrophil subsets within early granulomas. We demonstrated that granulomas are highly organized structures with concentric stratification. Specifically, there is an inner zone that is enriched with CD177^+^ neutrophils, a middle zone that is dominated by LTF^+^ neutrophils, and an outer zone that consists primarily of MMP9^+^ neutrophils, as well as substantial proportions of LTF^+^ and CD177^+^ subsets.

CD177 is a specific surface marker highly expressed in neutrophils that mediates the recruitment, transepithelial migration, and activation of neutrophils^54–56^. In this study, we observed that CD177⁺ neutrophils displayed a distinct radial arrangement, tightly surrounding schistosome eggs. To investigate the functional role of CD177 in granuloma pathogenesis, we performed CD177 knockdown in infected mice. CD177 knockdown significantly attenuated hepatic granuloma formation and fibrosis severity, suggesting that CD177 promotes the progression of egg-induced granulomatous inflammation and fibrosis.

LTF is a secondary granule protein abundantly produced by neutrophils and is also detected in multiple exocrine secretions, including milk and tears^57^. LTF exerts diverse biological effects, including anti-inflammatory, anti-infective, and immunomodulatory activities, as well as roles in tissue regeneration^58–60^. In our model, LTF⁺ neutrophils were predominantly distributed in the mid-to-peripheral regions of early hepatic granulomas. Functional silencing of *Ltf* aggravated egg-triggered liver damage and fibrotic remodeling, supporting a protective, tissue-resolving function of LTF in schistosomiasis-associated hepatic lesions. Unlike the pro-inflammatory driver CD177, LTF may limit excessive immunopathology during granuloma development.

MMP9, together with MMP8, is a major matrix metalloproteinase abundantly expressed in neutrophils^61,62^. Both enzymes primarily mediate extracellular matrix degradation, tissue remodeling, and leukocyte migration during inflammation^63–65^. In the present study, MMP9⁺ neutrophils located in the outer zone of granulomas appear to play a pivotal role in local tissue remodeling. This degradative activity may contribute to fibrosis resolution in chronic schistosomiasis by breaking down fibrotic deposits and promoting subsequent tissue repair and remodeling.

Although AAV8 primarily transduces hepatocytes, the observed knockdown of *Ltf* and *Cd177* in neutrophils may be attributed to several contributing factors. First, the fibrotic microenvironment can enhance AAV8 transduction efficiency in immune cells^66^. Second, siRNA packaged into hepatocyte-derived exosomes may be transferred to neighboring cells, including newly recruited neutrophils, thereby influencing cells not directly transduced by AAV8^67,68^. Third, the inflamed tissue microenvironments significantly prolong neutrophil lifespan^26,69^. Together, these mechanisms likely contribute to the sustained knockdown effects observed in our study. However, further studies using neutrophil-specific conditional knockout mice are required to precisely clarify the direct roles and intrinsic contributions of CD177⁺ and LTF⁺ neutrophils in schistosome-induced hepatic granuloma formation and progression.

We next examined other immune populations. Single-cell analysis in this study revealed the temporal dynamics of hepatic macrophage polarization during *S. japonicum* infection, providing key insights into the formation of granulomatous immune microenvironments. We identified two functionally distinct macrophage subsets, M1-like Macro_C3_Cxcl9 and M2-like Macro_C2_Arg1, which exhibited significant population dynamics across different infection stages. In the pre-egg phase, M1 macrophages dominated, reflecting a classical Th1-skewed response against schistosomula^70^. By the egg-deposition phase, this ratio had normalized rapidly, followed by a striking shift toward M2 dominance that is consistent with the well-established immune deviation paradigm in schistosomiasis, wherein the immune response transitions from an early Th1-dominant to a late Th2-dominant profile^1,71^. M2 macrophages promote tissue repair via Arg1-mediated polyamine synthesis, but can also exacerbate fibrosis^72^. Their dominance correlated with peak granuloma size and collagen deposition in our model. High expression of CD9, TREM2, and SPP1 was observed in the Macro_C2_Arg1 subset, mirroring the transcriptional signature of pro-fibrotic macrophages in human cirrhosis and murine CCl₄-induced fibrosis^31^. This conservation implies a common pro-fibrotic program underlying liver fibrosis of different etiologies.

The identification of Mono_C1_Cxcl2 as a post-egg stage–specific monocyte subset sheds light on the presence of an orchestrated cascade of neutrophil recruitment. This process comprises two phases: an initial monocyte-driven phase, where Mono_C1_Cxcl2 initiates early recruitment, and a subsequent self-sustaining, neutrophil-dominated autocrine phase. We speculate that CD177^+^ neutrophils are among the first to be recruited to granulomas, which is supported by their spatial localization adjacent to eggs and the ability of CD177 to promote neutrophil transmigration^54^.

In this study, the reduction in the number of T cells retrieved for analysis following egg deposition limited precise discrimination of all T cell subsets (e.g., Tfh and Th9 cells) and detailed characterization of their dynamic changes. Nevertheless, our key findings are consistent with well-established immunological paradigms in schistosomiasis^71,73–75^. Our results revealed a well-characterized immunological shift following egg production: a significant expansion of Th2 cells. This observation confirms the classical model that SEA induces a switch in the host immune response^76,77^. Pathway enrichment analysis provided additional evidence to support this immunopathology. The IL-17 signaling pathway was significantly enriched after egg deposition, which is consistent with the pro-inflammatory role of Th17 cells and IL-17 in chronic inflammation and granuloma development^35,78,79^. IL-10 was also significantly upregulated following egg deposition. IL-10 is known to exert crucial immunosuppressive effects by restraining excessive immune responses^80,81^. In schistosomiasis, IL-10 limits the severity of egg-induced granulomatous inflammation and hepatic fibrosis^82–84^. Although we did not identify which cell type secretes the highest level of IL-10, previous studies have reported that Th2 cells and Tregs serve as the major cellular sources of IL-10 during schistosome infection^82,84–86^. Additionally, we detected a significant expansion of plasma cells, suggesting that antibodies secreted by plasma cells may exert a neutralizing effect against SEA during the chronic phase of infection^87^. Dynamic changes in the proportions of cDC1 and pDC before and after egg deposition suggest that cDC1 plays a key role in presenting schistosomula antigens and priming Th1 immune responses^88^, whereas pDC is closely associated with the regulation of chronic inflammatory responses, consistent with previous reports in *S. mansoni* infection^37^.

*Schistosoma* granuloma formation is a dynamic process that has also been observed in other types of granulomas, such as those associated with tuberculosis^89,90^. In this study, we observed granuloma heterogeneity not only at different disease stages, but also at the same time points. Overall, as the disease progressed, the number of HSCs (Desmin^+^ cells) gradually increased, whereas the number of neutrophils (Ly6G^+^ cells) progressively decreased. However, due to the continuous production of fresh eggs, neutrophil-dominant granulomas remained detectable in the chronic phase. Based on the proportional differences in Ly6G^+^, F4/80^+^, and Desmin^+^ cells, we delineated a three-stage evolutionary trajectory for schistosome-induced hepatic granulomas: early granulomas (Ly6G^hi^F4/80 ^l^°Desmin^l^°), developing granulomas (Ly6G^mid^F4/80^hi^Desmin^mid^), and advanced granulomas (Ly6G^l^°F4/80^mid^Desmin^hi^). Early granuloma formation is induced by fresh eggs, and was detectable at both 42 and 70 d.p.i. However, the proportion of Ly6G^+^ cells at 70 d.p.i. was lower than that at 42 d.p.i. The proportion of macrophages (F4/80^+^) in developing granulomas was significantly higher than that in the other two types of granulomas, suggesting that macrophages may be involved in clearing apoptotic neutrophils at this stage. Advanced granulomas correspond to lesions surrounding dead schistosome eggs, which are densely populated with activated HSCs. This classification will facilitate systematic analysis of the dynamic pathological progression of schistosome hepatic granulomas, from formation to maturation. Importantly, our study highlights that, in addition to grouping granulomas by infection time points, attention should be paid to granuloma heterogeneity within the same time point when investigating schistosome egg–induced granulomas and fibrosis.

This study has several technical limitations. First, advanced hepatic fibrosis may bias single-cell recovery: cells from loosely organized, neutrophil-rich granulomas are preferentially released during tissue dissociation, whereas cells within dense, fibrotic granulomas are underrepresented. Consequently, scRNA-seq (Fig. 1c) and flow cytometry (Supplementary Fig. 10) may overestimate neutrophil proportions at late fibrotic stages and potentially yield results that are inconsistent with tissue-based quantification (Fig. 7c), which does not require cell release and thus more faithfully represents the in situ cellular composition. Second, the low number of HSCs detected in our scRNA-seq data reflects a known technical challenge in fibrotic liver studies: HSCs are fragile due to their stellate morphology and lipid-rich cytoplasm, making them prone to rupture during enzymatic and mechanical dissociation. Future investigations combining single-nucleus sequencing and cell-specific pre-isolation strategies will help circumvent dissociation-related bias and enable a more comprehensive profiling of stromal cell populations involved in schistosomiasis-associated fibrosis.

## Methods

### Ethics

All animal experiments were carried out in accordance with the Guide for the Care and Use of Laboratory Animals of the National Institutes of Health. The experiments were approved by the Committee for Animal Research of Tongji University (TJAA11422101). The study was conducted in accordance with the local legislation and institutional requirements.

### Animals, parasites and experimental design

Specific-pathogen-free (SPF) C57BL/6J female mice, 6 to 8 weeks old, weighing 20 to 25 g, were provided by Beijing Siberian Biotechnology Co., Ltd. They were supplied with sterile food and water ad libitum and housed in the Biosafety Level-2 (BSL-2) Laboratory of Tongji University. *S. japonicum* cercariae (strain isolated in Jiangsu, China), hatched from infected *Oncomelania hupensis*, were provided by the Department of Snail Biology, Jiangsu Institute of Parasitic Diseases. Each mouse was infected with 30 ± 2 cercariae. Cercariae with strong viability were collected on a coverslip and counted under a dissecting microscope. The coverslip was then placed on the bare abdomen of the mice for at least 15 minutes to allow cercarial invasion. All mice were deeply anesthetized with 2% pentobarbital before serum and liver samples were collected.

For body weight monitoring and survival analysis, a total of 25 mice were used, including 5 uninfected control mice and 20 infected mice. One infected mouse that failed to develop a patent infection (no adult worms or eggs detected at necropsy) was excluded from subsequent analyses. Body weight was measured weekly, and survival status was monitored daily until 70 d.p.i.

For serum liver function detection and histopathological evaluation, an independent cohort of 50 mice was used. Six uninfected mice were euthanized at day 0 to serve as baseline controls. The remaining 44 mice were subjected to *S. japonicum* infection, and six mice were euthanized at each indicated time point (16, 22, 28, 42, 56, and 70 d.p.i.). An additional eight infected mice were reserved to replace individuals with unexpected death during the experiment.

For liver wet weight measurement and scRNA-seq analysis, a third independent cohort consisting of 40 mice was used. At day 0 and each post-infection time point (16, 22, 28, 42, 56, and 70 d.p.i.), five mice were euthanized: three for liver wet weight measurement, and two for in situ perfusion and subsequent NPC isolation for scRNA-seq. The other five infected mice were reserved as spare individuals. Detailed protocols for NPC isolation, library construction and transcriptome sequencing are described in the following sections.

### Biochemical analysis

Serum was isolated by centrifuging the collected blood at 1500 g for 15 minutes at room temperature. Alanine transaminase (ALT), aspartate aminotransferase (AST), and albumin (ALB) were detected using a Chemray 240 automatic biochemical analyzer (Shenzhen Rayto Life and Analytical Sciences Co., Ltd., Shenzhen, China).

### Liver histopathology and immunohistochemistry

Liver samples from the left lateral lobe were cut into 10 mm × 10 mm × 1 mm pieces and fixed in 4% paraformaldehyde following standard protocols and subsequently sliced into 4-μm-thick continuous sections. The samples were stained with hematoxylin and eosin (H&E), Masson’s trichrome, and Sirius red to assess the percentages of granulomatous and fibrotic areas. The sections were observed under a microscope and digitized using an imaging system (Olympus, Japan), and all images were analyzed with Image Pro Plus 6.0 software (Media Cybernetics, Rockville, MD, USA). HSC proliferation was evaluated via immunohistochemistry (IHC). Specifically, 4-μm serial sections of paraffin-embedded tissue from each mouse were stained for OPN, ki67, α-SMA, and collagen I as follows. The liver sections were first deparaffinized and hydrated, then subjected to antigen retrieval by heating in citric acid buffer at 95 °C for 10 min, followed by blocking with 5% BSA for 30 min. The slides were then incubated overnight at 4 °C in a humidified chamber with primary antibodies against OPN (1:600, GB11500, Servicebio, Wuhan, China), ki67 (1:300, GB111499, Servicebio), α-SMA (1:500, GB111364, Servicebio), or collagen I (1:1000, GB110223, Servicebio). The sections were then incubated with HRP-conjugated goat anti-rabbit IgG (1:500, G1302, Servicebio) for 1 hour at 25 °C. After washing with PBS, DAB (1:200, ZSGB–BIO, Beijing, China) was added as the enzyme substrate. Five high-power fields (× 400 magnification, Olympus, Japan) were randomly selected from each stained section for each mouse, and the integrated optical density (IOD) for OPN, Ki-67, α-SMA, and collagen I was calculated using Image-Pro Plus 6.0 software, with a higher IOD value indicating stronger expression.

### scRNA-seq analysis of liver NPCs

Livers were perfused in situ with 100 ml calcium-free Hank’s Balanced Salt Solution (HBSS) containing 0.2 mg/mL EDTA, followed by sequential perfusion with 5 ml HBSS containing 0.4 mg/mL pronase (Sigma, P5147) and 0.2% collagenase type II (Worthington, LS004196). The livers were then removed, minced, and digested in HBSS containing 0.4 mg/mL pronase, 0.2% collagenase type II, and 0.1 mg/mL DNase I (Roche, R104159001) at 37 °C with shaking for 10 min. Digestion was terminated by adding DMEM supplemented with 10% serum. The resulting cell suspensions were centrifuged at 50 × *g* for 3 min to remove hepatocytes, then filtered through a 30-μm nylon cell strainer and treated with ACK Lysis Buffer (BD Biosciences, 555899) to lyse red blood cells. The non-parenchymal cell (NPC) suspensions were subsequently centrifuged and resuspended in HBSS, and a dead cell removal kit (Miltenyi Biotec, 130090101) was used to eliminate dead cells. Cell viability was confirmed by AO/PI dual fluorescence staining detected on a Countstar Rigel (S2) instrument. Quality control was performed using a cell counter, and the concentration was adjusted to 700 to 1200 cells/μL, with cell viability greater than 90% and aggregation less than 15%. Then, the cells were placed on ice, and a 10× Genomics single cell transcriptome chip on-board assay was carried out within 30 min.

### scRNA-seq library preparation and sequencing

scRNA-seq libraries were prepared using Chromium Next GEM Single Cell 3ʹ Reagent v3.1 kits on a Chromium Controller (10× Genomics). The cell suspension was loaded onto a Chromium Next GEM Chip G, and the Chromium Controller was run to generate gel beads-in-emulsion (GEMs) containing individual single cells, following the manufacturer’s protocol. The captured cells were lysed, and the released RNA was barcoded through reverse transcription of individual GEMs. Barcoded, full-length cDNA was then synthesized, and libraries were constructed according to the manufacturer’s protocol. Library quality was evaluated using two complementary methods: the library concentration was quantified with a Qubit 4.0 fluorometer (Thermo Fisher Scientific), and the library size distribution was analyzed using an Agilent 2100 Bioanalyzer (Agilent Technologies) to confirm a major peak at 400 to 600 bp (a characteristic of qualified 10× scRNA-seq libraries) and exclude impurity peaks, such as those indicating the presence of adapter dimers. Sequencing was performed on an Illumina NovaSeq 6000 platform by Biomarker Technologies Corporation (Beijing, China), with a sequencing depth of at least 50,000 reads per cell and 150 bp paired-end reads (PE150).

### Data processing and quality control

Raw scRNA-seq data were processed using CellRanger (10x Genomics) for demultiplexing, alignment to the mouse reference genome (mm10), and generation of gene-barcode matrices. Downstream analyses were performed in Seurat (v4.3.0)^91^. Cells with fewer than 200 detected genes or with >10% mitochondrial gene expression were excluded. Normalization and variance stabilization were performed using the SCTransform function. Dimensionality reduction was performed by principal component analysis (PCA), and clusters were identified using the Louvain algorithm, and resolution parameters were optimized through silhouette scoring to balance cluster granularity and biological interpretability. Cell type annotation was carried out based on canonical marker genes and cross-referenced against curated resources, including CellMarker and PanglaoDB, to ensure high-confidence assignments. Differentially expressed genes (DEGs) in each cluster and across disease phases were identified using Wilcoxon rank-sum testing with Bonferroni’s correction.

### Clustering and visualization

Uniform manifold approximation and projection (UMAP) embeddings were generated using the top principal components. Cell clustering was performed with the Louvain algorithm at a resolution of 0.6^92^. Cell types were annotated based on canonical marker genes and DEGs. Expression patterns of selected markers (e.g., *Ltf*, *Mmp8*, *Cd177*, *Marco*, *Vsig4*, and *Cxcl* family chemokines) were visualized using dot plots, violin plots, heatmaps, and feature plots.

### Differential gene expression and pathway enrichment

Cluster-specific DEGs were identified using the Wilcoxon rank-sum test with Bonferroni correction (adjusted *P* < 0.05). Functional enrichment was performed with clusterProfiler (v4.6.0)^93^ against the Gene Ontology (GO) and KEGG pathway databases. Enriched pathways (FDR < 0.05) were visualized as bar plots to highlight biological functions such as oxidative phosphorylation, antigen presentation, immune activation, and phagocytosis.

### Developmental potential and trajectory inference

Cell differentiation potential was assessed with CytoTRACE (v0.3.3)^94^, and CytoTRACE scores were compared across neutrophil subclusters. RNA velocity analysis was performed using velocyto (v0.17.17)^95^ to quantify spliced and unspliced transcripts, followed by scVelo (v0.2.5)^96^ dynamic modeling to reconstruct transcriptional dynamics and latent time. Lineage trajectories were inferred using partition-based graph abstraction (PAGA).

### Pseudotime-dependent gene expression

Pseudotime trajectories^97^ were used to identify genes whose expression levels were dynamically regulated along differentiation lineages. Hierarchical clustering grouped genes into co-expression modules, which were visualized as heatmaps. Genes with significant pseudotime-dependent regulation (FDR < 0.05) were reported.

### Temporal dynamics of immune cell subsets

Cells from sequential infection timepoints ranging from 0 to 70 d.p.i. were projected onto the reference UMAP and assigned to previously defined clusters^91^. The proportions of the neutrophil, monocyte, macrophage, and Kupffer cell subtypes were calculated for each time point, and temporal abundances were visualized as stacked bar plots. Similar approaches were applied to quantify chemokine (Cxcl) gene expression dynamics across the immune and non-immune compartments, including hepatocytes, HSCs, cholangiocytes, and dividing cells.

### Signature scoring

Functional polarization states were assessed using curated gene sets for M1, M2, and lipid-associated macrophage (LAM) signatures. Additionally, pro-inflammatory (*Il1b*, *Il6*, *Tnf*) and anti-inflammatory (*Il10*, *Tgfb1*) modules were scored. Per-cell signature enrichment was calculated using AUCell (v1.18.1) and Seurat’s AddModuleScore^98,99^. Group comparisons were performed by the Kruskal-Wallis test.

### Cell–cell communication analysis

CellChat (v1.5.0) was used to explore intercellular signaling, which infers communication networks using a ligand–receptor interaction database^100^. The normalized expression matrix and cell type annotations from Seurat were input into CellChat. The communication probability (Comm.Prob) was estimated using a mass-action model that accounts for the expression of both ligands and their cognate receptors across interacting cell populations^100^. Circle plots were generated to display the number and strength of inferred interactions across cell types. To quantify communication patterns, incoming and outgoing interaction strengths were represented using scatter plots. Cell–cell signaling networks were inferred separately for the pre-egg phase and the acute/chronic phases of infection. Differential pathway activity was assessed by comparing Comm. Prob values between conditions. Significantly upregulated or downregulated pathways (*P* < 0.05 by permutation test) were visualized using dot plots, in which dot size represented significance and color intensity indicated communication probability. To further dissect specific pathways of interest, such as the CXCL-CXCR2 signaling axis, phase-specific communication probability matrices were extracted. Heatmaps displaying sender–receiver interactions were generated to assess context-dependent differences in CXCL-mediated signaling. In addition, chord diagrams were constructed to depict the directional information flow between source and target cell types, to enable visualization of altered communication hierarchies across disease phases.

### Data visualization and reproducibility

Network graphs, heatmaps, dot plots, and chord diagrams were generated using a combination of CellChat^100^, igraph (v1.5.1), and ggplot2 (v3.4.4) in R^101^. To ensure comparability across experimental groups, all communication probability matrices were normalized, and all visualizations were scaled, using identical parameters. Figures corresponding to global communication networks, phase-specific pathway enrichment, and CXCL signaling flow were generated using this unified pipeline.

### Microscopy and quantitative image analysis

High-resolution whole-tissue imaging was performed using the TissueFAXS automated microscopy platform (TissueGnostics GmbH, Vienna, Austria), which enables acquisition of large-area, multi-channel images with minimal signal loss across scanned sections. For multiplex immunofluorescence staining, filter settings were individually optimized for each target antigen to maximize the signal-to-noise ratio and to minimize spectral overlap. The following primary antibodies were employed: Desmin (ab32362, Abcam) for stromal cells, F4/80 (GB113373, Servicebio) for macrophages, CD3 (ab237721, Abcam) for T lymphocytes, CD19 (ab245235, Abcam) for B lymphocytes, and Ly6G (GB11229, Servicebio) for neutrophils. DAPI was used for nuclear counterstaining to facilitate single-cell segmentation.

Quantitative image analysis was performed using TissueQuest software (TissueGnostics), which provides a flow cytometry-like analytical framework applied directly to intact tissue sections, thereby preserving the spatial organization of cellular subsets. Single-cell identification was achieved through automated segmentation, followed by classification based on fluorophore intensity. To ensure objective and reproducible identification of positive cells, fluorescence thresholds were stringently determined using positive control tissues, defined as the mean fluorescence intensity (MFI) plus two standard deviations.

For advanced six-color multispectral analysis, spectral libraries were first established from singly stained reference tissues. These libraries were then used in StrataQuest software (TissueGnostics, version 7.1.129) for spectral unmixing, which effectively disentangles overlapping emission spectra and eliminates fluorophore crosstalk. This computational step guaranteed accurate channel separation and specific attribution of fluorescence signals to their corresponding markers. The resulting datasets enabled reliable quantitative assessment of immune and stromal cell populations within the native tissue microenvironment, while retaining both phenotypic detail and spatial context.

### Preparation of AAV-mediated RNA interference viruses targeting Ltf and Cd177

To knock down *Ltf* and *Cd177* expression, the siRNA expression constructs (5’-BamHI-[siRNA sense strand]-loop (CTCGAG)-[siRNA antisense strand]-terminator (TTTTT)-EcoRI-3’) were cloned into pHBAAV-U6-MCS-CMV-EGFP (Hanbio Biotechnology, Shanghai, China), placing siRNA expression under the control of a U6 promoter. The three siRNA sequences used for *Ltf* were: siRNA-Ltf-1: 5’-CCGCTCAGTTGTGTCAAGAAATCCT-3’, siRNA-Ltf-2: 5’-CGGAAGACTGCATTGTCGCAATCAT-3’, and siRNA-Ltf-3: 5’-GCAAGTGCGGTTTAGTTCCAGTCTT-3’. The three siRNA sequences used for *Cd177* were: siRNA-Cd177-1: 5’-CAGGACAATATTGGTGTCTCCATAT-3’, siRNA-Cd177-2: 5’-GGGTCATTTCTGATCTGAAGGTACA-3’, and siRNA-Cd177-3: 5’-GCCAAGACTTGATAATGCTCCTGTA-3’. The recombinant plasmids were verified by sequencing and then individually co-transfected into AAV-293 cells with pAAV-RC and pHelper via liposome-mediated transfection. Viruses were collected 72 h post-transfection, purified using a Biomiga AAV Purification Kit (Cat. No. V1469-01), and concentrated to a titer of 1 × 10^12^ to 2 × 10^12^ vector genomes (vg)/mL. The virus aliquots were stored at −80 °C until use. U6 siRNA control AAVs were provided by Hanbio Biotechnology.

### AAV-mediated knockdown of Ltf and Cd177 in S. japonicum–infected mice

Mice were infected with 30 ± 2 *S. japonicum* cercariae. Five mice were used in each experimental group. One week post-infection, each mouse received a tail vein injection of 100 μL PBS containing 2 × 10^12^ vg of AAV. The mice were euthanized at 42 d.p.i. Liver tissue was digested to prepare single-cell suspensions. Ly6G^+^ neutrophils were isolated via fluorescence-activated cell sorting. Total RNA was extracted from liver tissues and sorted cells, and qRT-PCR was performed to detect *Ltf* and *Cd177* expression levels using the following primers: Ltf-F: 5’-TGAGGCCCTTGGACTCTGT-3’, Ltf-R: 5’-ACCCACTTTTCTCATCTCGTTC-3’, Cd177-F:5’-ATACCAGTGCTGACCCTTCTG-3’, and Cd177-R: 5’-CCTCGCAGGTTTTCTCACCA-3’. *Gapdh* was used as the internal reference gene, and relative mRNA expression was calculated using the 2^–ΔΔCT^ method. Serum biochemical analysis, liver pathology measurement, and liver immunohistochemical analysis were performed as described above.

### Fluorescence-activated cell sorting and flow cytometry analysis

Intrahepatic leukocytes were obtained as described elsewhere, with minor modifications^102^. Briefly, liver tissues from individual mice were minced and mechanically dissociated by gently pressing through a 40-µm nylon cell strainer using a sterile syringe plunger. The cell suspension was centrifuged at 300 g for 10 min and resuspended in 4 ml of 40% Percoll (GE Healthcare, catalog number: 17-0891-01), which was carefully overlaid onto 5 ml of 80% Percoll in a new tube. After centrifugation at 700 g for 25 min, leukocytes were collected from the interface between the 40% and 80% Percoll layers. Red blood cells were lysed using ACK Lysis Buffer (BD Biosciences, catalog number: 555899). For flow cytometry staining, approximately 1 × 10⁷ cells were incubated with fluorochrome-conjugated antibodies against surface markers, including anti-Ly6G (PE) and anti-CD11b (APC) (561104 and 564985, BD PharMingen), according to the manufacturer’s instructions. Cells were stained on ice for 30 min in the dark, washed, and resuspended in FACS buffer. For fluorescence-activated cell sorting (FACS), cells were analyzed and sorted using a BD FACSAria III flow cytometer (Becton Dickinson). For flow cytometry analysis, data acquisition was performed on a BD LSRFortessa flow cytometer (Becton Dickinson), and data were analyzed using FlowJo_10.8.1 software (Tree Star).

### Immunofluorescence staining of paraffin-embedded liver sections

Liver tissues were fixed in 4% paraformaldehyde, dehydrated, embedded in paraffin, and sliced into 4- to 5-µm sections. The sections were baked at 60 °C for 30 min, deparaffinized in xylene, and rehydrated with a graded ethanol series. Heat-induced epitope retrieval was performed in citrate buffer (10 mM, pH 6.0), followed by cooling to room temperature and rinsing in PBS. For permeabilization, sections were incubated in 0.2% Triton X-100 for 5–10 min, then blocked for 30 min at room temperature in blocking buffer containing 5% normal serum (matched to the secondary antibody host species) and 1% bovine serum albumin. Primary antibodies against Ly6G (1:500, GB11229, Servicebio, Wuhan, China), Ltf (1:500, GB112032, Servicebio), CD177 (1:500, GB11316, Servicebio), MMP9 (1:500, GB150096, Servicebio), and CXCL2 (1:500, ab317569, Abcam) (raised in distinct host species to enable multiplex staining) were diluted in antibody diluent (1% BSA, 0.05% Tween-20, PBS) at optimized working concentrations, and the sections were incubated with the diluted antibodies overnight at 4 °C in a humidified chamber. The following day, the sections were washed in PBS with 0.05% Tween-20 (3 × 5 min) and incubated with species-specific, cross-adsorbed secondary antibodies conjugated to spectrally distinct fluorophores (e.g., Alexa Fluor 488, 555, and 647) for 60 min at room temperature, protected from light. Nuclei were counterstained with DAPI, and the sections were mounted on slides using an anti-fade mounting medium (Servicebio). Images were acquired on a confocal laser-scanning microscope using 405, 488, 561, and 640 nm laser lines. Consistent acquisition parameters (laser intensity, gain, and offset) were used for all experimental groups. For each sample, at least three randomly selected fields were imaged. Image processing and quantification were performed using ImageJ, applying identical thresholds and background subtraction to all images. For each ROI, the CXCL2 channel was converted to 8-bit grayscale, and the mean fluorescence intensity (mean gray value) was measured. Colocalization and spatial distance analyses were conducted using standard plugins, and negative controls (isotype controls and omission of primary antibodies) were included to validate staining specificity. Granulomatous regions were defined as areas surrounding *S. japonicum* eggs characterized by dense inflammatory cell infiltration and organized granuloma structure, as identified based on DAPI staining and tissue morphology. Non-granulomatous regions were defined as liver parenchymal areas located at least 200–500 μm away from visible granulomas and lacking inflammatory cell aggregation.

### Statistics and reproducibility

All values are expressed as mean ± SEM. The data were analyzed using SPSS 19.0 software (SPSS Inc, Chicago, IL, USA). Student's *t-*test was used for comparison between two groups. One-way ANOVA with Tukey’s post hoc test was used for comparison among more than two groups. Statistically significant differences were defined by *P* < 0.05. Statistical approaches for bioinformatic analyses are described in the corresponding bioinformatics section of Methods. Multiplex immunofluorescence and immunofluorescence staining experiments shown in Figs. 6i and 8a–f; 9e–h were performed using samples from 4 biologically independent mice, and representative images are shown. Similar results were obtained across all samples analyzed. For Fig. 7a, whole-section multiplex immunofluorescence images from liver sections collected at D28, D42, and D70 post-infection are shown. For Fig. 7b, representative images of distinct granuloma types are shown. Quantitative analyses were performed on individual granulomas, with *n* = 20 (28-I), *n* = 25 (42-I), *n* = 4 (42-II), *n* = 2 (42-III), *n* = 7 (70-IV), *n* = 2 (70-V), and *n* = 16 (70-VI).

### Reporting summary

Further information on research design is available in the Nature Portfolio Reporting Summary linked to this article.

## Supplementary information

Supplementary Information

Description Of Additional Supplementary File

Supplementary Data 1

Supplementary Data 2

Supplementary Data 3

Supplementary Data 4

Supplementary Data 5

Reporting summary

Transparent Peer Review file

## Source data

Source data

## Data Availability

The single-cell RNA sequencing data generated in this study have been deposited in the Genome Sequence Archive (GSA) database under accession code CRA029043. These sequencing data are publicly available without restricted access. All source data underlying the figures generated in this study are provided in the Source Data files, and other supporting datasets are available within the Supplementary Information. Source data are provided with this paper.
