# Supplementary material for: Cellular heterogeneity of hepatic granuloma formation and evolution in murine schistosomiasis japonica
Source: Nat Commun. 2026 Jul 8;17:8697. doi: 10.1038/s41467-026-75112-7 (PMC13490380; doi:10.1038/s41467-026-75112-7)
Supplement: Supplementary file 1 [file 41467_2026_75112_MOESM1_ESM.pdf]

Supplementary Figures

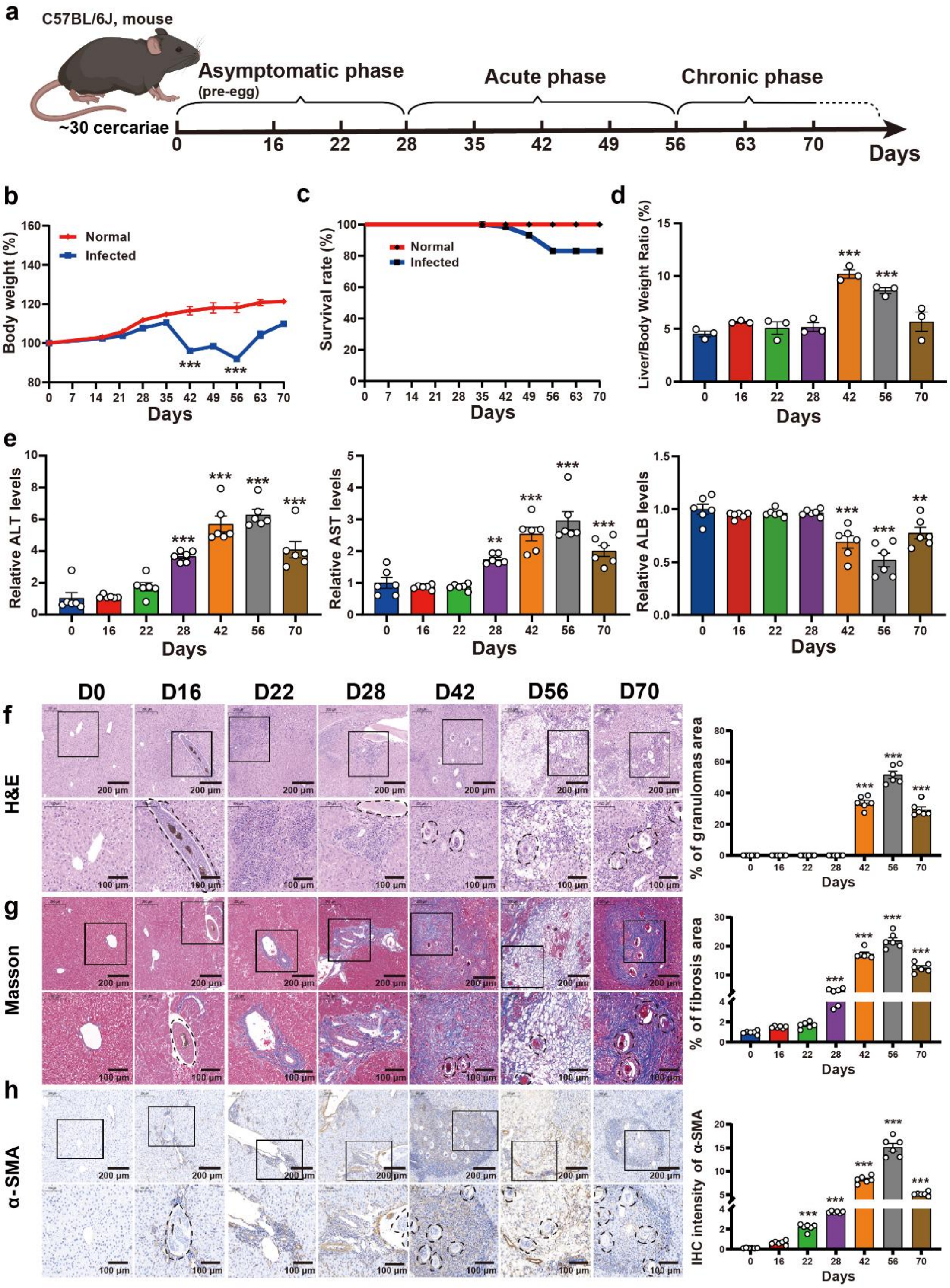

Supplementary Fig. 1. Liver injury and fibrosis in mice infected with *S. japonicum* over time. (a) Key stages in murine *S. japonicum* infection (Created in BioRender. Cao, G. (2026) https://BioRender.com/hilmkhl). (b) Body weight normalized to the baseline

value at D0. Normal group, n = 5; Infected group, n = 19. (c) Survival rate of mice following *S. japonicum* infection. Normal group, n = 5; Infected group, n = 19. (d) Liver-to-body weight ratio (liver index, %). n = 3 per group. (e) Serum levels of alanine aminotransferase (ALT), aspartate aminotransferase (AST), and albumin (ALB) (normalized to D0 control levels). n = 6 per group. Representative images of hepatic histopathology, as assessed by (f) H&E staining (inflammatory cell infiltration and granuloma formation), (g) Masson's trichrome staining (collagen deposition and fibrosis), and (h) α-smooth muscle actin (α-SMA) immunohistochemistry (activated hepatic stellate cells). Schistosome eggs and schistosomula are circled with dashed lines. Scale bars: 200 μm (main panels) and 100 μm (magnified insets). n = 6 per group. Granuloma area (from H&E staining), fibrosis area (from Masson's trichrome staining), and α-SMA immunohistochemistry (IHC) intensity scores were quantified using Image-Pro Plus software. Data are presented as mean ± SEM. Statistical significance was determined by one-way ANOVA with multiple comparisons. ** $P < 0.01$, *** $P < 0.001$ vs. D0 group. Source data are provided as a Source Data file. Exact P values are provided in the Source Data file.

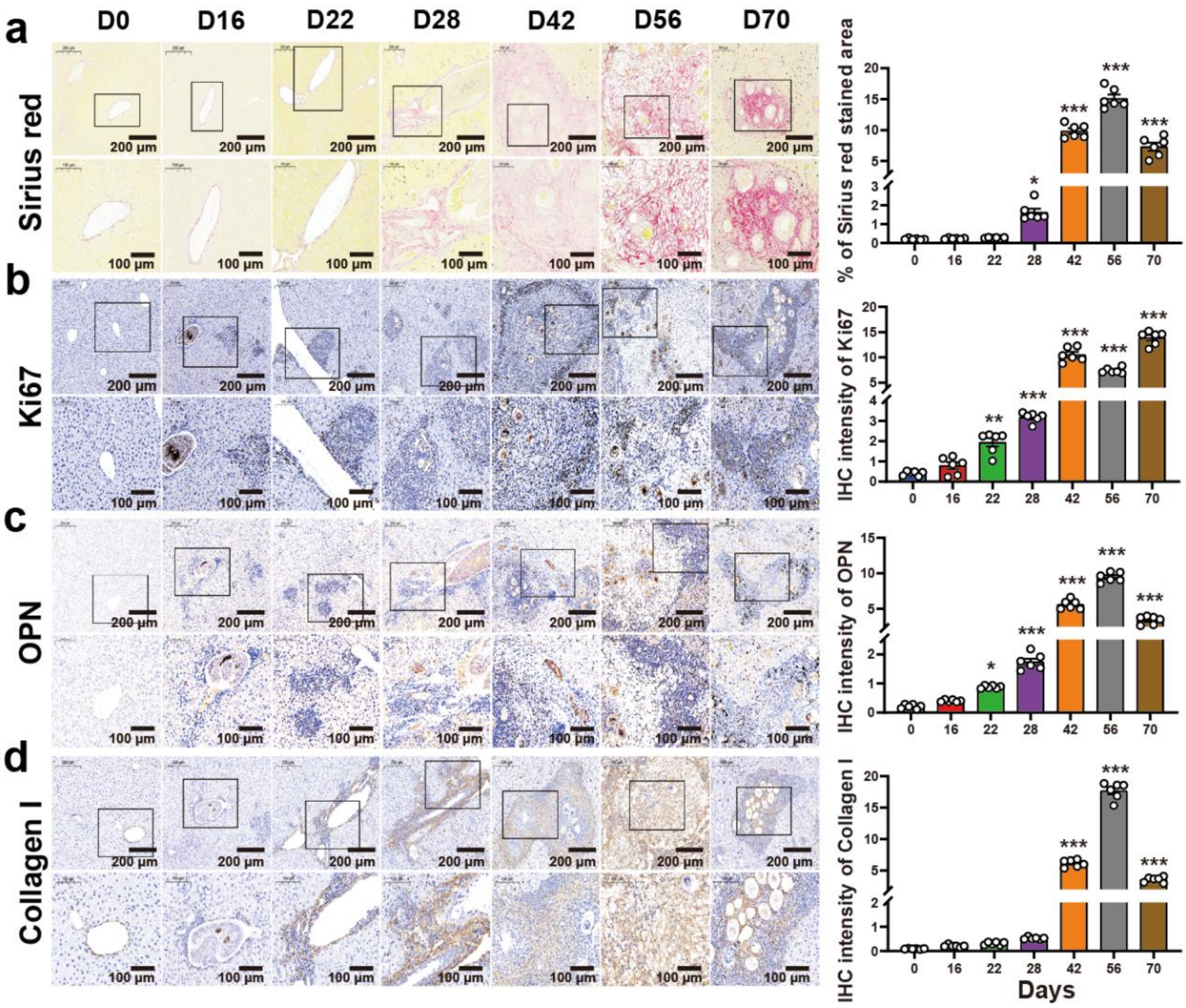

Supplementary Fig. 2. Infection with *S. japonicum* can lead to hepatic fibrosis in mice. (a) Sirius red staining showed collagen depositions of the liver in mice infected with *S. japonicum*. (b-d) The expression of Ki67, Osteopontin (OPN), and Collagen I in mouse livers infected with *S. japonicum*. The highlighted area indicates the positive cells. The positive areas in the livers of non-infected and infected mice were quantitated by Image-Pro Plus software (n = 6 per group). The values were expressed as mean ± SEM. Statistical significance was determined by one-way ANOVA with multiple comparisons. Scale bars: 200 μm (overview), 100 μm (magnified inset). Compared with indicated groups, $*P < 0.05$, $**P < 0.01$, $***P < 0.001$. Source data are provided as a Source Data file. Exact *P* values are provided in the Source Data file.

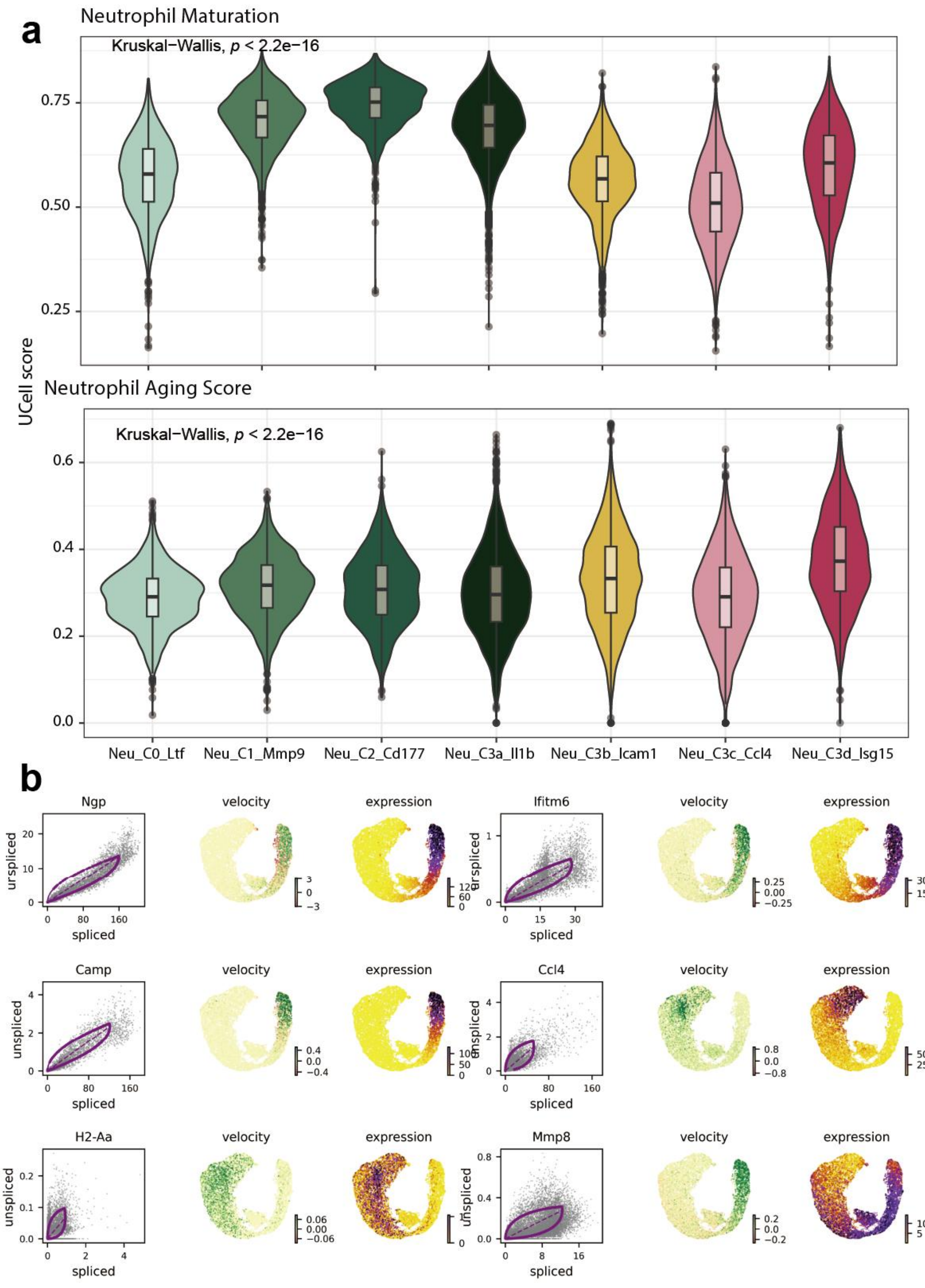

Supplementary Fig. 3. Directional changes of individual cells and intercellular transition processes within neutrophil subsets. (a) Score of neutrophil maturation and degree of aging. (b) Velocity visualization of the top 6 driver genes.

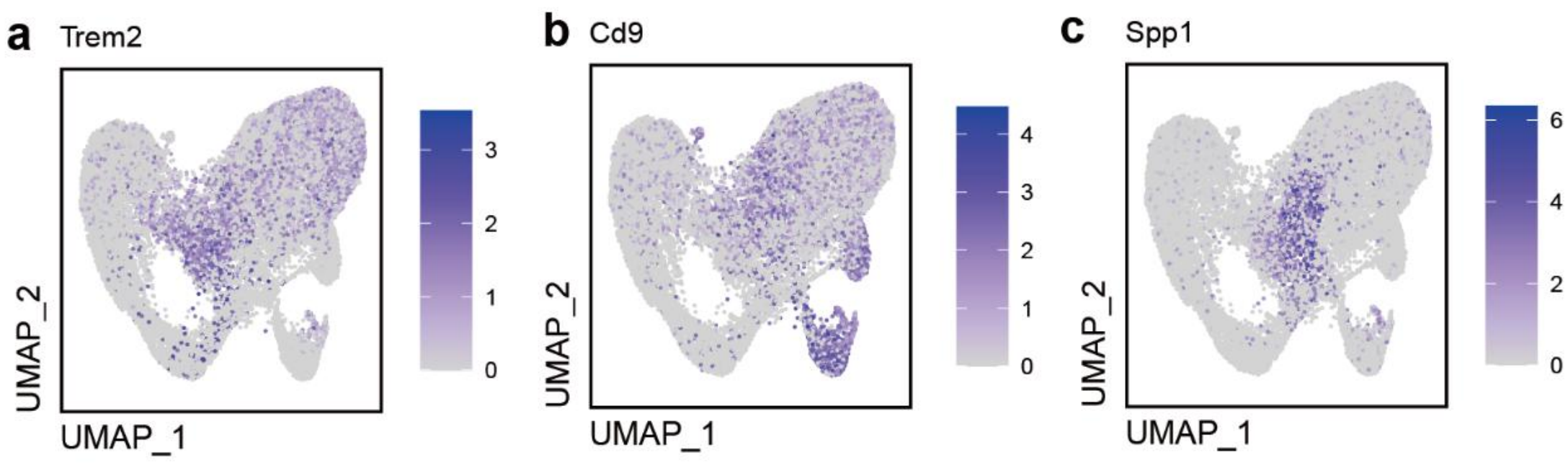

Supplementary Fig. 4. Single-cell characterization of mononuclear phagocyte subsets in the liver during *S. japonicum* infection. UMAP feature plots showing the expression of representative marker genes across mononuclear phagocyte subsets, including Trem2 (a), Cd9 (b), and Spp1 (c). Color intensity indicates normalized expression levels.

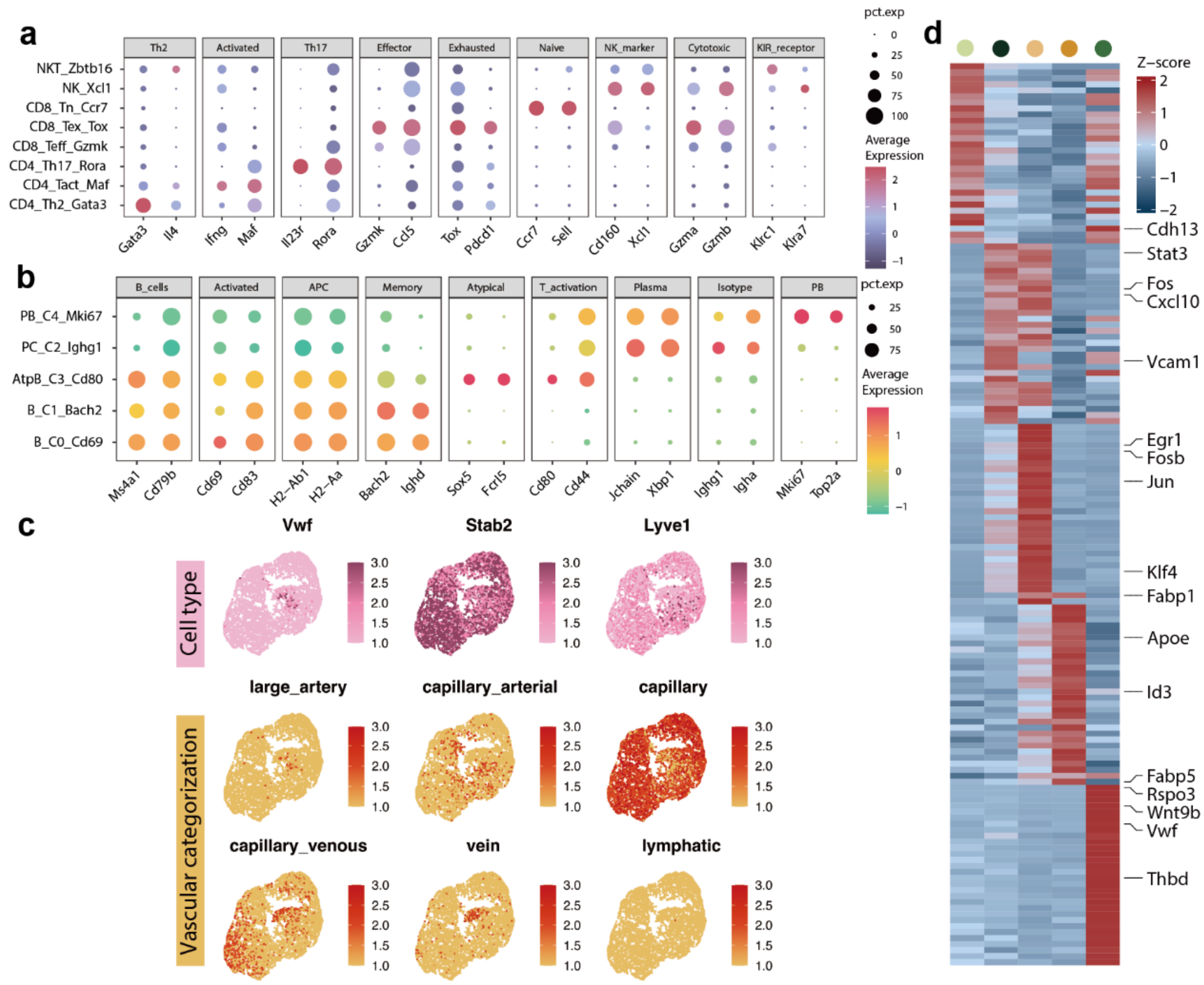

Supplementary Fig. 5. Dynamic alterations in T cells, B cells, and endothelial cells during the progression of liver fibrosis in *S. japonicum*-infected mice. (a) Bubble plots of T cells for the top 2 marker genes expressed for each cluster. (b) Bubble plots of B cells for the top 2 marker genes expressed for each cluster. (c) Subsets of endothelial cells related to the formation of vascular types. (d) Gene expression heatmap (marker gene expressed) in endothelial cells subsets.

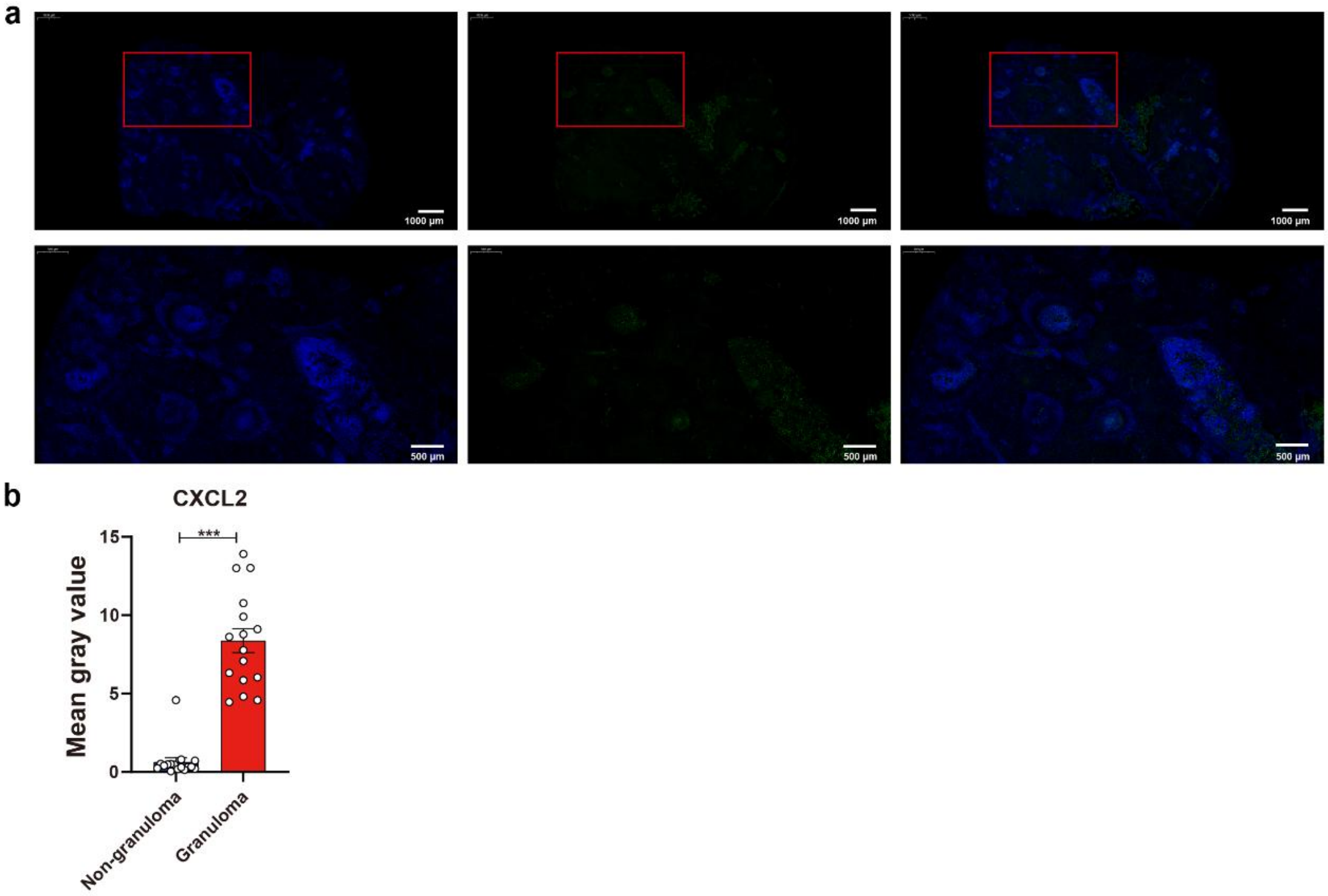

Supplementary Fig. 6. Spatial enrichment of CXCL2 expression within hepatic granulomas during *S. japonicum* infection. (a) Representative immunofluorescence images of CXCL2 expression in liver sections at 42 d.p.i. Nuclei are stained with DAPI (blue), and CXCL2 is shown in green. Upper panels show low-magnification views of whole tissue sections, and lower panels show magnified views of the regions indicated by red boxes. CXCL2 signals are predominantly localized within granulomatous regions. Scale bars, 1000 μm (upper panels) and 500 μm (lower panels). (b) Quantitative analysis of CXCL2 fluorescence intensity. Mean gray values were measured in granulomatous and non-granulomatous regions using ImageJ. Each dot represents one field of view (ROI), with a total of 16 fields analyzed across multiple mice ($n = 16$ per group). Data are presented as mean ± SEM. Statistical significance was determined by two-sided unpaired *t* test. ***$P < 0.001$ compared with the control group. CXCL2 expression is significantly enriched in granulomas compared with non-granulomatous liver tissue. Source data are provided as a Source Data file. Exact *P* values are provided in the Source Data file.

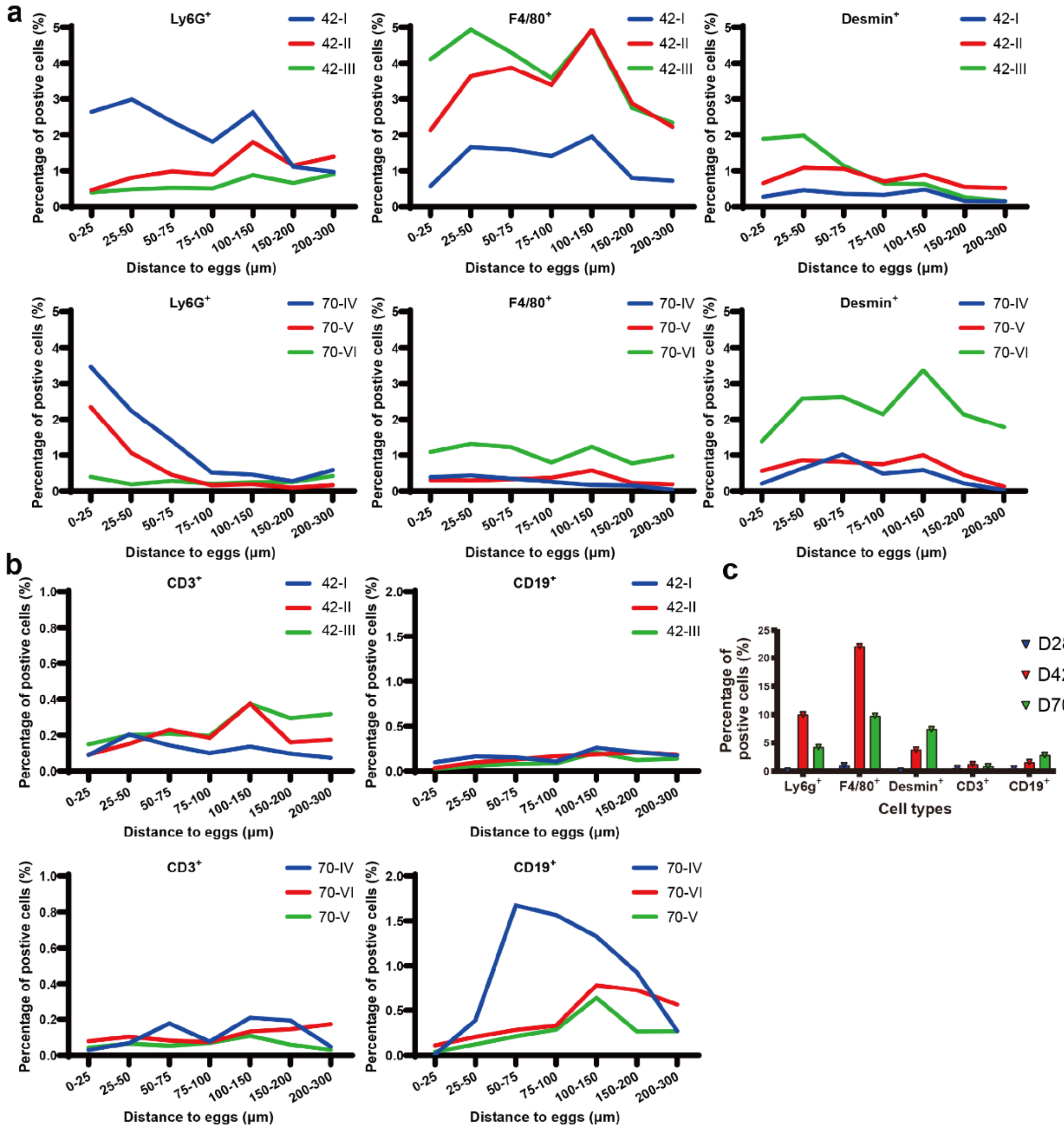

Supplementary Fig. 7. The proportion of Ly6g^{+}, F4/80^{+}, Desmin^{+}, CD3^{+}, and CD19^{+} cells at different distances from eggs. (a) Ly6g^{+}, F4/80^{+}, and Desmin^{+} cells at 42 and 70 d.p.i. (b) CD3^{+} and CD19^{+} cells at 42 and 70 d.p.i. (c) The proportions of Ly6g^{+}, F4/80^{+}, Desmin^{+}, CD3^{+}, and CD19^{+} cells in the liver tissues of mice at 28, 42, and 70 d.p.i.

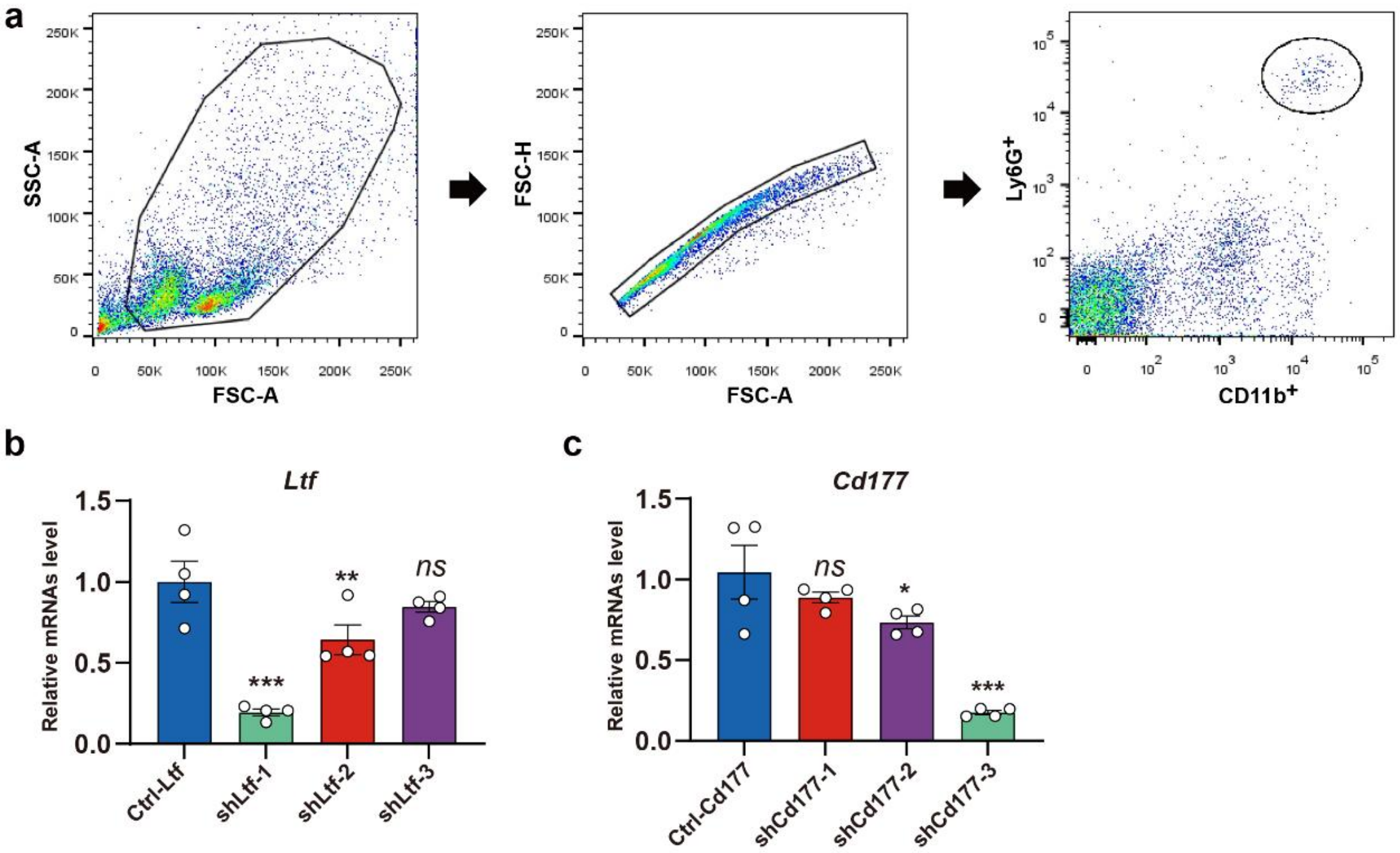

Supplementary Fig. 8. Isolation of hepatic neutrophils and validation of *Ltf* and *Cd177* expression in *S. japonicum*–infected mice. (a) Flow cytometry sorting of Ly6G$^+$/CD11b$^+$ cells (neutrophils). Liver cells were initially gated according to FSC-A and SSC-A parameters to exclude debris, followed by singlet selection using FSC-H versus FSC-A. The target neutrophil population (circled area) was defined as Ly6G$^+$ and CD11b$^+$. The same gating strategy was used for all flow cytometric analyses presented in this study. Relative *Ltf* (b) and *Cd177* (c) mRNA levels in Ly6G$^+$/CD11b$^+$ cells. Data are presented as mean ± SEM, n = 4 per group. Statistical significance was determined by two-sided unpaired *t* test. *$P < 0.05$, **$P < 0.01$, ***$P < 0.001$ compared with the control group. Source data are provided as a Source Data file. Exact *P* values are provided in the Source Data file.

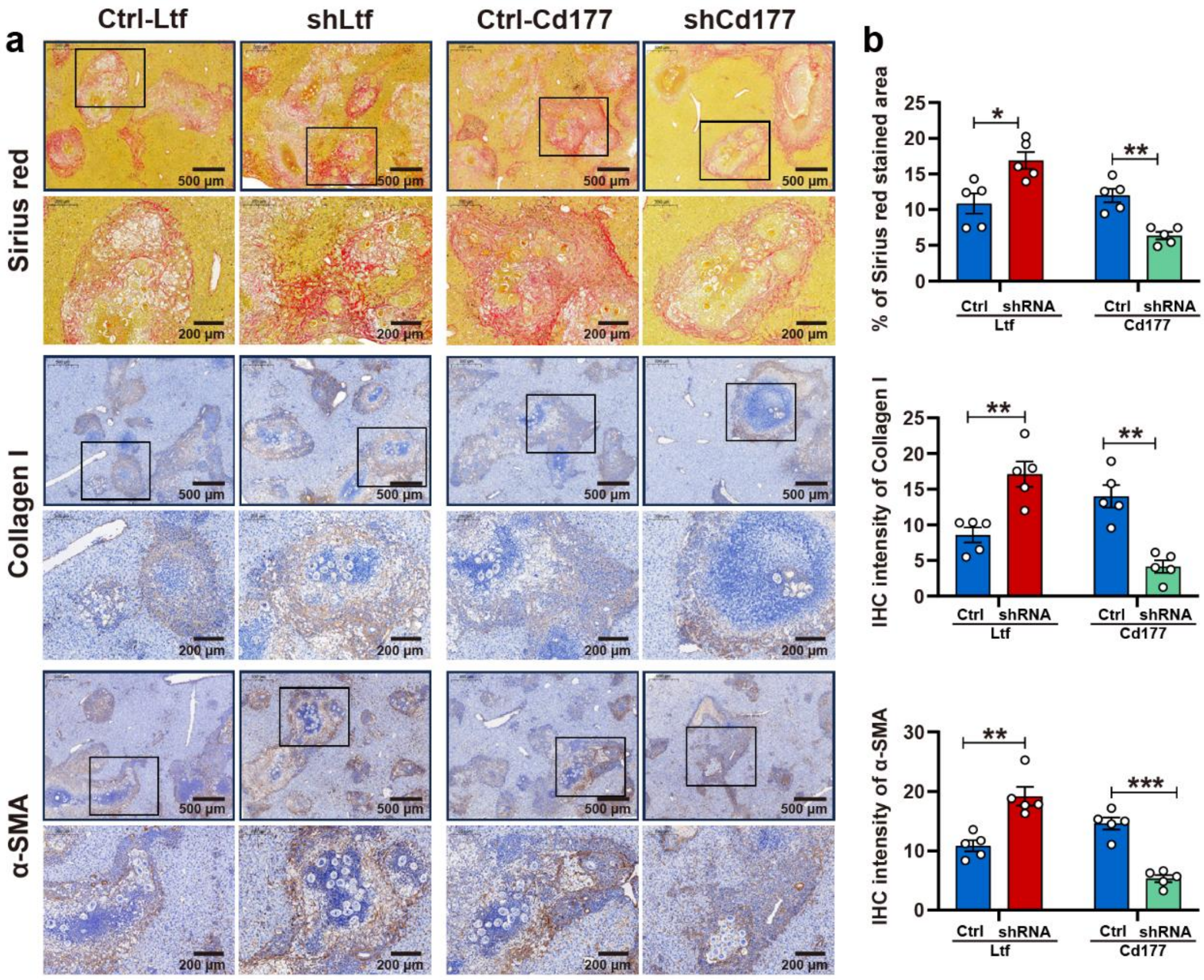

Supplementary Fig. 9. Assessment of fibrosis in *S. japonicum*-infected mice following knockdown of *Ltf* and *Cd177*. (a) Representative images of Sirius red staining, Collagen I, and α-SMA immunohistochemistry. (b) The positive areas in the liver of control and shRNA group were quantitated by Image-Pro Plus software. The values were expressed as mean ± SEM, n = 5 per group. Statistical significance was determined by two-sided unpaired t test. Scale bars: 200 μm (overview), 100 μm (magnified inset). **P* < 0.05, ***P* < 0.01 when compared with control groups. Source data are provided as a Source Data file. Exact *P* values are provided in the Source Data file.

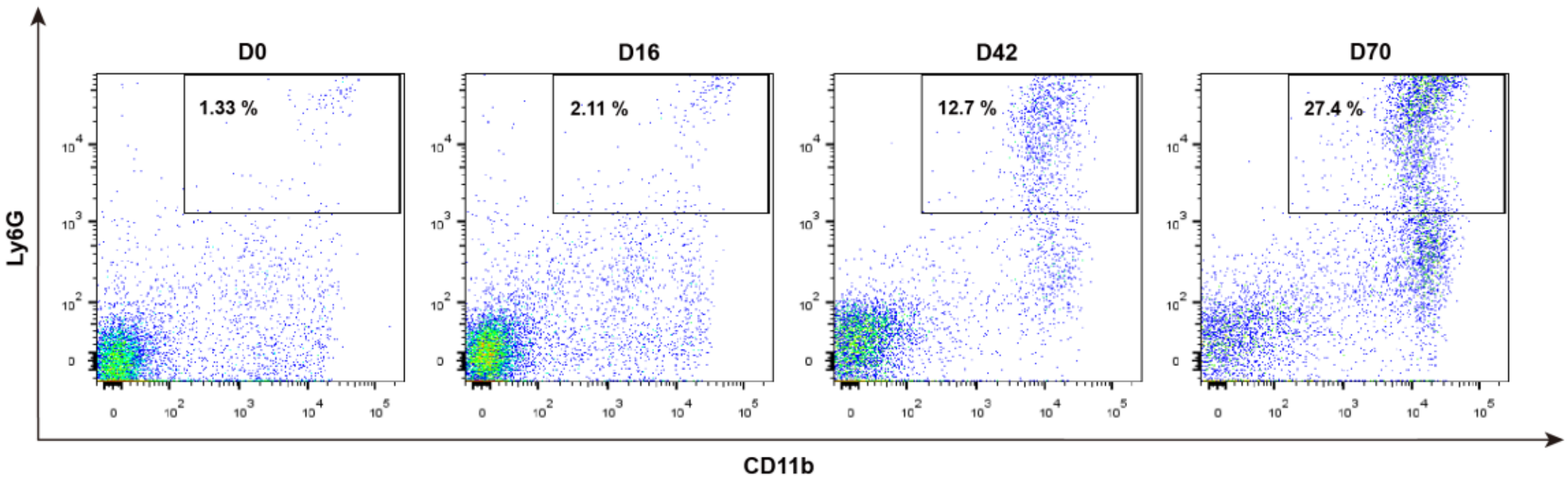

Supplementary Fig. 10. Dynamic expansion of hepatic Ly6G⁺CD11b⁺ neutrophils during *S. japonicum* infection. Representative flow cytometry plots showing Ly6G and CD11b expression in liver cells at D0, D16, D42, and D70 days post-infection. The gated population (upper right quadrant) represents $Ly6G^{+}CD11b^{+}$ neutrophils, and the corresponding percentages are indicated in each panel. Data show a progressive increase in neutrophil frequency over the course of infection.

Supplementary Tables

Supplementary Table 1. Liver granuloma types at different stages of *S. japonicum* infection

Days post-infection	Granuloma type	Granuloma size (mm^2)	Number of granulomas	Granuloma area (mm^2, %)	Number of eggs	Mean eggs per granuloma
**D28**	28-I	0.01–0.12	20	0.88 (100%)	41	2.05 ± 1.28
**D42**	42-I	0.03–0.43	25	3.86 (20.6%)	110	4.40 ± 3.03
	42-II	0.74–1.23	4	4.25 (22.7%)	92	23.00 ± 11.90
	42-III	4.23–6.48	2	10.61 (56.7%)	358	179.00 ± 80.00
**D70**	70-IV	0.08–0.40	7	1.46 (18.5%)	39	5.57 ± 3.96
	70-V	1.51–2.03	2	3.54 (44.9%)	163	81.50 ± 51.50
	70-VI	0.10–0.34	16	2.88 (36.6%)	47	2.94 ± 1.56
