# Supplementary material for: Cellular heterogeneity of hepatic granuloma formation and evolution in murine schistosomiasis japonica
Source: Nat Commun. 2026 Jul 8;17:8697. doi: 10.1038/s41467-026-75112-7 (PMC13490380; doi:10.1038/s41467-026-75112-7)
Supplement: Supplementary file 2 [file 41467_2026_75112_MOESM2_ESM.pdf]

Description of Additional supplementary files

Supplementary Data 1. Differentially expressed marker genes of neutrophil subsets.

Supplementary Data 2. Gene signatures defining functional states of neutrophil and Mono/Marco subsets.

Supplementary Data 3. Differentially expressed marker genes of Mono/Marco subsets.

Supplementary Data 4. Differentially expressed marker genes of endothelial cell subsets.

Supplementary Data 5. Differentially expressed marker genes of all 37 identified sub-populations.
