# Supplementary material for: Cellular heterogeneity of hepatic granuloma formation and evolution in murine schistosomiasis japonica
Source: Nat Commun. 2026 Jul 8;17:8697. doi: 10.1038/s41467-026-75112-7 (PMC13490380; doi:10.1038/s41467-026-75112-7)
Supplement: Supplementary file 4 [file 41467_2026_75112_MOESM9_ESM.pdf]

nature portfolio

Peer Review File

Cellular heterogeneity of hepatic granuloma formation and evolution in murine schistosomiasis japonica

Corresponding Author: Professor Xindong Xu

This file contains all reviewer reports in order by version, followed by all author rebuttals in order by version.

Version 0:

Reviewer comments:

Reviewer #1

(Remarks to the Author)
In this work, the authors have conduced a very thorough analysis of the immunological nature of the granulomatous response to Schistosoma japonicum infection in a mouse model. Specifically, they rely upon single cell RNAseq in order to generate a large single cell atlas of non-parenchymal host cells in livers from infected mice over the course of a S. japonicum infection. This revealed many instances of cellular heterogeneity in a variety of immune populations. The authors then focused in on granulocyte heterogeneity and performed some knockdown experiments to test the function of two genes during infection. They showed that these genes, markers of distinct neutrophil subsets, play opposite roles in the outcome of schistosome infection.

In all, this work provides a great deal of data related to the immune response to Schistosoma japonicum infection, which, as the authors point out, is not as well understood as the response to Schistosoma mansoni infection. As such, it does represent a useful contribution to the field of schistosome immunobiology. Even as a useful contribution, there are a number of shortcomings that should be addressed prior to publication.

Major points:

General

The authors state in the materials and methods that 3 mice are used per time point. This is potentially an issue because the authors also show a survival curve wherein some fraction of animals die midway through the experiment, but every data point following animal death has the same number of replicates. There are also 6 data points in Figures 1E-H, so it is not clear where these numbers come from. Additionally, there is no mention of how many animals are used for the knockdown experiment. The authors need to clearly state how many animals are used in each experiment to allow accurate interpretation of data.

Specific:

Figure 1C: It is not clear what "hepatic wet weight/body weight (%)" means. A plain interpretation would be that the hepatic weight wait constitutes 100% of the body weight of the mouse at 42 days post infection, which is obviously incorrect. The authors should report this as liver weight anyways, as it is impossible to understand whether an increase in this ratio is hepatomegaly or animal weight loss

Minor points:

General

While the data regarding the function of Ltf and Cd177 is fascinating, it seems like studying Cxcl2/Cxcr2 would have tied in better with the data regarding intercellular communication. As it stands, the intercellular communication data is potentially interesting but not supported by any functional data. I don't think this direct connection is necessary to make the manuscript suitable for publication, but I believe it would substantially improve the impact of the manuscript if more of the bioinformatic

analyses were supported with functional data.

Many of the single cell plots appear to have a great deal of unexplored heterogeneity, some of which is addressed in subsequent figures but many of which are largely unexplored. To name a few examples, there are clearly subsets of dividing cells, dendritic cells, B-cells, etc. The authors have already generated a monumental amount of data so fully exploring every cluster isn't feasible, but a higher resolution initial atlas (i.e., one that incorporates all of the subclusters described further on in the manuscript) in combination with the output of FindAllMarkers() from Seurat would enable readers to more closely examine cell populations of interest without requiring the authors to make any more figures.

Specific

Line 62: plexus should not be plural
Line 298: Saying that the "compositional shift likely reflects SEA driven recruitment" is too strong of conclusion for something that the authors did not directly test
Line 500: "indicates" is too strong of a word for something the authors did not directly test
Line 693: Though the authors claim that their work "challenges the conventional view of neutrophils as a homogenous, terminally differentiated population", they extensively rely upon at least two other studies that have already 'challenged' this conventional view in order to describe their granulocyte data. As such, this claim seems to be a bit of an overreach.
Line 702: the authors say that "CXCL2 likely serves as the primary chemokine mediating neutrophil recruitment in models of S. japonicum infection" which they did not show in this work. They should either cite where this is shown or significantly soften their claim given the lack of data directly supporting this.
Line 731: same as line 693
Figure 1F: Readers who are unfamiliar with schistosomes may benefit from the authors labeling the parasites on the histology images.
Figure 1H: IHC intensity score is never defined; is this the IOD?

Reviewer #2

(Remarks to the Author)
The single cell resources presented in this study are a value addition to the knowledge regarding the understanding of hepatic schistosomiasis pathology. A substantial body of high quality data is presented.
Why were these papers not considered regarding schistosome/neutrophil transcriptomics
Li et al Single-cell RNA sequencing reveals a peripheral landscape of immune cells in Schistosomiasis japonica. Parasit Vectors. 2023 Oct 10;16(1):356. I realise this is PBMCs but it should be mentioned and compared.
Chuah, et al . (2014) Defining a Pro-inflammatory Neutrophil Phenotype in Response to Schistosome Eggs. Cellular Microbiology. 16: 1666-77.

Further the zonal observations by immunolocalisation should be compared. and/or referenced to: Chuah, et al (2013) Spatial and Temporal gene expression during Schistosoma japonicum hepatic granuloma formation. Journal of Leukocyte Biology. 94: 353-365.

In figures where SEM is presented please define the n.
Line 196 “likely because severe liver fibrosis interfered with isolating single cells at this stage.” Has this been reported in similar studies of other fibrotic liver pathologies? Similarly what about the observed the deficiency of HSCs?

This recent publication should be compared in regard to single-cell metrics, as well as findings. Watson et al. Spatial transcriptomics of healthy and fibrotic human liver at single-cell resolution. Nature Communications. 2025;16 1:319; doi: 10.1038/s41467-024-55325-4. https://doi.org/10.1038/s41467-024-55325-4. Similarly references 10,11 and 12 within this publication should be considered.
Scale bars needed for Figs 8B,
Line 84 correct “Ccercariae”
Line 851 what lobe was used for liver histo/IHC?
Line 836 how big were mouse groups? The number of biological replicates need to be better communicated
Data Availability was appropriate.

Reviewer #3

(Remarks to the Author)
Summary
The manuscript by Wu et al. investigates temporal changes in granuloma cellularity and transcriptomes after infection with the parasitic helminth, Schistosoma japonicum. The study provides a very deep data set covering the transcriptomic profiles of the many different cell types present in the liver during infection with a focus on neutrophils as well as mononuclear phagocytes (monocytes and macrophages) as these are the major populations. The broad cellular groups are clustered into subsets and temporal changes assessed. From these analyses the authors investigate two distinct molecular signatures in more detail. First, they explore the chemokine network and, in particular, the CXCL2-CXCR2 cross talk by immune subsets. They also demonstrate that CXCL2 is present in a granuloma (Fig 7I). Second, they identified CD177, Ltf and MMP9 as

markers for 3 neutrophil subsets and further explored their location in hepatic granulomas. Using siRNA delivered by AAV8, they knocked down CD177 or Ltf in mice to evaluate how a reduction in these factors affected disease parameters and found that reduction in CD177 appeared to ameliorate disease parameters while a reduction in Ltf exacerbated them.

Overall, the work is interesting and provides valuable insight into the temporal changes that occur in the liver during infections with S. japonicum. It is well presented and clearly written. While the data in Fig 1 are not new (these changes have been known for decades), they are a nice summary for subsequent analyses. One aspect that was surprising was the absence of any reference or discussion of a previous study with a similar approach (Chuah et al. 2013. Spatial and temporal transcriptomics of Schistosoma japonicum-induced hepatic granuloma formation reveals novel roles for neutrophils. JLB. 94: 353–365). This study had some similar findings – identifying the heavy involvement of neutrophils as well as the expression of CXCL2, Ltf and desmin. In my mind, the fact that some of the findings were reported previously does not detract from the value of this work but instead provides an external validation, and this current manuscript certainly describes a much deeper and more complex analysis.

Specific points

1. Line 43: “Cellular localization analysis of the developmental trajectory of granulomas showed that they progressed from the early Ly6GhiF4/80loDesminlo stage, through the developing Ly6GmidF4/80hiDesminmid stage, to the advanced Ly6GloF4/80midDesminhi stage.”

2. Line 58: “Schistosomiasis is the most prevalent helminthic disease, affecting over 250 million people worldwide” By all accounts, Ascaris lumbrcoides is the most prevalent helminth infection with >1 billion infected.

3. Line 97: “…with variations in immune cell composition showing temporal and interspecific variations.” What is meant by ‘interspecific’?

4. Line 110: “…occasionally triggering intraovular immune responses.” Do you mean intravascular”?

5. Line 196: “The lowest cell counts were observed at 70 d.p.i. (3756 and 3761, N=2), likely because severe liver fibrosis interfered with isolating single cells at this stage..” Is it possible that the fibrosis biased the type of cells that were isolated? Was there any assessment of the cell numbers or proportions in the granulomas by flow cytometry or confocal microscopy that confirm the broad scRNAseq clusters?

6. Line 206 and line 697: “The granulocyte cluster was composed of 97.7% neutrophils and approximately 2.3% mast cells, with no eosinophils detected.” The lack of eosinophils in the liver during S. japonicum infection is very interesting. While there are many studies reporting their abundance during infection, there have also been studies where their presence was not noted. In the Discussion, the absence of eosinophils is attributed potentially to SjE16.7. “This species-specific difference suggests that S. japonicum may produce functional factors (e.g., SjE16.7) that recruit neutrophils directly.” However, since there are reports of the presence and absence of eosinophils, a deeper discussion is merited. Otherwise, the generalizability of the findings from this study are impacted.

7. Line 219: “robust granulocyte expansion (predominantly neutrophils) which emerge as key orchestrators…” There is no proof provided that they are "orchestrators" although they look to be heavily involved.

8. Line 366: “Among the CD4+ T cell clusters, the CD4_Th2_Gata2 subset was defined by high Gata2 and IL4 expression, which is consistent with a classical Th2 phenotype.” I believe that you mean GATA-3.

9. Line 513: “To further verify the role of the CXCL2-CXCR2 axis in granuloma formation, we analyzed the distribution of CXCL2 expression in the mouse liver at 42 d.p.i. Immunofluorescence staining results showed that CXCL2 expression was predominantly concentrated within hepatic granulomas (Figure 7I),..” While it is good to have an anecdotal confirmation that CXCL2 is expressed in the hepatic granulomas, was there any deep, quantitative assessment of CXCL2 expression spatially or temporally?

10. Figure 7G: “(G) Expression dynamics of key chemokines and cytokines across the course of infection. Panels 1–7: chemokines produced by seven immune cell types.” How were these genes selected? For example, CXCL2 is not shown for monocytes or macrophages, but seemed to be expressed. Why was it not shown?

11. Line 542: “We observed significant heterogeneity among hepatic granulomas both across different infection stages and within the same infection stage (Table 1).” How many mice were used for these studies?

12. Figure 10: “(A) Ltf and Cd177 expression in different cell clusters.” What timepoint is shown in panel A?

13. Section 2.10: The use of AAV8 to deliver siRNA looks very promising but it must be acknowledged that it is not specific to neutrophils. While i.v.-injected AAV will be taken up by monocytes and neutrophils, it can transduce many cell types and thus the effect of knocking down CD177 or Ltf could be mediated by any of these cells, not necessarily neutrophils. Moreover, could the authors comment on how the single injection can enable the knockdown of CD177 and Ltf in neutrophils for over 4 weeks, given their lifespan of 24 hours?

14. Line 668 and line 716: “These results indicate that these two neutrophil subsets play opposing roles in S. japonicum egg–induced granuloma pathogenesis: the CD177+ subset acts as a driver of granulomatous inflammation, whereas the

LTF+ subset functions to suppress this process.” and “Following CD177 knockdown, the number of CD177+ neutrophils decreased and this was accompanied by a reduction in granuloma size and fibrosis severity, which suggested that CD177+ neutrophils may promote the progression of egg-induced inflammatory granuloma formation and associated fibrosis.” One cannot conclude that all these effects are mediated by neutrophil subsets. That has not been shown. What has been shown is that siRNA knocked down the expression of CD177 and LTF and that the siRNA led to distinct pathological changes. But, whether these two neutrophil subsets are involved has not been shown. It is just as possible that other cells (immune or non-immune) mediated the pathological effects. More caution must be used in interpreting these findings.

15. Did the knockdown of CD177 or Ltf cause any change in adult worm numbers or egg production? If so, could that explain the changes to liver pathology?

Version 1:

Reviewer comments:

Reviewer #1

(Remarks to the Author)
Overall, the authors sufficiently addressed this reviewer's concerns. This reviewer would still prefer Fig 1D presented as a percent rather than permille, as (in this reviewer's experience), percent is far more commonly used. Nevertheless, the data are interpretable as presented so it doesn't need to be changed. As such, this reviewer believes that the manuscript is now suitable for publication.

Reviewer #2

(Remarks to the Author)
Thank you for fully addressing my feedback and questions.

Reviewer #3

(Remarks to the Author)
The manuscript by Wu et al. has been vastly improved by the modifications suggested by the reviewers. Almost all my queries have been addressed save one relating to Table 1. This table as well as the corresponding Figure 8c & d and text require just a few modifications to more accurately reflect the data shown. The key issue is that the catagorisation into “early”, “developing” and “advanced” granulomas does not reflect the data in Figure 8c & d. Specifically:

1. The use of Ia and Ib for day 72 does not make sense. Why not use 72-IV for this type of "advanced" granuloma. It is the only stage where these are detected and the use of IV seems more logical.

2. In the text, three types of granulomas are listed but 42-I granulomas are not included. Is 42-I not an "early granuloma"?

3. Also, looking at the Ly6G-F4/80-Desmin expression in Fig 8c, there is no consistency in the I or Ia "early" granuloma pattern. The expression of all these markers is very low at day 28 while day 42-I is high-mid-low and 72-Ia is mid-low-low. Given these patterns, day 28 looks to be very different from the other timepoints (certainly not Ly6Ghigh) with day 40 and 72 being more similar to each other.

Greater clarity is needed in the granuloma types and patterns.

Open Access This Peer Review File is licensed under a Creative Commons Attribution 4.0 International License, which permits use, sharing, adaptation, distribution and reproduction in any medium or format, as long as you give appropriate credit to the original author(s) and the source, provide a link to the Creative Commons license, and indicate if changes were made.
In cases where reviewers are anonymous, credit should be given to 'Anonymous Referee' and the source.
The images or other third party material in this Peer Review File are included in the article's Creative Commons license, unless indicated otherwise in a credit line to the material. If material is not included in the article's Creative Commons license and your intended use is not permitted by statutory regulation or exceeds the permitted use, you will need to obtain permission directly from the copyright holder.
To view a copy of this license, visit https://creativecommons.org/licenses/by/4.0/

Detailed Response to Reviewers

Manuscript ID: NCOMMS-25-105447

Title: Cellular heterogeneity of *Schistosoma japonicum* egg-induced hepatic granulomas in mice: insights into granuloma formation and evolution

We sincerely thank all reviewers for their thoughtful and constructive comments as well as valuable suggestions, which have greatly helped us enhance the rigor, clarity, and completeness of our manuscript. We have carefully addressed all the concerns raised by the reviewers and revised the manuscript systematically and point by point. The detailed responses to each comment are provided below, and the corresponding revisions in the manuscript are highlighted in red.

Response to Reviewer #1

In this work, the authors have conducted a very thorough analysis of the immunological nature of the granulomatous response to Schistosoma japonicum infection in a mouse model. Specifically, they rely upon single cell RNAseq in order to generate a large single cell atlas of non-parenchymal host cells in livers from infected mice over the course of a S. japonicum infection. This revealed many instances of cellular heterogeneity in a variety of immune populations. The authors then focused in on granulocyte heterogeneity and performed some knockdown experiments to test the function of two genes during infection. They showed that these genes, markers of distinct neutrophil subsets, play opposite roles in the outcome of schistosome infection.

In all, this work provides a great deal of data related to the immune response to *Schistosoma japonicum* infection, which, as the authors point out, is not as well understood as the response to *Schistosoma mansoni* infection. As such, it does represent a useful contribution to the field of schistosome immunobiology. Even as a useful contribution, there are a number of shortcomings that should be addressed prior to publication.

Major points:

General

The authors state in the materials and methods that 3 mice are used per time point. This is

potentially an issue because the authors also show a survival curve wherein some fraction of animals die midway through the experiment, but every data point following animal death has the same number of replicates. There are also 6 data points in Figures 1E-H, so it is not clear where these numbers come from. Additionally, there is no mention of how many animals are used for the knockdown experiment. The authors need to clearly state how many animals are used in each experiment to allow accurate interpretation of data.

We sincerely appreciate the reviewer's careful comments and constructive suggestions regarding animal numbers and experimental design. We fully acknowledge the need to clarify animal usage in each experiment for accurate data interpretation, and we have revised the manuscript accordingly, with all details consistent with the experimental design described in the "**Animals, parasites and experimental design**" section in Methods.

First, regarding the survival curve and body weight monitoring (Figure 1B and C): These data were obtained from an independent cohort of 25 mice, including 5 uninfected control mice and 20 infected mice. One infected mouse was excluded from subsequent analyses due to failure to develop a patent infection (no adult worms or eggs detected at necropsy). Survival status was monitored daily from 0 to 70 d.p.i., and body weight was measured weekly, with the survival curve generated based on this specific cohort.

Second, regarding serum liver function detection and histopathological evaluation (including the data points in Figures 1E–H): These experiments used a second independent cohort of 50 mice. Six uninfected mice were euthanized at day 0 to serve as baseline controls; the remaining 44 mice were infected with S. japonicum, with six mice euthanized at each indicated time point (16, 22, 28, 42, 56, and 70 d.p.i.). Additionally, eight infected mice were reserved as spares to replace individuals that died unexpectedly during the experiment, ensuring that each time point maintained the planned sample size. The data points in Figures 1E–H were derived from this cohort, and we have clarified the origin of these data points in the revised manuscript.

Third, regarding liver wet weight measurement and scRNA-seq analysis: A third independent cohort of 40 mice was used. At day 0 and each post-infection time point (16, 22, 28, 42, 56, and 70 d.p.i.), five mice were euthanized: three for liver wet weight measurement, and two for in situ perfusion and subsequent isolation of liver non-parenchymal cells (NPCs) for scRNA-seq. Five additional infected mice were reserved as spares to address unexpected attrition.

Finally, regarding the knockdown experiment: We have supplemented the detailed animal number in the Materials and Methods section. Specifically, five mice were used for the knockdown experiment, ensuring transparency and reproducibility of all experimental procedures.

All animal grouping and sample size details are now clearly stated in both the Materials and Methods section and the corresponding figure legends, consistent with the experimental design, to facilitate accurate interpretation of the data.

Specific

Figure 1C: It is not clear what "hepatic wet weight/body weight (%)" means. A plain interpretation would be that the hepatic weight wait constitutes 100% of the body weight of the mouse at 42 days post infection, which is obviously incorrect. The authors should report this as liver weight anyways, as it is impossible to understand whether an increase in this ratio is hepatomegaly or animal weight loss.

In this study, the presented hepatic wet weight/body weight ratio was calculated as (liver weight / body weight) × 1000, expressed as per mille (‰) rather than percentage (%). The raw data of individual mouse liver wet weight and body weight have been listed in detail in the supplementary table.

Minor points:

General

While the data regarding the function of Ltf and Cd177 is fascinating, it seems like studying Cxcl2/Cxcr2 would have tied in better with the data regarding intercellular communication. As it stands, the intercellular communication data is potentially interesting but not supported by any functional data. I don't think this direct connection is necessary to make the manuscript suitable for publication, but I believe it would substantially improve the impact of the manuscript if more of the bioinformatic analyses were supported with functional data.

We sincerely appreciate the reviewer’s insightful comment and valuable suggestion, which is

instrumental in strengthening the mechanistic depth and impact of our study. We fully agree that validating the CXCL2-CXCR2 axis, a key intercellular communication pathway identified in our bioinformatic analyses, would create a more cohesive link between our single-cell transcriptional data and functional outcomes, enhancing the rigor of our findings. In line with this constructive advice, we plan to conduct in-depth investigations in our future work to elucidate the specific molecular mechanisms by which the CXCL2-CXCR2 signaling axis mediates neutrophil recruitment and activation, which will further consolidate the physiological significance of our intercellular communication analysis.

Many of the single cell plots appear to have a great deal of unexplored heterogeneity, some of which is addressed in subsequent figures but many of which are largely unexplored. To name a few examples, there are clearly subsets of dividing cells, dendritic cells, B-cells, etc. The authors have already generated a monumental amount of data so fully exploring every cluster isn't feasible, but a higher resolution initial atlas (i.e., one that incorporates all of the subclusters described further on in the manuscript) in combination with the output of FindAllMarkers () from Seurat would enable readers to more closely examine cell populations of interest without requiring the authors to make any more figures.

We thank the reviewer for this constructive suggestion. The sub-clustering and annotation of B cell subsets have already been presented in Figure 5. In addition, we further performed sub-clustering and re-annotation of dendritic cells (DCs). Two DC subclusters were identified based on canonical mouse DC markers: cDC1_Xcr1 (high *Xcr1* and *Cadm1*), specialized in cross-presentation and enriched during early infection; and pDC_Bst2 (high *Bst2*, *Siglech*, and *Tcf4*) that peaked during the late phase, suggesting involvement in chronic inflammation as reported in *S. mansoni*.

Regarding the dividing cell cluster, we did not perform further sub-clustering because these cells are predominantly in the G2/M phase of the cell cycle and express a wide range of markers corresponding to multiple lineages. They are likely proliferating progenitors or activated cells derived from various immune populations, rather than a distinct functional cell type. Therefore, we focused our sub-clustering analysis on non-cycling, lineage-defined populations.

As recommended by the reviewer, we have now integrated all subclusters into a single high-resolution UMAP, which is presented in the revised **Figure 5**. Meanwhile, the Results

section 2.5 has been revised and reorganized to match the updated clustering results and UMAP visualization.

Specific

Line 62: plexus should not be plural

Corrected.

Line 298: Saying that the "compositional shift likely reflects SEA driven recruitment" is too strong of conclusion for something that the authors did not directly test

We revised the sentence that reads as: "This temporal association raises the hypothesis that egg deposition generates an inflammatory milieu triggered by soluble egg antigens (SEA). The microenvironment may promote the recruitment and local expansion of precursor and immature neutrophils to hepatic granulomas, where these subsets may help modulate egg-induced pathology (e.g., via ROS production, NET formation, or proteolytic enzyme release)."

Line 500: "indicates" is too strong of a word for something the authors did not directly test

The sentence has been revised to: "Notably, the Mono_C1_Cxcl2 cluster emerged specifically following egg deposition. We speculate that this subset plays a critical role in initiating neutrophil recruitment."

Line 693: Though the authors claim that their work "challenges the conventional view of neutrophils as a homogenous, terminally differentiated population", they extensively rely upon at least two other studies that have already 'challenged' this conventional view in order to describe their granulocyte data. As such, this claim seems to be a bit of an overreach.

We have revised the corresponding sentence as follows: "We identified seven transcriptionally and functionally distinct neutrophil subsets, further highlighting the extensive heterogeneity of neutrophils."

Line 702: the authors say that "CXCL2 likely serves as the primary chemokine mediating

neutrophil recruitment in models of S. japonicum infection" which they did not show in this work. They should either cite where this is shown or significantly soften their claim given the lack of data directly supporting this.

We have softened the claim. Line 702 has been revised to: "Our data suggest that CXCL2 may play an important role in mediating neutrophil recruitment during *S. japonicum* infection, although whether it acts as the primary chemokine driving this process requires further investigation."

Line 731: same as line 693

We appreciate the reviewer's suggestion. This sentence has been revised to: "Collectively, our results highlight that neutrophils comprise a heterogeneous population and form functionally distinct, spatially regulated subsets during *S. japonicum* infection".

Figure 1F: Readers who are unfamiliar with schistosomes may benefit from the authors labeling the parasites on the histology images

We have revised Figure 1F, in which schistosome eggs and schistosomula are now circled with dashed lines in the histological images. The corresponding figure legend has also been updated accordingly to improve readability for readers unfamiliar with schistosomes.

Figure 1H: IHC intensity score is never defined; is this the IOD?

We apologize for the lack of definition. The IHC intensity score refers to the integrated optical density (IOD), quantified using Image-Pro Plus 6.0 software. This definition has been added to Method: "IHC intensity score represents integrated optical density (IOD), with higher values indicating stronger α-SMA expression (quantified via Image-Pro Plus 6.0 software)."

Response to Reviewer #2

The single cell resources presented in this study are a value addition to the knowledge regarding the understanding of hepatic schistosomiasis pathology. A substantial body of high quality data is presented.

Why were these papers not considered regarding schistosome/neutrophil transcriptomics.

Li et al Single-cell RNA sequencing reveals a peripheral landscape of immune cells in Schistosomiasis japonica. Parasit Vectors. 2023 Oct 10;16(1):356. I realise this is PBMCs but it should be mentioned and compared.

Chuah, et al . (2014) Defining a Pro-inflammatory Neutrophil Phenotype in Response to Schistosome Eggs. Cellular Microbiology. 16: 1666-77.

Further the zonal observations by immunolocalisation should be compared. and/or referenced to: Chuah, et al (2013) Spatial and Temporal gene expression during Schistosoma japonicum hepatic granuloma formation. Journal of Leukocyte Biology. 94: 353-365.

We thank the reviewer for pointing out these three important papers and for the constructive suggestion to include them in our manuscript. We have now carefully read and incorporated all three references into the revised manuscript, as detailed below.

Regarding Li et al. (2023):

This scRNA-seq study on human PBMCs is relevant to our work, as it identified expanded CXCR2^{+} NKT cells in advanced schistosomiasis patients. We have cited this paper in the revised Discussion, we state: "This chemokine axis may also operate in other immune cell types. A recent scRNA-seq study of PBMCs from patients with schistosomiasis japonica reported that CXCR2^{+} NKT cells are expanded in advanced disease and exhibit a pro-inflammatory signature"

Regarding Chuah et al. (2014):

We have cited this paper in the revised Discussion to support our finding that neutrophils co-express CXCL2 and CXCR2, and to highlight the conserved ability of schistosome eggs to drive neutrophil inflammatory responses across species (human *in vitro* vs. mouse *in vivo*). Specifically, we state: "In addition, S. japonicum eggs can directly stimulate human neutrophils

to upregulate CXCL2 expression, highlighting the potent capacity of schistosome eggs to drive neutrophil inflammatory responses.*"*

Regarding Chuah et al. (2013):

This pioneering LMM-based transcriptomic study first described a dual neutrophil phenotype (pro-inflammatory core, reparative periphery) within zonal granuloma regions. We have now cited it in the Discussion and directly compared our findings: "Earlier LMM-based transcriptomics described a dual neutrophil phenotype (pro-inflammatory core, reparative periphery) in *S. japonicum* granulomas. Here, we further reveal prominent and functionally meaningful spatial zonation of neutrophil subsets within early granulomas."

In figures where SEM is presented please define the n.

We have now reviewed all figures that present data as mean ± SEM. The number of biological replicates (n) has been explicitly defined in each corresponding figure legend. These revisions have been made throughout the manuscript.

Line 196 "likely because severe liver fibrosis interfered with isolating single cells at this stage." Has this been reported in similar studies of other fibrotic liver pathologies? Similarly what about the observed the deficiency of HSCs?

We sincerely thank the reviewer for raising these important technical points regarding the impact of fibrosis on single-cell isolation and the low recovery of HSCs. Our speculation that severe liver fibrosis interferes with efficient single-cell recovery is indeed well supported by previous studies of fibrotic liver pathologies. As demonstrated by MacParland et al. (2018) and Andrews et al. (2022), enzymatic and mechanical dissociation of human liver tissue significantly biases cell composition; this bias is exacerbated in fibrotic livers where harsher digestion protocols are necessary to break down dense extracellular matrix, thereby reducing cell yield and viability [1,2]. The same technical limitations also explain the low number of HSCs detected in our scRNA-seq data. HSCs are exceptionally fragile due to their stellate morphology and lipid-rich cytoplasm, making them prone to rupture during dissociation. Thus, the underrepresentation of HSCs reflects a well-recognized technical challenge, not their biological absence.

We have now explicitly stated these limitations in the revised Discussion. Future studies using single-nucleus RNA-seq or HSC enrichment prior to sequencing will better capture these cells.

Reference：

1. MacParland SA, Liu JC, Ma XZ, et al. Single cell RNA sequencing of human liver reveals distinct intrahepatic macrophage populations. Nat Commun. 2018;9:4383.
2. Andrews TS, Atif J, Liu JC, et al. Single-cell, single-nucleus, and spatial RNA sequencing of the human liver identifies cholangiocyte and mesenchymal heterogeneity. Hepatol Commun. 2022;6(5):821–840.

This recent publication should be compared in regard to single-cell metrics, as well as findings. Watson et al. Spatial transcriptomics of healthy and fibrotic human liver at single-cell resolution. Nature Communications. 2025;16 1:319; doi: 10.1038/s41467-024-55325-4. https://doi.org/10.1038/s41467-024-55325-4. Similarly references 10,11 and 12 within this publication should be considered.

We thank the reviewer for directing our attention to this important recent publication by Watson et al. (2025) and its references 10, 11, and 12. We have now carefully reviewed these studies and incorporated them into our revised manuscript.

Regarding Watson et al. (2025):

This study applied MERFISH spatial transcriptomics combined with single-nucleus RNA-seq (snRNA-seq) to map healthy and fibrotic human liver at single-cell resolution. The authors explicitly note that snRNA-seq bypasses the dissociation bias inherent to scRNA-seq and is compatible with frozen and fibrotic specimens, enabling comprehensive profiling of all major hepatic cell types. These approaches represent a powerful complement to conventional scRNA-seq for studying fibrotic liver diseases.

In the revised Discussion, we have therefore added a forward-looking statement that future investigations combining snRNA-seq and spatial transcriptomics will help circumvent dissociation-induced bias and better preserve fragile cells such as HSCs.

Regarding reference 10 (MacParland et al., 2018) and reference 12 (Andrews et al., 2022):

These studies, as discussed above, provide direct evidence for the technical limitations of scRNA-seq in recovering mesenchymal cells from human liver. We have cited them in the revised Discussion to support our technical limitation statement.

Regarding reference 11 (Ramachandran et al., 2019):

This study identified a $TREM2^+CD9^+$ macrophage subpopulation in human cirrhotic liver and in a mouse carbon tetrachloride (CCl_4)-induced fibrosis model that expresses high levels of CD9, TREM2, and SPP1, and is associated with hepatic fibrosis. We examined the expression of these genes in our scRNA-seq data and found that our Macro_C2_$Arg1^+$ (M2-like) macrophages also exhibit high expression of these markers (Fig. S3 in the revised manuscript). This cross-species conservation across human cirrhosis, murine CCl_4-induced hepatic fibrosis, and *S. japonicum*-induced hepatic fibrosis indicates that a similar pro-fibrotic macrophage subset is activated across different etiologies of liver fibrosis.

All suggested references have been added to the reference list. We thank the reviewer again for these valuable suggestions, which have substantially improved our manuscript.

Scale bars needed for Figs 8B,

Scale bars have been added to the revised Figure 8B, 9A-F, and 10G-J.

Line 84 correct "Ccercariae"

Corrected

Line 851 what lobe was used for liver histo/IHC?

The left lateral lobe was used for all histopathological and immunohistochemical analyses. The sentence has been revised : " Liver samples from the left lateral lobe were cut into 10 mm × 10 mm × 1 mm pieces and fixed in 4% paraformaldehyde following standard protocols and

subsequently sliced into 4-μm-thick continuous sections."

Line 836 how big were mouse groups? The number of biological replicates need to be better communicated.

We thank the reviewer for pointing out the lack of clarity regarding group sizes and biological replicates. To address this, we have substantially revised the "Animals, parasites and experimental design" subsection in the Materials and Methods. The revised text now clearly describes the number of mice used in each independent cohort, the allocation to experimental groups, and the number of biological replicates per time point and per assay. All sample sizes (n values) are also explicitly indicated in the corresponding figure legends.

Data Availability was appropriate.

We appreciate the reviewer's confirmation of appropriate data availability.

Response to Reviewer #3

Summary

The manuscript by Wu et al. investigates temporal changes in granuloma cellularity and transcriptomes after infection with the parasitic helminth, Schistosoma japonicum. The study provides a very deep data set covering the transcriptomic profiles of the many different cell types present in the liver during infection with a focus on neutrophils as well as mononuclear phagocytes (monocytes and macrophages) as these are the major populations. The broad cellular groups are clustered into subsets and temporal changes assessed. From these analyses the authors investigate two distinct molecular signatures in more detail. First, they explore the chemokine network and, in particular, the CXCL2-CXCR2 cross talk by immune subsets. They also demonstrate that CXCL2 is present in a granuloma (Fig 7I). Second, they identified CD177, Ltf and MMP9 as markers for 3 neutrophil subsets and further explored their location in hepatic granulomas. Using siRNA delivered by AAV8, they knocked down CD177 or Ltf in mice to evaluate how a reduction in these factors affected disease parameters and found that reduction in CD177 appeared to ameliorate disease parameters while a reduction in Ltf exacerbated them. Overall, the work is interesting and provides valuable insight into the temporal changes that occur in the liver during infections with S. japonicum. It is well presented and clearly written. While the data in Fig 1 are not new (these changes have been known for decades), they are a nice summary for subsequent analyses. One aspect that was surprising was the absence of any reference or discussion of a previous study with a similar approach (Chuah et al. 2013. Spatial and temporal transcriptomics of Schistosoma japonicum-induced hepatic granuloma formation reveals novel roles for neutrophils. JLB. 94: 353–365). This study had some similar findings – identifying the heavy involvement of neutrophils as well as the expression of CXCL2, Ltf and desmin. In my mind, the fact that some of the findings were reported previously does not detract from the value of this work but instead provides an external validation, and this current manuscript certainly describes a much deeper and more complex analysis.

We thank the reviewer for bringing this important prior study to our attention and for the fair assessment that its findings do not detract from the value of our work but rather provide external validation. We apologize for the original omission. In the revised manuscript, we have now cited and discussed Chuah et al. in the Discussion. Specifically, we note that their pioneering LMM-based transcriptomic study first described a dual neutrophil phenotype (pro-inflammatory core, reparative periphery) within zonal granuloma regions, and detected elevated expression of *Cxcl2*, *Ltf*, and *Desmin*. We then elaborate how our single-cell resolution

and functional knockdown experiments extend these earlier observations by identifying the specific subsets ($CD177^+$ vs. LTF^+) that underlie the dual roles proposed by Chuah et al. Thus, while Chuah et al. established the foundation, our work provides a deeper, higher-resolution, and functionally validated framework.

Specific points

1. Line 43: "Cellular localization analysis of the developmental trajectory of granulomas showed that they progressed from the early $Ly6G^{hi}F4/80^{lo}Desmin^{lo}$ stage, through the developing $Ly6G^{mid}F4/80^{hi}Desmin^{mid}$ stage, to the advanced $Ly6G^{lo}F4/80^{mid}Desmin^{hi}$ stage."

We thank the reviewer for highlighting this sentence, which summarizes a key finding of our study regarding the developmental trajectory of hepatic granulomas.

The three stages - early ($Ly6G^{hi}F4/80^{lo}Desmin^{lo}$), developing ($Ly6G^{mid}F4/80^{hi}Desmin^{mid}$), and advanced ($Ly6G^{lo}F4/80^{mid}Desmin^{hi}$) - were defined based on quantitative multiplex immunofluorescence analysis of granulomas at different infection time points (Figure 8). The classification reflects the dynamic changes in the relative proportions of neutrophils (Ly6G), macrophages (F4/80), and activated HSCs (Desmin) as granulomas mature and progress toward fibrosis.

2. Line 58: "Schistosomiasis is the most prevalent helminthic disease, affecting over 250 million people worldwide" By all accounts, Ascaris lumbrcoides is the most prevalent helminth infection with >1 billion infected.

The line has been revised to: "Schistosomiasis is one of the most prevalent and devastating helminthic diseases, affecting over 250 million people worldwide."

3. Line 97: "…with variations in immune cell composition showing temporal and interspecific variations." What is meant by 'interspecific'?

The intended meaning is "interspecies". The sentence has been revised to: "Schistosome granulomas recruit a wide variety of immune cells, including eosinophils, neutrophils, monocytes, macrophages, lymphocytes, and epithelioid cells, with variations in immune cell

composition differing across time and species."

4. Line 110: "…occasionally triggering intraovular immune responses." Do you mean intravascular"?

We do not mean intravascular. We intended to describe immune responses occurring inside the schistosome eggs. The sentence has been revised to: "However, neutrophils continue to be the predominant cell type within advanced *S. japonicum* granulomas, frequently degranulating upon contact with eggshells and occasionally triggering immune responses inside the schistosome eggs."

5. Line 196: "The lowest cell counts were observed at 70 d.p.i. (3756 and 3761, N=2), likely because severe liver fibrosis interfered with isolating single cells at this stage." Is it possible that the fibrosis biased the type of cells that were isolated? Was there any assessment of the cell numbers or proportions in the granulomas by flow cytometry or confocal microscopy that confirm the broad scRNAseq clusters?

We thank the reviewer for this insightful question. We fully agree that severe liver fibrosis at 70 d.p.i. may introduce bias in the recovery of specific cell types during single-cell isolation. To address this concern, we compared three datasets to verify the reliability of our scRNA-seq results and clarify potential technical bias:

1) scRNA-seq (Figure 2C): This dataset showed a progressive increase in neutrophil proportions from 0 to 70 d.p.i., with the highest percentage observed at 70 d.p.i.

2) Flow cytometry of dissociated liver NPCs (Supplementary Figure 9 in the revised manuscript): Performed in an independent study covering 0, 16, 42, and 70 d.p.i., this analysis confirmed the trend observed in scRNA-seq, demonstrating a time-dependent increase in the proportion of $Ly6G^{+}$ neutrophils.

3) Multiplex immunofluorescence on liver tissue sections (Figure 8C): In contrast to the dissociated cell methods, in situ imaging revealed that the proportion of $Ly6G^{+}$ neutrophils within granulomas was higher at 42 d.p.i. than at 70 d.p.i.

The discrepancy between dissociated cell techniques (scRNA-seq and flow cytometry) and in situ imaging can be explained by the heterogeneous composition of the fibrotic liver at 70 d.p.i. At this late infection stage, the liver contains distinct types of granulomas:

(1) Predominant advanced, fibrotic granulomas (Ly6G^{lo}F4/80^{mid}Desmin^{hi}): These granulomas are embedded in dense extracellular matrix, contain very few neutrophils, and are resistant to enzymatic dissociation.

(2) Minor newly formed, early granulomas (Ly6G^{hi}F4/80^{lo}Desmin^{lo}): Continuously generated by persistent egg laying of adult worms, these granulomas are neutrophil-rich, structurally loose, and easily dissociable.

During tissue processing, easily dissociable early granulomas are preferentially disrupted, releasing abundant neutrophils into the single-cell suspension. In contrast, cells within advanced fibrotic granulomas are largely retained in undigested tissue fragments and thus underrepresented. This technical bias leads to an overestimation of neutrophil proportions in scRNA-seq and flow cytometry datasets at 70 d.p.i., compared to the true cellular composition of the entire liver.

We therefore agree with the reviewer that in situ imaging (multiplex immunofluorescence, Figure 8C) provides a more accurate reflection of the true cellular composition within granulomas, particularly in the context of advanced liver fibrosis. This consideration has been added to the limitations section in Discussion.

6. Line 206 and line 697: “The granulocyte cluster was composed of 97.7% neutrophils and approximately 2.3% mast cells, with no eosinophils detected.” The lack of eosinophils in the liver during S. japonicum infection is very interesting. While there are many studies reporting their abundance during infection, there have also been studies where their presence was not noted. In the Discussion, the absence of eosinophils is attributed potentially to SjE16.7. “This species-specific difference suggests that S. japonicum may produce functional factors (e.g., SjE16.7) that recruit neutrophils directly.” However, since there are reports of the presence and absence of eosinophils, a deeper discussion is merited. Otherwise, the generalizability of the findings from this study are impacted.

We thank the reviewer for this insightful comment. In the revised Discussion, we have substantially expanded our analysis of the absence of eosinophils in our dataset to address the concern regarding generalizability.

Specifically, we now:

(1) Acknowledge the well-established species difference: *S. japonicum* granulomas are neutrophil-dominated, whereas *S. mansoni* granulomas are eosinophil-dominated.

(2) Describe the dual role of neutrophils in *S. japonicum* infection – they drive acute hepatic necrosis and fibrosis yet also exert regulatory functions, as evidenced by augmented granulomas in neutrophil-depleted mice.

(3) Cite the parasite-derived factor SjE16.7 that directly recruits neutrophils and induces NET formation, which may indirectly limit eosinophil recruitment through microenvironmental competition.

(4) Contrast this with the mechanism in *S. mansoni*, where a secreted chemokine-binding protein (smCKBP) selectively blocks neutrophil chemoattractant CXCL8 while sparing eosinophil chemoattractant CCL11, thereby favoring eosinophil infiltration.

These additions, detailed in the revised Discussion, demonstrate that the absence of eosinophils in our model is consistent with a substantial body of literature on species-specific granuloma composition and does not compromise the generalizability of our findings. We thank the reviewer for prompting this deeper contextualization.

7. Line 219: "robust granulocyte expansion (predominantly neutrophils) which emerge as key orchestrators…" There is no proof provided that they are "orchestrators" although they look to be heavily involved.

The sentence has been revised to: "This restructuring is accompanied by robust granulocyte expansion (predominantly neutrophils), which are highly involved in shaping the inflammatory response and the subsequent pathological progression during *S. japonicum* infection."

8. Line 366: "Among the CD4+ T cell clusters, the CD4_Th2_Gata2 subset was defined by high Gata2 and IL4 expression, which is consistent with a classical Th2 phenotype." I believe that you mean GATA-3.

We appreciate the correction. The signature transcription factor for Th2 cells is GATA-3, not GATA-2.

9. Line 513: "To further verify the role of the CXCL2-CXCR2 axis in granuloma formation, we analyzed the distribution of CXCL2 expression in the mouse liver at 42 d.p.i. Immunofluorescence staining results showed that CXCL2 expression was predominantly concentrated within hepatic granulomas (Figure 7I)," While it is good to have an anecdotal confirmation that CXCL2 is expressed in the hepatic granulomas, was there any deep, quantitative assessment of CXCL2 expression spatially or temporally?

We thank the reviewer for this insightful comment. The current immunofluorescence assay was originally designed to clarify the spatial localization of CXCL2 in the liver at 42 d.p.i., a key time point for mature granuloma formation, and we did not conduct a temporal dynamic analysis in this part.

To further address the reviewer's concern and provide quantitative spatial evaluation, we have now supplemented quantitative analysis of CXCL2 expression based on whole-slide tissue imaging (added as Figure S5). We systematically compared the CXCL2 fluorescence intensity within granulomas with that in the adjacent normal liver parenchyma. Quantitative results confirmed that CXCL2 signal intensity was significantly higher in granuloma regions than in the surrounding hepatic tissues, which solidifies our original qualitative observation that CXCL2 is predominantly enriched in hepatic granulomas.

10. Figure 7G: "(G) Expression dynamics of key chemokines and cytokines across the course of infection. Panels 1–7: chemokines produced by seven immune cell types." How were these genes selected? For example, CXCL2 is not shown for monocytes or macrophages, but seemed to be expressed. Why was it not shown?

We apologize for the unclear description and insufficient explanation of gene selection in the original figure legend, which has caused confusion.

Figure 7G aimed to illustrate the temporal expression dynamics of representative chemokines and cytokines at the bulk level with all cell populations integrated across the infection time course, focusing on the signature chemokine axes governing immune cell recruitment. The genes in each panel were selectively chosen based on well-established cell-type-specific canonical chemokine-receptor signaling modules to systematically delineate the recruitment mechanism of distinct immune subsets. The Ccl2/Ccl7/Ccl8/Ccl12 cluster represents the canonical CCR2-axis ligands, which primarily govern monocyte and macrophage recruitment. The Cxcl1/Cxcl2/Cxcl3/Cxcl5/Cxcl15 panel constitutes the core CXCR1/2-axis chemokines that specifically mediate neutrophil chemotaxis and infiltration. Other panels were similarly defined by representative signature cytokines or chemokines corresponding to individual immune cell lineages.

To improve readability and clarity, we have thoroughly revised Figure 7G, Figure 7H, and the corresponding figure legends.

11. Line 542: "We observed significant heterogeneity among hepatic granulomas both across different infection stages and within the same infection stage (Table 1)." How many mice were used for these studies?

We thank the reviewer for the question. The data in Table 1 were derived from the whole-slide multiplex immunofluorescence images shown in Figure 8A.

12. Figure 10: "(A) Ltf and Cd177 expression in different cell clusters." What timepoint is shown in panel A?

We thank the reviewer for the question. The expression data shown in Figure 10A are derived from the combined scRNA-seq dataset integrating all seven time points (0, 16, 22, 28, 42, 56, and 70 d.p.i.), which provides a global view of Ltf and Cd177 expression across different cell clusters.

13. Section 2.10: The use of AAV8 to deliver siRNA looks very promising but it must be acknowledged that it is not specific to neutrophils. While i.v.-injected AAV will be taken up by monocytes and neutrophils, it can transduce many cell types and thus the effect of knocking

down CD177 or Ltf could be mediated by any of these cells, not necessarily neutrophils. Moreover, could the authors comment on how the single injection can enable the knockdown of CD177 and Ltf in neutrophils for over 4 weeks, given their lifespan of 24 hours?

We fully agree with the reviewer that intravenously injected AAV8 can transduce multiple cell types and lacks absolute neutrophil specificity. While AAV8 has high tropism for hepatocytes, its transduction of immune cells including monocytes and neutrophils has been reported. We therefore cannot exclusively attribute the observed phenotypic changes to neutrophil-specific knockdown of *Cd177* or *Ltf*. Accordingly, we have revised the manuscript throughout, removing overstated causal claims and adopting neutral descriptions of the functional alterations induced by AAV8-mediated knockdown. Related qPCR data have also been rearranged between the main and supplementary figures for appropriate presentation.

Regarding the sustained knockdown effect over 4 weeks despite the short lifespan of circulating neutrophils:

Although circulating neutrophils have a short half-life, those recruited to inflamed tissue microenvironments can undergo phenotypic reprogramming and exhibit markedly prolonged survival (1-2). In addition, the liver fibrosis increases AAV8 transduction efficiency in non-parenchymal immune cells (3). Moreover, hepatocyte-derived exosomes can deliver RNAi molecules to newly recruited neutrophils, sustaining gene silencing even in cells not directly transduced by AAV8 (4-5). Collectively, these mechanisms explain the prolonged functional effects observed in our 4-week experiment.

Nevertheless, we acknowledge the limitation of the non-neutrophil-specific AAV8 system; future work using neutrophil-specific conditional knockout mice will be necessary to definitively dissect the intrinsic roles of $CD177^{+}$ and LTF^{+} neutrophils in schistosomiasis-associated granuloma and fibrosis progression.

To avoid overinterpretation, we have carefully revised the descriptions throughout Results sections 2.10 and 2.11, and further incorporated this limitation and related mechanistic interpretation into the Discussion section.

Reference：

1. Ng MSF, Kwok I, Tan L, et al. Deterministic reprogramming of neutrophils within tumors. Science. 2024;383(6679):eadf6493. doi: 10.1126/science.adf6493
2. Zhang F, Xia Y, Su J, et al. Neutrophil diversity and function in health and disease. Signal Transduct Target Ther. 2024;9(1):343. doi: 10.1038/s41392-024-02049-y
3. Ferriero R, et al. Impact of liver fibrosis on AAV-mediated gene transfer to mouse hepatocytes. Nat Commun. 2025;16:2118. doi: 10.1038/s41467-025-56978-5
4. Pan Q, Ramakrishnaiah V, Henry S, et al. Hepatic cell-to-cell transmission of small silencing RNA can extend the therapeutic reach of RNA interference (RNAi). Gut. 2012;61(9):1330-1339. doi: 10.1136/gutjnl-2011-300449
5. Chen L, Chen R, Kemper S, Brigstock DR. Pathways of production and delivery of hepatocyte exosomes. J Cell Commun Signal. 2018;12(1):343-357. doi: 10.1007/s12079-017-0421-7

14. Line 668 and line 716: “These results indicate that these two neutrophil subsets play opposing roles in S. japonicum egg–induced granuloma pathogenesis: the CD177+ subset acts as a driver of granulomatous inflammation, whereas the LTF+ subset functions to suppress this process.” and “Following CD177 knockdown, the number of CD177+ neutrophils decreased and this was accompanied by a reduction in granuloma size and fibrosis severity, which suggested that CD177+ neutrophils may promote the progression of egg-induced inflammatory granuloma formation and associated fibrosis.” One cannot conclude that all these effects are mediated by neutrophil subsets. That has not been shown. What has been shown is that siRNA knocked down the expression of CD177 and LTF and that the siRNA led to distinct pathological changes. But, whether these two neutrophil subsets are involved has not been shown. It is just as possible that other cells (immune or non-immune) mediated the pathological effects. More caution must be used in interpreting these findings.

We thank the reviewer for this important comment, which is closely related to the previous concern regarding AAV8 specificity. As detailed in our response to comment #13, we have thoroughly revised the manuscript to address this issue. Specifically, we have deleted causal statements that directly attribute pathological changes to $CD177^{+}$ or LTF^{+} neutrophils in Results sections 2.10 and 2.11. We now neutrally state that CD177 or Ltf knockdown attenuated or exacerbated the pathological outcomes, respectively, without overinterpreting and assigning these effects specifically to neutrophils alone. In the Discussion, we have also reframed our conclusions to acknowledge that further studies using neutrophil-specific conditional knockout

mice are required to define the direct roles of these neutrophil subsets.

15. Did the knockdown of CD177 or Ltf cause any change in adult worm numbers or egg production? If so, could that explain the changes to liver pathology?

We thank the reviewer for this important question. We did not directly enumerate adult worm pairs or total egg production in the liver following *Cd177* or *Ltf* knockdown. However, we adopted an alternative analytical approach to exclude the possibility that variations in egg burden might account for the observed differences in liver pathology.

Specifically, for each liver tissue slide, we quantified egg numbers and calculated granuloma area per egg instead of relying solely on total granuloma area. This normalization effectively corrects for potential variability in egg deposition across individual mice and experimental groups. Using this normalized method, we found that *Cd177* knockdown significantly reduced, while *Ltf* knockdown markedly increased, granuloma area per egg relative to control mice (Figure 11H). These findings indicate that the altered pathological outcomes are not attributable to differences in egg production or overall egg burden, but rather reflect genuine modulation of the host granulomatous response by CD177 and LTF.

We have updated Figure 11H and incorporated the explanation into the revised Results 2.11 section.

Conclusion

We have carefully addressed all the comments and suggestions from the reviewers, which significantly improve the quality of our manuscript. We hope the revised manuscript meets the publication standards of the journal. We would like to thank the reviewers again for their valuable contributions to the improvement of our work.

Response to Reviewers' Comments

Reviewer #1 (Remarks to the Author):

Overall, the authors sufficiently addressed this reviewer's concerns. This reviewer would still prefer Fig 1D presented as a percent rather than permille, as (in this reviewer's experience), percent is far more commonly used. Nevertheless, the data are interpretable as presented so it doesn't need to be changed. As such, this reviewer believes that the manuscript is now suitable for publication.

Response: We have revised Fig. 1D (Supplementary Fig.1d in the revised manuscript) and Fig. 10c to present the data as percent (%) instead of permille (‰). We truly appreciate your time and effort in guiding us through the revision process.

Reviewer #2 (Remarks to the Author):

Thank you for fully addressing my feedback and questions.

Response: Thank you very much for your time, effort and constructive feedback throughout the review process.

Reviewer #3 (Remarks to the Author):

The manuscript by Wu et al. has been vastly improved by the modifications suggested by the reviewers. Almost all my queries have been addressed save one relating to Table 1. This table as well as the corresponding Figure 8c & d and text require just a few modifications to more accurately reflect the data shown. The key issue is that the catagorisation into "early", "developing" and "advanced" granulomas does not reflect the data in Figure 8c & d. Specifically:

1. The use of Ia and Ib for day 72 does not make sense. Why not use 72-IV for this type of "advanced" granuloma. It is the only stage where these are detected and the use of IV seems more logical.

Response: We fully agree that the previous Ia/Ib nomenclature for day 70 granulomas was confusing and lacked logical consistency. We have therefore completely revised and unified the nomenclature system for all granuloma subtypes throughout the manuscript.

The new standardized nomenclature is as follows:

1. Day 28: 28-I
2. Day 42: 42-I, 42-II, 42-III
3. Day 70: 70-IV, 70-V, 70-VI

As you correctly pointed out, using 70-IV, 70-V, and 70-VI more logically reflects that these are the most advanced stages of granuloma development detected only at the later time point.

2. In the text, three types of granulomas are listed but 42-I granulomas are not included. Is 42-I not an "early granuloma"?

Response: This question will be addressed together with Comment 3.

3. Also, looking at the Ly6G-F4/80-Desmin expression in Fig 8c, there is no consistency in the I or Ia "early" granuloma pattern. The expression of all these markers is very low at day 28 while day 42-I is high-mid-low and 72-Ia is mid-low-low. Given these patterns, day 28 looks to be very different from the other timepoints (certainly not Ly6Ghigh) with day 40 and 72 being more similar to each other.

Greater clarity is needed in the granuloma types and patterns.

Response: Thank you very much for raising these important questions, which allow us to clarify the rationale behind our granuloma classification system and figure presentation.

We apologize for any confusion caused by our previous text. Type 42-I is indeed the representative of early granulomas. As described in the revised manuscript, we have completely redefined the evolutionary trajectory of schistosome-induced hepatic granulomas. We delineated three core developmental stages: early granulomas (Ly6G^{hi}F4/80^{lo}Desmin^{lo}, represented by type 42-I), developing granulomas (Ly6G^{mid}F4/80^{hi}Desmin^{mid}, represented by type 42-II), and advanced granulomas (Ly6G^{lo}F4/80^{mid}Desmin^{hi}, represented by type 70-VI) (Fig. 7d).

Regarding the other observed subtypes:

1. Type 28-I was excluded from the core classification because it represents atypical lesions induced by immature schistosome eggs. As you correctly noted, these lesions have an unstable cellular composition with very low expression of all three markers, which is distinct from the typical early granuloma phenotype induced by mature eggs.

2. Type 70-IV (previously designated as 70-Ia) exhibits a cellular profile nearly identical to type 42-I, corresponding to early granulomas formed around newly deposited mature eggs at the late infection stage. Therefore, it is categorized under the early granuloma phenotype rather than being designated as an independent core stage.

3. Types 42-III and 70-V are lesions characterized by extensive egg deposition and frequent confluence, which preclude accurate quantitative analysis of individual cellular populations.

For Fig. 8c, representative images were derived from single-egg granulomas wherever feasible to show the full morphological structure of individual lesions. For types 42-III and 70-V, which frequently form confluent lesions, we carefully selected representative individual granulomas that accurately reflect the overall cellular distribution patterns of these subtypes.

We have updated all relevant sections of the manuscript, figures, and figure legends to clearly present this classification rationale and ensure complete consistency throughout the text. (Line 486-532)
